# The nucleardatapy toolkit for simple access to experimental nuclear data, astrophysical observations, and theoretical predictions

**DOI:** 10.1140/epja/s10050-025-01760-w

**Published:** 2026-02-02

**Authors:** Jérôme Margueron, Christian Drischler, Mariana Dutra, Stefano Gandolfi, Alexandros Gezerlis, Guilherme Grams, Sébastien Guillot, Rohit Kumar, Sudhanva Lalit, Odilon Lourenço, Rahul Somasundaram, Ingo Tews, Isaac Vidaña

**Affiliations:** 1 International Research Laboratory on Nuclear Physics and Astrophysics, Michigan State University and CNRS, East Lansing, MI 48824 USA; 2 Institute of Nuclear and Particle Physics (INPP), Ohio University, Athens, OH 45701 USA; 3 Facility for Rare Isotope Beams, Michigan State University, East Lansing, MI 48824 USA; 4 Departamento de Física e Laboratório de Computação Científica Avançada e Modelamento (Lab-CCAM), Instituto Tecnológico de Aeronáutica, DCTA, São José dos Campos, SP 12228-900 Brazil; 5 Theoretical Division, Los Alamos National Laboratory, Los Alamos, NM 87545 USA; 6 Department of Physics, University of Guelph, Guelph, ON N1G 2W1 Canada; 7 Institut für Physik und Astronomie, Universität Potsdam, Haus 28, Karl-Liebknecht-Str. 24/25, 14476 Potsdam, Germany; 8 IRAP, CNRS, 9 avenue du Colonel Roche, BP 44346, 31028 Toulouse Cedex 4, France; 9 Université de Toulouse, CNES, UPS-OMP, 31028 Toulouse, France; 10 Istituto Nazionale di Fisica Nucleare, Sezione di Catania, Dipartimento di Fisica e Astronomia “Ettore Majorana”, Università di Catania, Via Santa Sofia 64, 95123 Catania, Italy

## Abstract

Systematic comparisons across theoretical predictions for the properties of dense matter, nuclear physics data, and astrophysical observations (also called meta-analyses) are performed. Existing predictions for symmetric nuclear and neutron matter properties are considered, and they are shown in this paper as an illustration of the present knowledge. Asymmetric matter is constructed assuming the isospin asymmetry quadratic approximation. It is employed to predict the pressure at twice saturation energy-density based only on nuclear-physics constraints, and we find it compatible with the one from the gravitational-wave community. To make our meta-analysis transparent, updated in the future, and to publicly share our results, the Python toolkit nucleardatapy is described and released here. Hence, this paper accompanies nucleardatapy, which simplifies access to nuclear-physics data, including theoretical calculations, experimental measurements, and astrophysical observations. This Python toolkit is designed to easily provide data for: (i) predictions for uniform matter (from microscopic or phenomenological approaches); (ii) correlation among nuclear properties induced by experimental and theoretical constraints; (iii) measurements for finite nuclei (nuclear chart, charge radii, neutron skins or nuclear incompressibilities, etc.) and hypernuclei (single particle energies); and (iv) astrophysical observations. This toolkit provides data in a unified format for easy comparison and provides new meta-analysis tools. It will be continuously developed, and we expect contributions from the community in our endeavor.

## Program Summary and Specifications

Program title: nucleardatapy, version 1.0

Licensing provisions: CC BY-NC-ND 4.0

Programming language: Python.

Repository: nucleardatapy on GitHub (public), see also Ref. [1].

Documentation: direct link, see also Ref. [2].

Tutorials: direct link, see also Ref. [3].

Description of problem: Nuclear physics and observational data for dense nuclear matter are dispersed across various sources. They may be stored in different formats, requiring the reconstruction of quantities, which might differ from one author to another. Meta-analyses are limited by access to a large number of predictions.

Method of solution: The nucleardatapy toolkit simplifies access to nuclear-physics data by collecting them in a single repository and providing the community with a simple Python toolkit, containing reconstructed quantities in a unified and transparent way. It also makes it easy to manipulate data further as well as to perform meta-analyses.

Additional comments: The Python toolkit employed to create the figures presented in this paper is open-source, see Sect. 2 for details on the installation and use of the toolkit, and more information can also be available from the GitHub repository, see Ref. [1]. Documentation and tutorials are provided as well. Possible issues as well as further extensions can be suggested from the GitHub Issue option, or alternatively, could be discussed directly with the authors of this paper.

## Introduction

Dense nuclear matter is a system instrumental for the understanding of compact stars, such as neutron stars, and high-energy astrophysical phenomena they are involved in, such as binary neutron star mergers. However, it is difficult to directly measure its properties or to isolate its components from a nuclear experiment [4, 5] or an astrophysical observation [6, 7]. In finite nuclei, for instance, dense matter alone does not define their properties, but finite-size contributions, which also reflect the properties of the nuclear interaction, such as its finite range and its momentum-dependence, need to be considered. In neutron stars, it contributes together with leptons to the equation of state (EoS).

Phase transitions in the crust, where forming non-uniform clusters is energetically favorable, or in the core, where new degrees of freedom are expected above a given density, have also to be considered [8]. It is, therefore, impossible to isolate dense nuclear matter from the other effects in the system under study. Instead, in a first step it is more appropriate to compare the global modeling of the system of interest (nuclei, hypernuclei, or neutron stars) with existing data. In the second step, theory is then employed to disentangle the contribution of dense nuclear matter from others.

For such a program, it has become common to perform Bayesian analyses, coupled to Markov-Chain Monte-Carlo sampling of the model parameter space, to link nuclear matter properties with experimental and/or astrophysical data and estimate uncertainties [9, 10]. For instance, collections of scientific libraries are publicly available and can be employed for model parameter exploration [11–13]. In such analyses, the constraints from fundamental approaches as well as from nuclear experiments are crucial to assess the quality of the models. These constraints are, therefore, commonly employed, but they are not systematically provided in a form that is easy to manipulate. In addition, experimental results and theoretical calculations are often available in different formats and references, making comparisons difficult. Since data plays a crucial role in assessing the quality of the models, it is important to furnish a source of data, checked by authors and by the community, which is easily accessible and improved by user feedback. The nucleardatapy toolkit is aimed to be such a community-driven tool. Hence, feedback from users is encouraged and will be fully considered. The motivation for nucleardatapy is to facilitate and simplify the sharing of data in the nuclear and nuclear-astrophysics communities.

The nucleardatapy toolkit collects data that can be useful to calibrate low-energy nuclear models, such as energy-density functionals (EDFs). These EDF^1^ models are expected to reproduce at least nuclear binding energies and charge radii, which are present in the nucleardatapy toolkit, but one could also be interested in comparing model predictions, for example, for the isoscalar giant monopole resonance (ISGMR), the correlations between $E_\textrm{sym}$ and $L_\textrm{sym}$, as well as between $K_\textrm{sat}$ and $Q_\textrm{sat}$, and many others (empirical parameters such as $E_\textrm{sym}$, $L_\textrm{sym}$, $K_\textrm{sat}$, and $Q_\textrm{sat}$ are defined in Sect. 3.12). Additionally, microscopic ab initio and phenomenological calculations of uniform matter are provided by nucleardatapy. Finally, astrophysical data related to neutron star properties are provided in the toolkit. They are complementary to the new repository for astrophysical observations CompARE [14].

The nucleardatapy toolkit also simplifies meta-analyses, by collecting and providing results from different approaches in a single format. The present study suggests several of these meta-analyses, and one that is particularly interesting is the prediction for the pressure at twice saturation energy-density and its comparison with the results from the gravitational-wave community. Furthermore, the nucleardatapy toolkit constructs the EoS for the ground state of uniform nuclear matter at $\beta$-equilibrium for the models predicting nuclear properties in symmetric and neutron matter, and assuming a simple approximation to describe asymmetric matter, see Sect. 8 for more details. A larger sample of EoSs, including finite temperature and non-uniform matter in the crust, is provided by the CompOSE repository [15], or the stellar collapse website [16].

The present paper is divided into the following sections: we begin with the installation of the toolkit in Sect. 2. In Sect. 3 we first review the constraints from microscopic and phenomenological approaches for symmetric and neutron matter (in some cases, asymmetric matter is also provided), e.g., the energy per particle, pairing gaps, empirical parameters, Landau parameters as well as the experimental constraints from heavy-ion collisions. We then show correlation diagrams for $K_\textrm{sat}$-$Q_\textrm{sat}$ and $E_\textrm{sym}$-$L_\textrm{sym}$ in Sect. 4. We review the constraints from finite nuclei in Sect. 5, e.g., nuclear masses, binding energies, two-neutron separation energies, odd-even mass staggering, charge radii, and neutron skins. The constraints from hypernuclei are reviewed in Sect. 6 for single and double $\varLambda$ and single $\varXi ^-$ hypernuclei. We address the neutron star crust and the related crust module in Sect. 7, asymmetric matter at beta-equilibrium in Sect. 8, and astrophysical data in Sect. 9. Our conclusions are presented in Sect. 10.

## Installation notes for the toolkit

The toolkit is written in Python, and once installed, the library is designed to be imported from everywhere, including Google colab. To install the toolkit, launch the following command from a terminal on your computer (or on Google Colab): 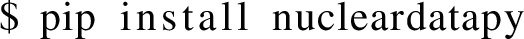 The toolkit is available for direct download on the pypi website. Once installed, one can access this package from any Python script. If you are using a mobile device, details on installing and using the nucleardatapy toolkit are given in Appendix B. In all cases, one must import the toolkit in the usual way in Python: 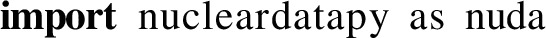 We also import the numpy library as: 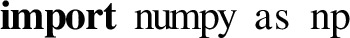 From now on, we shall call the nucleardatapy toolkit as nuda, and numpy as np in short.

To check that the toolkit is installed correctly and is accessible, enter into python and write at the prompt: 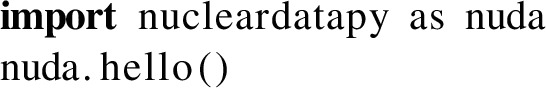 You should get hello world! in return for your hug!

The list of all functions and global variables available in the toolkit can be printed using: 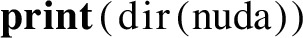 You can also have a detailed view of the routines with: 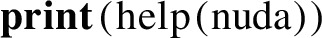 or 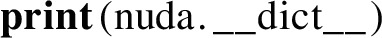 as well as 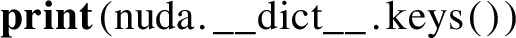


In the following, we detail the functions available for use in the nuda toolkit and discuss the results.

The nuda toolkit is divided into several modules: astro, corr, crust, eos, fig, hnuc, matter, and nuc. In addition, there are a few common files where global definitions are fixed: cst.py for fixing the constants, env.py for fixing environment flags (such as verb), param.py for provided parameters, for instance, the absolute paths to data files.

As an example, one can instantiate the variable mass with the neutron star mass measured by radio-astronomy in the following way: 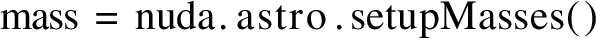 where the default pulsar is PSR J1614–2230. All the properties available for the variable mass are accessible via: 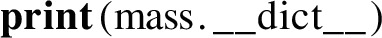 More details about the nuda.astro class nuda.astro. setupMasses() are given in Sect. 9.1.

The public GitHub repository [1] contains installation instructions as well as links to the documentation [2] and tutorials [3]. Detailed tutorials explaining the usage of the nuda toolkit are given in the documentation. Tutorials are distributed in several chapters, each dedicated to providing examples of usage for a given module. For instance, a tutorial providing massive neutron star masses can be found in chapter 8: Astrophysical Data. In addition, the figures shown in this paper can be reproduced using the tutorial and are available from the toolkit repository in the folder nucleardatapy_sample. The present paper provides details on how to employ the toolkit, for which it provides an introduction, while further details are given in the documentation [2] and tutorials [3].

We would also like to remind the users of the nuda toolkit to systematically provide citations to the original reference of the employed data. In the toolkit, all data are provided with their original reference, so when using these data in a scientific paper, references to data should be provided explicitly. To facilitate the quoting of the original references, we also provide a set of .bib files in the folder biblio on the GitHub repository [1]. These files are the ones employed for this paper. They are sorted chronologically. One can also cite this toolkit referring to the present paper, in addition to citing the original publication and/or data.

## Theoretical predictions for the ground state of uniform nuclear matter: the matter module

Consider a system composed of neutrons and protons, whose rest mass contribution to the energy is additive:

1 $$\begin{aligned} E_{r.m.} \equiv N_n m_n c^2 + N_p m_p c^2 , \end{aligned}$$

where $N_n$ ($N_p$) is the number of neutrons (protons) and $m_n$ ($m_p$) is the neutron (proton) mass. Introduce the nucleon mass $m_N$ defined as $m_N=(m_n+m_p)/2$. The rest mass contribution to the energy per particle ($e_{r.m.}$) reads,

2 $$\begin{aligned} e_{r.m.}\equiv \frac{E_{r.m.}}{N_\textrm{nuc}} = x_n m_n c^2 + x_p m_p c^2 , \end{aligned}$$

where the baryon number is $N_\textrm{nuc}=N_n+N_p$, the neutron (proton) fraction is $x_n=N_n/N_\textrm{nuc}$ ($x_p=N_p/N_\textrm{nuc}$).

In uniform matter, neutron and proton densities are defined as $n_n=N_n/V$ and $n_p=N_p/V$, where *V* is a mesoscopic volume containing $N_\textrm{nuc}$ nucleons. The total nucleon density is $n_\textrm{nuc}=n_n+n_p$ and the isospin parameter $\delta$ is defined as $\delta =(n_n-n_p)/n_\textrm{nuc}$. We have,

3 $$\begin{aligned} \delta = {\left\{ \begin{array}{ll} 0 & \quad \text {symmetric matter (SM)}, \\ 1 & \quad \text {neutron matter (NM)}.\\ \end{array}\right. } \end{aligned}$$

The total energy $E_\textrm{tot}$ is composed of the rest mass energy $E_{\mathrm {r.m.}}$ and the internal energy $E_\textrm{int}$ as,

4 $$\begin{aligned} E_\textrm{tot}\equiv E_{\mathrm {r.m.}} + E_\textrm{int}. \end{aligned}$$

Equation (4) is fixed by the requirement that non-relativistic approaches match relativistic ones at low momentum.

In the following, we consider predictions for the nucleon ground state, i.e., at zero temperature.

### Non-relativistic nucleonic free Fermi gas

The nucleonic free Fermi gas (FFG) is a uniform nuclear quantum system composed of fermions in their ground state with no interactions. In this section, we consider only non-relativistic (NR) FFG systems, for which the NR single-particle internal energy $e_q^{\textrm{NRFFG}}(k)$ is ($q=n$, *p*),

5 $$\begin{aligned} e_q^{\textrm{NRFFG}}(k) = \frac{\hbar ^2 k^2}{2m_q} . \end{aligned}$$

The Fermi energy is defined as the internal energy of the last occupied state for $k=k_{F_q}$,

6 $$\begin{aligned} e_{F_q}^{\textrm{NRFFG}} \equiv \frac{\hbar ^2 k_{F_q}^2}{2m_q} \equiv \mu _q -m_q c^2 , \end{aligned}$$

where $\mu _q$ is the ground state chemical potential. The internal energy per nucleon is obtained by adding the neutron and proton contributions as,

7 $$\begin{aligned} e_{\textrm{int}}^{\textrm{NRFFG}}(k_{Fn},k_{Fp}) \equiv \frac{3}{5}\left( \frac{\hbar ^2 k_{Fn}^2}{2m_n} + \frac{\hbar ^2 k_{Fp}^2}{2m_p} \right) , \end{aligned}$$

where the Fermi momentum is $k_{F_q}=(3\pi ^2 n_q)^{1/3}$, assuming spin-saturated systems (equal number of spin up and down). The nucleonic Fermi momentum is defined as $k_{F_\textrm{nuc}}=(3\pi ^2 n_\textrm{nuc}/2)^{1/3}$, and it corresponds to the momentum of the last occupied state in SM. Note that in SM, we have $k_{F_n}=k_{F_p}=k_{F_\textrm{nuc}}$. Introducing the density $n_\textrm{nuc}$ and the isospin asymmetry $\delta$ in Eq. (7), we obtain:

8 $$\begin{aligned} e_{\textrm{int}}^{\textrm{NRFFG}} = e_{\textrm{int},\textrm{sat}}^{\textrm{FFG}} \left( \frac{n_\textrm{nuc}}{n_\textrm{sat}}\right) ^{2/3}\frac{(1+\delta )^{5/3} + (1-\delta )^{5/3}}{2} , \end{aligned}$$

where the energy per particle at saturation density in SM is

9 $$\begin{aligned} e_{\textrm{int},\textrm{sat}}^{\textrm{FFG}} \equiv \frac{3}{5}\frac{\hbar ^2}{2m_N} \left( \frac{3\pi ^2}{2}n_\textrm{sat}\right) ^{2/3} \approx 22\hbox { MeV} , \end{aligned}$$

for $n_\textrm{sat}\approx 0.16$ fm$^{-3}$, $m_N c^2\approx 939$ MeV and $\hbar c\approx 197$ MeV fm. Note that Eq. (8) is the kinetic energy where the neutron and proton masses are fixed to be identical ($m_n\approx m_p\approx m_N$). This approximation is accurate and simplifies equations, but it is not necessary. The internal energy density reads,

10 $$\begin{aligned} \epsilon _{\textrm{int}}^{\textrm{NRFFG}}(n_\textrm{nuc},\delta ) = n_\textrm{nuc}\, e_{\textrm{int}}^{\textrm{NRFFG}}(n_\textrm{nuc},\delta ) , \end{aligned}$$

the total energy per nucleon is

11 $$\begin{aligned} e(n_\textrm{nuc},\delta ) = e_{r.m.}(n_\textrm{nuc},\delta )+e_{\textrm{int}}(n_\textrm{nuc},\delta ) , \end{aligned}$$

and the energy-density $\epsilon =e\, n_\textrm{nuc}$ is defined as

12 $$\begin{aligned} \epsilon (n_\textrm{nuc},\delta ) = \epsilon _{r.m.}(n_\textrm{nuc},\delta )+\epsilon _{\textrm{int}}(n_\textrm{nuc},\delta ) . \end{aligned}$$

Note that the energy density $\epsilon$ and the mass density $\rho$ are related in the following way:

13 $$\begin{aligned} \epsilon (n_\textrm{nuc},\delta ) = \rho (n_\textrm{nuc},\delta ) c^2 , \end{aligned}$$

where *c* is the speed of light.

The symmetry energy is defined as the difference between NM and SM,

14 $$\begin{aligned} e_\textrm{sym}(n_\textrm{nuc}) \equiv e_{\textrm{int}}(n_\textrm{nuc},\delta =1) - e_{\textrm{int}}(n_\textrm{nuc},\delta =0) . \end{aligned}$$

This definition applies to the NR FFG,

15 $$\begin{aligned} e_\textrm{sym}^{\textrm{NRFFG}}(n_\textrm{nuc}) = E_{\textrm{int},\textrm{sat}}^{\textrm{FFG}} \left( \frac{n_\textrm{nuc}}{n_\textrm{sat}}\right) ^{2/3}\left( 2^{2/3}-1\right) . \end{aligned}$$

The symmetry energy can also be expressed as a series expansion, as

$$\begin{aligned} e_\textrm{sym}(n_\textrm{nuc})=e_{\textrm{sym},2}(n_\textrm{nuc})+e_{\textrm{sym},4}(n_\textrm{nuc})+\cdots , \end{aligned}$$

where the quadratic and quartic contributions are defined as

16 $$\begin{aligned} e_{\textrm{sym},2}(n_\textrm{nuc})= &   \frac{1}{2} \frac{\partial ^2 e(n_\textrm{nuc},\delta )}{\partial \delta ^2} , \, \end{aligned}$$

17 $$\begin{aligned} e_{\textrm{sym},4}(n_\textrm{nuc})= &   \frac{1}{24} \frac{\partial ^4 e(n_\textrm{nuc},\delta )}{\partial \delta ^4} . \end{aligned}$$

For the NR FFG, we have

18 $$\begin{aligned} e_{\textrm{sym},2}^{\textrm{NRFFG}}(n_\textrm{nuc})= &   \frac{10}{18} E_{\textrm{int},\textrm{sat}}^{\textrm{FFG}} \left( \frac{n_\textrm{nuc}}{n_\textrm{sat}}\right) ^{2/3} , \, \end{aligned}$$

19 $$\begin{aligned} e_{\textrm{sym},4}^{\textrm{NRFFG}}(n_\textrm{nuc})= &   \frac{5}{243} E_{\textrm{int},\textrm{sat}}^{\textrm{FFG}} \left( \frac{n_\textrm{nuc}}{n_\textrm{sat}}\right) ^{2/3} . \end{aligned}$$

The nucleon pressure is defined as

20 $$\begin{aligned} p^{\textrm{NRFFG}}(n_\textrm{nuc},\delta )\equiv n_\textrm{nuc}^2\, \frac{\partial e^{\textrm{NRFFG}}}{\partial n_\textrm{nuc}} = \frac{2}{3} \epsilon _{\textrm{int}}^{\textrm{NRFFG}} , \end{aligned}$$

which means that the pressure does not depend on the rest mass energy of the particles (in this case). It can also be calculated using

21 $$\begin{aligned} p^{\textrm{NRFFG}}(n_\textrm{nuc},\delta )=\frac{n_\textrm{nuc}\, k_{F_\textrm{nuc}}}{3} \frac{\partial e^{\textrm{NRFFG}}}{\partial k_{F_\textrm{nuc}}} . \end{aligned}$$

The enthalpy per nucleon is defined as

22 $$\begin{aligned} h(n_\textrm{nuc},\delta ) \equiv \frac{d \rho (n_\textrm{nuc},\delta ) c^2}{d n_\textrm{nuc}} = e(n_\textrm{nuc},\delta ) + \frac{p(n_\textrm{nuc},\delta )}{n_\textrm{nuc}} , \end{aligned}$$

which is dominated, as lowest density, by the rest mass energy.

The derivative of the pressure is

23 $$\begin{aligned} \frac{\partial p^{\textrm{NRFFG}}}{\partial n_\textrm{nuc}} = \frac{10}{9} e^{\textrm{NRFFG}}_\textrm{int}(n_\textrm{nuc},\delta ) . \end{aligned}$$

The sound speed $c_s$ is defined as:

24 $$\begin{aligned} \left( c_{s}(n_\textrm{nuc},\delta )/c\right) ^2 \equiv \frac{\partial p}{\partial \epsilon } = \frac{1}{h(n_\textrm{nuc},\delta )} \frac{\partial p}{\partial n_\textrm{nuc}} , \end{aligned}$$

for fixed isospin asymmetry $\delta$. Note that at low density, when the rest mass energy dominates the total energy, we have

25 $$\begin{aligned} (c_s/c)^2 \xrightarrow [n \rightarrow 0]{} \frac{10}{9} \frac{e_\textrm{int}}{e_\mathrm {r.m.}} \propto n_\textrm{nuc}^{2/3} . \end{aligned}$$

**Table 1:** Functions from nucleardatapy toolkit associated with free Fermi gas quantities. The physics property is given in the first row, the corresponding method is in the second row, and finally the third row provides the associated attribute of the object ffg instantiated via the class matter.setupFFGNuc. The input variables can be scalar or numpy float array. We have: den_nuc for $n_\textrm{nuc}$ and delta for $\delta$. Note that both non-relativistic ($\textrm{NR}$) expressions and relativistic ones are provided

	Methods	Instantiation of ffg
		ffg=nuda.matter. setupFFGNuc
$k_{F_\textrm{nuc}}$	kf_nuc (den)	ffg.kf_nuc
$k_{F_n}$	kf_n (den_n)	ffg.kf_n
$k_{F_p}$	–	ffg.kf_p
$n_\textrm{nuc}$	den(kf)	ffg.den
$n_n$	den_n(kf_n)	ffg.den_n
$n_p$	–	ffg.den_p
$e_{F_n}$	eF_n(kf_n)	ffg.eF_n
$e_{F_n}^\textrm{NR}$	eF_n_nr(kf_n)	ffg.eF_n_nr
$e_{F_p}$	–	ffg.eF_p
$e_{F_p}^\textrm{NR}$	–	ffg.eF_p_nr
$e_\textrm{int}^{\textrm{NRFFG}}$	effg_nr(kf_n)	ffg.e2a_int_nr
$\epsilon ^{FFG}$	–	ffg.eps
$\epsilon _\textrm{int}^{FFG}$	–	ffg.eps_int
$e_\textrm{sym}^{\textrm{NRFFG}}$	–	ffg.esym
$e_{\textrm{sym},2}^{\textrm{NRFFG}}$	–	ffg.esym2
$e_{\textrm{sym},4}^{\textrm{NRFFG}}$	–	ffg.esym4
$p^{\textrm{NRFFG}}$	–	ffg.pre_nr
$p^{\textrm{FFG}}$	–	ffg.pre
$(c_s/c)^2$	–	ffg.cs2

These quantities are given in nuda toolkit, and the correspondence between these quantities and the nuda toolkit functions is given in Table 1. For instance, to obtain the FFG energies for a set of kf_n, one shall write the following lines in Python: 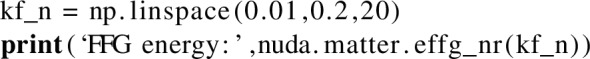 Alternatively, one can use the matter.setupFFGNuc class in the following way (for SM): 
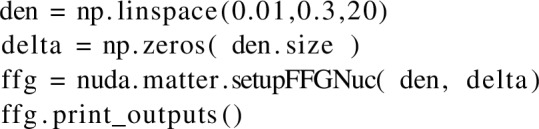


This option, in fact, provides more than just the energy. Most common attributes are the following ones: ffg.den_nuc for $n_\textrm{nuc}$, ffg.e2a_nuc for $e^{\textrm{FFG}}$, ffg.e2a_nuc_int for $e^{\textrm{FFG}}_\textrm{int}$, ffg.eps_nuc for $\epsilon ^{\textrm{FFG}}$, ffg.pre_nuc for $p^\textrm{FFG}$, ffg.cs2_nuc for $(c_s^\textrm{FFG}/c)^2$... For more attributes, see Table 1, while the full list of all attributes associated with the object ffg can be obtained via: 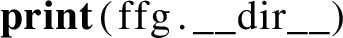

**Fig. 1:**
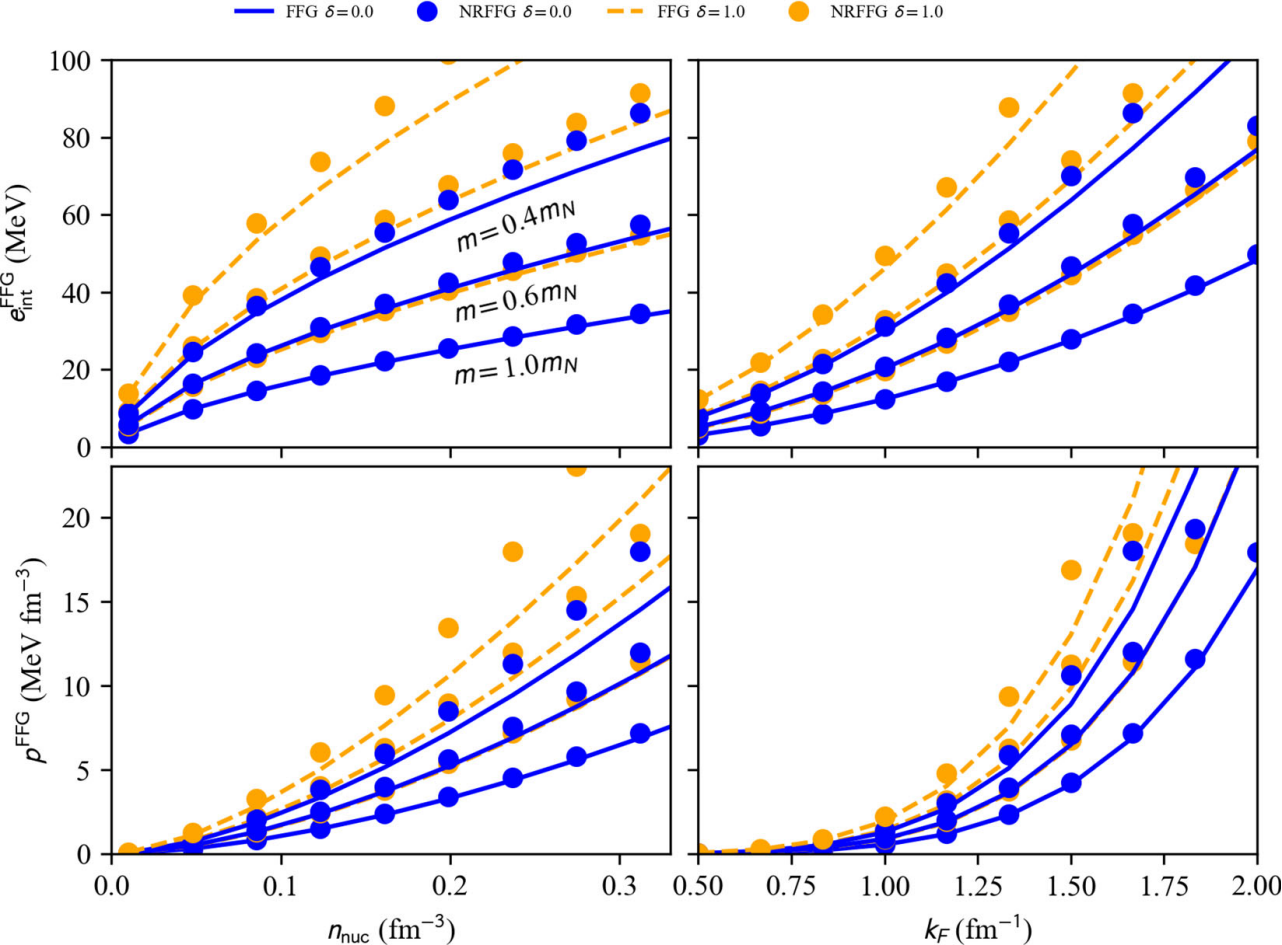
FFG energy in NM ($\delta =1$) and in SM ($\delta =0$) (top) and FFG pressure in NM and SM (bottom) as a function of the density $n_\textrm{nuc}$ (left) and the Fermi momentum $k_{F}$ (right). Lines (Symbols) show the relativistic (non-relativistic) FFG results. The nucleon mass is fixed to three constant values: $m_N$, $0.4m_N$, and $0.6m_N$. This figure is generated with matter_setupFFGNuc_plot.py

### Relativistic nucleonic free Fermi gas

The relativistic expression for the free Fermi gas energy density is

26 $$\begin{aligned} \epsilon ^{\textrm{RFFG}}(n) = C\Bigg [ 2 p_F E_F^3 - m^2 p_F E_F -m^4\log \frac{p_F+E_F}{m} \Bigg ] , \end{aligned}$$

where *n* is the particle density, $E_F=\sqrt{m^2+p_F^2}$, $p_F=\hbar c \, k_F$ the Fermi impulsion, $C=g/(16\pi ^2)$, *g* the degeneracy ($g=4$ in SM). The non-relativistic limit is recovered for low momenta ($p_F$, $k_F \rightarrow 0$), or equivalently low density, as

27 $$\begin{aligned} \epsilon ^{\textrm{RFFG}}(n) \xrightarrow [n \rightarrow 0]{} \left( m c^2+ \frac{3}{10} \frac{p_F^2}{m c^2} \right) \, n , \end{aligned}$$

and the ultra-relativistic limit ($p_F\gg mc^2$) gives:

28 $$\begin{aligned} e^{\textrm{URFFG}} = p_F = \hbar c \left( \frac{6 \pi ^2}{g} n\right) ^{1/3} . \end{aligned}$$

The pressure is

29 $$\begin{aligned} p^{\textrm{RFFG}}(n) = C^\prime \Bigg [ 2p_F E_F^3 - 5m^2 p_F E_F +3m^4\log \frac{p_F+E_F}{m} \Bigg ] , \end{aligned}$$

where $C^\prime =C/3$, and for the ultra-relativistic limit:

30 $$\begin{aligned} p^{\textrm{URFFG}}=\frac{1}{3} \epsilon ^{\textrm{URFFG}} . \end{aligned}$$

The enthalpy per particle is

31 $$\begin{aligned} h^{\textrm{RFFG}}(n) = \frac{8C}{3 n}\Bigg [ p_F E_F^3 - m^2 p_F E_F \Bigg ] . \end{aligned}$$

The derivative of the pressure is

32 $$\begin{aligned} \frac{\partial p^{\textrm{RFFG}}}{\partial n} = \frac{1}{3} \frac{p_F^2}{E_F} . \end{aligned}$$

The sound speed $c_s$ is:

33 $$\begin{aligned} \left( c_{s}(n)/c\right) ^2 = \frac{1}{h(n)} \frac{\partial p}{\partial n} , \end{aligned}$$

and for the ultra-relativistic limit,

34 $$\begin{aligned} (c_s^{\textrm{URFFG}}/c)^2 = 1/3 , \end{aligned}$$

which is also called the conformal limit for the sound speed.

For nuclear matter, we have

35 $$\begin{aligned} e_\textrm{nuc}=\frac{E_\textrm{nuc}}{A}=x_n \frac{E_n}{N} + x_p \frac{E_p}{Z}=x_n \frac{\epsilon _n(n_n)}{n_n} + x_p \frac{\epsilon _p(n_p)}{n_p} , \end{aligned}$$

where $x_i=n_i/n_\textrm{nuc}$ ($i=n$, *p*), $n_\textrm{nuc}=n_n+n_p$ and the energy densities $\epsilon _q$ are obtained from Eq. (26).

The object ffg can be instantiated to the class matter.setupFFGNuc in the following way (for SM in this example): 
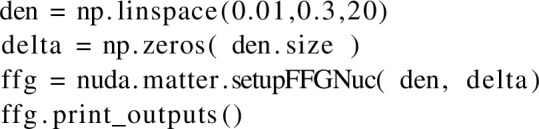


The FFG energy and pressure in SM (solid blue) and NM (dashed yellow) are shown in Fig. 1 as a function of the density $n_\textrm{nuc}$ (left) and Fermi momentum $k_{F}$ (right). A comparison of the non-relativistic results (circles) with the relativistic ones (lines) is shown. We note that in the case of nucleons, the relativistic corrections to the FFG are small for densities below $2n_\textrm{sat}$ and for $m=m_N$. The impact of the relativistic corrections increases as the nucleon mass *m* decreases. We show in Fig. 1 situations where the mass *m* is reduced to $0.6m_N$ and $0.4m_N$, which correspond to the current expectation for the effective mass at $\approx n_\textrm{sat}$ and $\approx 2n_\textrm{sat}$. Note, however, that the effect of the density dependence of the effective mass (rearrangement contribution) is not considered here. As the mass *m* is reduced, the difference between the non-relativistic and the relativistic quantities is increased, and these differences are larger in NM compared to SM.

**Fig. 2:**
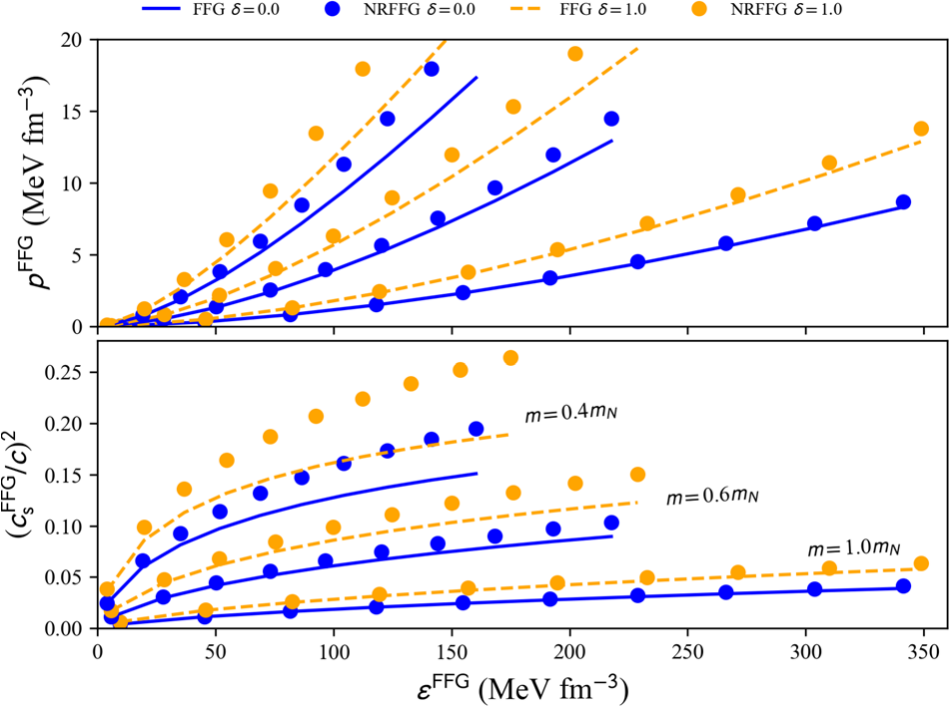
FFG EoS: pressure *p* (top) and sound speed $(c_s/c)^2$ (bottom) for SM (solid blue lines) and NM (dashed yellow lines) function of the energy density $\epsilon$. Circles (lines) show the non-relativistic (relativistic) FFG results. This figure is generated with matter_setupFFGNuc_plot.py

The EoS is shown in Fig. 2, with the pressure *p* function of the energy density $\rho c^2$ (top panel) and the sound speed squared $(c_s/c)^2$ function of the energy density $\epsilon$. Similarly to Fig. 1, we show relativistic and non-relativistic quantities, in SM and NM, and for different choices for the nucleon mass. We observe that the sound speed increases as the mass *m* decreases: it reaches 0.07 (0.15) for $0.6m_N$ ($0.4m_N$) at saturation density.

### Relativistic free Fermi gas for leptons

Electrons and muons are contributing to the leptonic energy in neutron stars. The mass of the $\tau$ is too high to contribute in neutron stars. We have

36 $$\begin{aligned} \epsilon _\textrm{lep}= \frac{E_\textrm{e}+E_\mu }{V} = \epsilon _\textrm{e}+ \epsilon _\mu , \end{aligned}$$

and

37 $$\begin{aligned} e_\textrm{lep}=\frac{E_\textrm{lep}}{N_\textrm{lep}}=\tilde{x}_\textrm{e}\frac{E_\textrm{e}}{N_\textrm{e}} + \tilde{x}_\mu \frac{E_\mu }{N_\mu }=\tilde{x}_\textrm{e}\frac{\epsilon _\textrm{e}}{n_\textrm{e}} + \tilde{x}_\mu \frac{\epsilon _\mu }{n_\mu } , \end{aligned}$$

where $N_\textrm{lep}=N_\textrm{e}+N_\mu$, $\tilde{x}_i=N_i/N_\textrm{lep}=n_i/n_\textrm{lep}$ ($i=\textrm{e}$, $\mu$). Electrons and muons are present in the core of cold neutron stars (in fact, muons are present for densities $n\gtrapprox n_\textrm{sat}$). In the following example, the terms $\epsilon _\textrm{e}$ and $\epsilon _\mu$ are properties of the object lep instantiated from the class nuda.matter.setupFFGLep in the following way: 
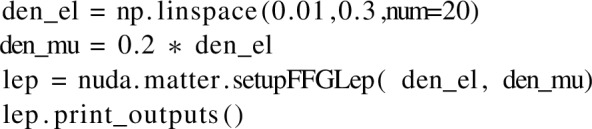
 where den_el and den_mu are the number densities (in fm$^{-3}$) associated with the electrons and the muons. The electron (muon) energy density $\epsilon _\textrm{e}$ ($\epsilon _\mu$) is given as an attribute of the object lep as lep.eps_el (lep.eps_mu).

**Fig. 3:**
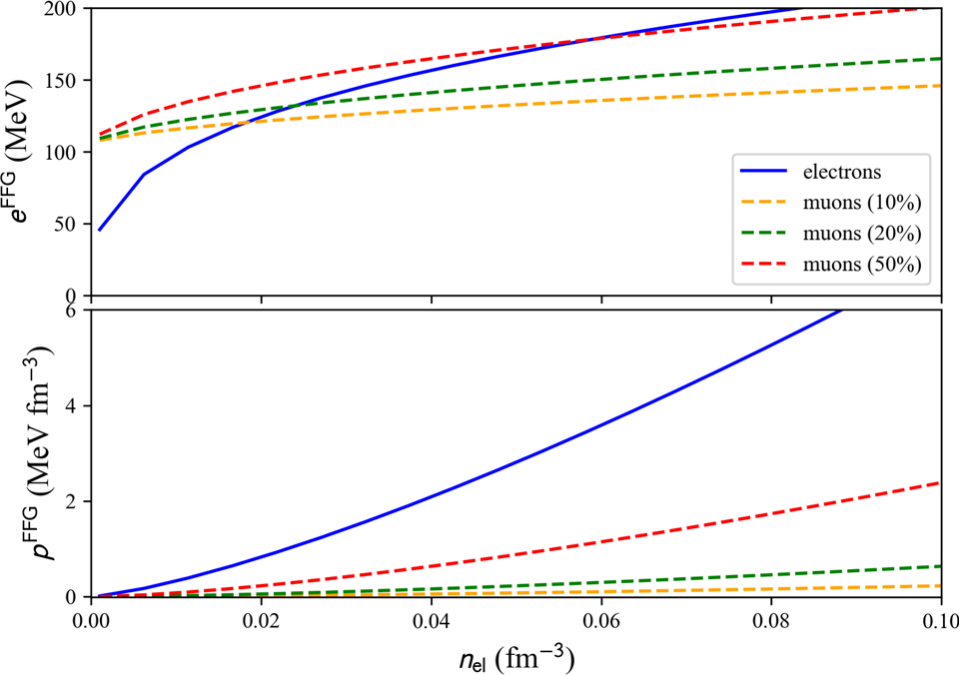
Leptonic FFG: energy per particle for electrons (solid) and muons (dashed) (top) and pressure (bottom). We have considered three scenarios for muons: 10% of the electron density (yellow), 20% (green) and 50% (red). This figure is generated with matter_setupFFGLep_plot.py

The relativistic leptonic FFG is shown in Fig. 3. Solid lines stand for the electron contribution while dashed lines for the muon one for $n_\mu =0.1 n_\text {el}$ (yellow), $n_\mu =0.2 n_\text {el}$ (green), $n_\mu =0.5 n_\text {el}$ (red). At low density, the rest mass contribution to the energy dominate, while the rest mass contribution decreases as the density increases. This effect is more pronounced for the electrons and less for the muons. For the pressure, there is no rest mass contribution and the electron pressure is always larger than the muon pressure.

### Microscopic models

In this section, we list the microscopic models available in the nuda toolkit. These microscopic predictions are grouped into several many-body approaches. The following instruction provides the list of many-body groups: 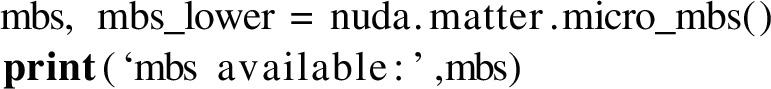 It provides the following list: [ ‘VAR’, ‘AFDMC’, ‘BHF2’, ‘BHF23’, ‘QMC’, ‘MBPT’, ‘NLEFT’ ], which can be completed if necessary. The following instruction provides a list of models inside a given group defined by the variable mb in the class nuda.matter.micro_models_mb():
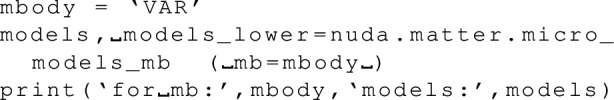


Instead of a single many-body group, a list of groups can be provided to obtain the associated list of model predictions, as in this example:
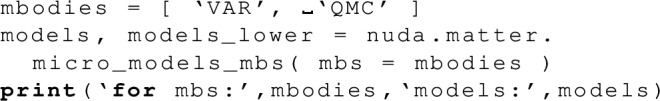


The complete list of available microscopic models is given with the following instructions: 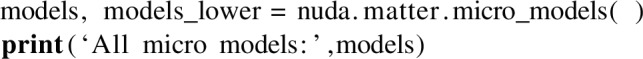


The class matter.setupMicro(model) provides the following attributes: nm_e2a, sm_e2a (energy per particle in SM and NM), nm_e2a_int, sm_e2a_int (internal energy per particle in SM and NM), nm_eps, sm_eps (energy density in SM and NM) and the pressure nm_pre and sm_pre, related to the cubic-spline derivative of the energy per particle. The chemical potentials are obtained, similarly, from a cubic-spline of the energy per nucleon and are encoded in the properties: sm_chempot, nm_chempot. If provided by the authors, the pairing gap nm_gap and sm_gap are also given by the toolkit, otherwise, these properties are defined as None.

The call for the results of a microscopic calculation for a given model, here the FP EoS, can be done in the following way:
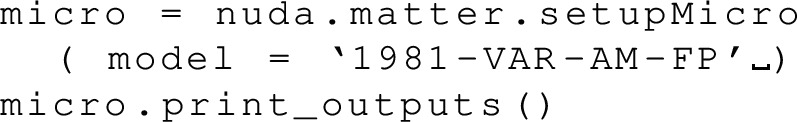


In the following, the variable model will be varied on all the EoS available in the nuda toolkit. We adopt the following convention model=‘YYYY-X-Y-Z’, where:

- ‘YYYY’ stands for the year associated with the publication of the results;
- ‘X’ for the many-body technique, e.g., VAR, AFDMC, QMC, etc.;
- ‘Y’ is ‘NM’ for neutron matter only, and ‘AM’ for SM and NM;
- ‘Z’ is optional: it is the name of the EoS when it exists, e.g., FP, APR, or of the nuclear interaction, e.g., Av18.

For this example, output quantities are attributes of the object micro, for instance: micro.nm_e2a (micro.nm_e2a_int) and micro.sm_e2a (micro.sm_e2a_int) arrays for the nucleon (internal) energy per particle in NM and SM with obvious notations.^2^

#### Variational approaches

In nuda toolkit, two variational EoS are provided: FP [21] and APR [22]. They are described below. Note that in the case of APR EoS, the authors of the original paper provide a fit in addition to their results. In nuda toolkit, we therefore provide their fit, based on continuous functions, which allows us to perform accurate first and second-order derivations.

A typical call for a given model, see hereafter, is: 
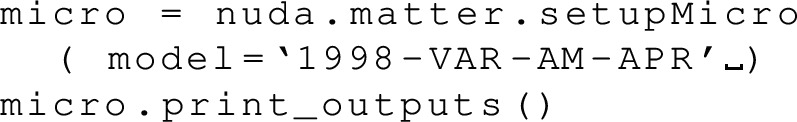
 where the object micro contains a large set of properties, some of them listed by the command micro.print_ outputs(). The entire list of properties are obtained in the following way: 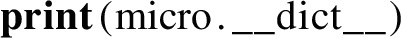


We now list the options available for the variable model. Note that the code will stop if the variable model is misspelled.

model=‘1981-VAR-AM-FP’:

The Friedman-Pandharipande (FP) EoS is obtained from a variational hypernetted-chain approach based on $v_{14}$ two-nucleon interaction and complemented by TNI three-nucleon interaction. The data provided by nuda are extracted from Ref. [21], more specifically from the second column of Table 1 for SM and Table 2 for NM.

**Table 2:** Parameter of the function (39) fitted on the NLEFT predictions for the energy per particle in SM and NM from Ref. [43] and considering $a=1$, to get the FFG limit as the density goes to zero

Parameters	*b*	*c*	*d*
Matter	(fm)	(fm$^2$)	(fm$^3$)
SM	$-\,9.171\pm 0.505$	$10.01\pm 1.40$	$-\,3.070\pm 0.239$
NM	$-\,1.188\pm 0.014$	$0.719\pm 0.022$	$-\,0.113\pm 0.002$

**Fig. 4:**
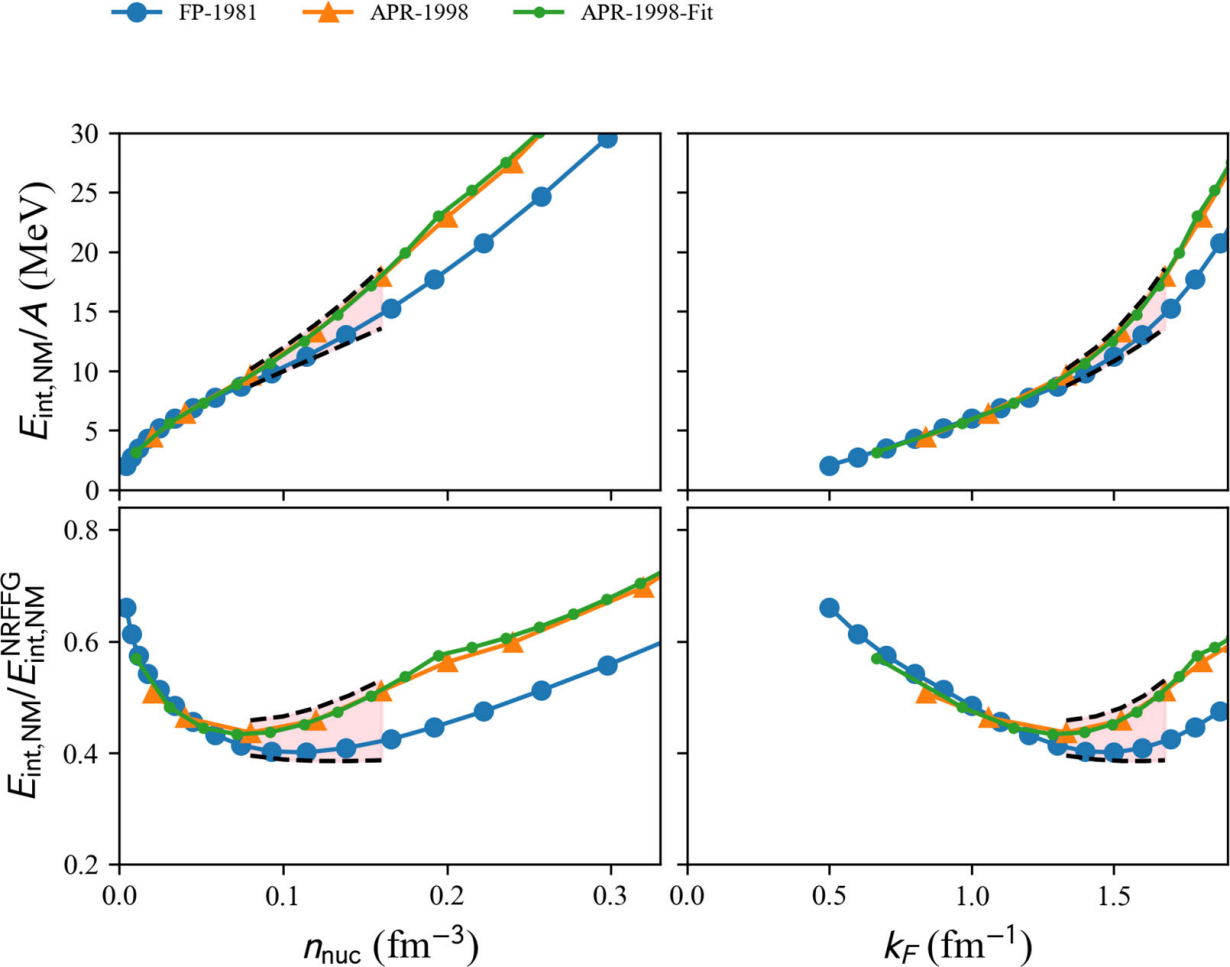
Internal energy per nucleon in neutron matter (NM) $E_{\textrm{NM}}^\textrm{int}$ (top) and $E_{\textrm{NM}}^\textrm{int}$ over the non-relativistic free Fermi gas energy (bottom) as a function of the density (left) and the Fermi momentum (right) for the variational models (FP and APR) available in nuda toolkit. The reference band detailed in Sect. 3.5 is also shown (pink area). We have applied here and in the next figures a selection of the models: all those passing through the reference band are shown in solid lines, the ones not passing through it in dashed lines. Since all models shown in this figure pass through the reference band, they are all shown with solid lines. Figure generated with matter_setupMicro_plot.py

model=‘1998-VAR-AM-APR’:

The Akmal-Pandharipande-Ravenhall (APR) EoS is obtained from a variational chain summation calculation for SM and NM based on Argonne $v_{18}$ two-nucleon interaction and complemented with three-nucleon interaction and boost corrections (leading order relativistic correction). The data provided by nuda are extracted from Ref. [22], more specifically from the last column (corrected) of Table 6 for SM and the last column of Table 7 for NM.

**Fig. 5:**
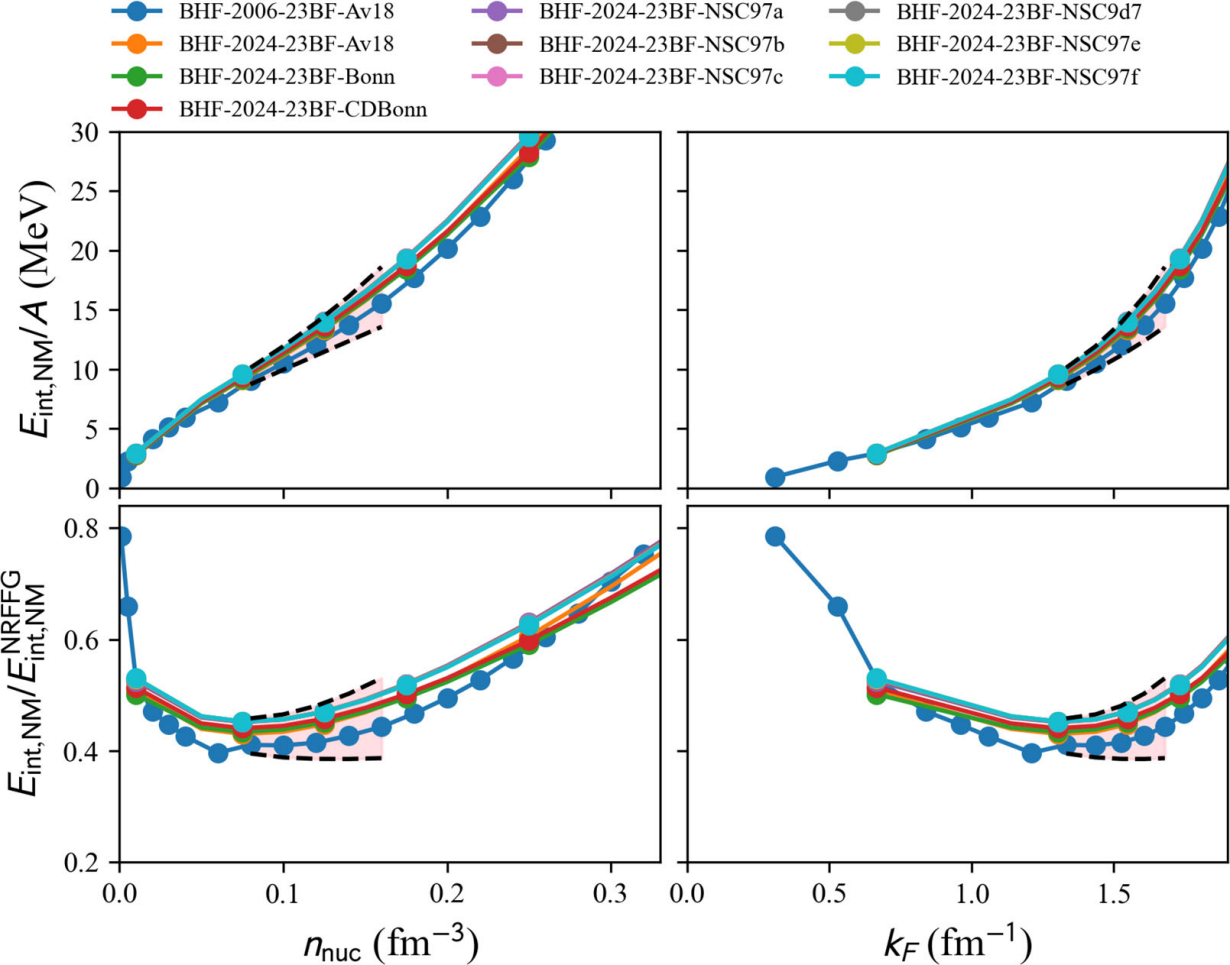
Same as Fig. 4 for BHF23 models with 2+3BF available in nuda toolkit. Figure generated with matter_setupMicro_plot.py

model=‘1998-VAR-AM-APR-fit’:

The suggested fit of the APR EoS in Ref. [22] is provided for a set of densities and isospin asymmetries given in the arrays var1 (densities) and var2 (isospin asymmetries). The call of the class nuda.matter.setupMicro() is slightly modified as:
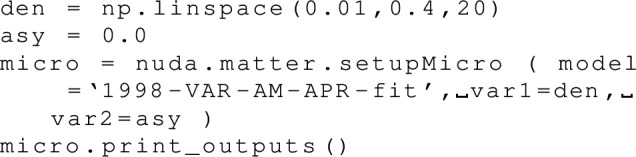


Note that var1 can be an array of density, while var2 is a scalar fixing the isospin asymmetry.

We show in Fig. 4 the variational models FP and APR. The symbols represent the data provided in the tables from Refs. [21, 22] while the solid line is the fit provided in Ref. [22]. The top panels show the total NM energy and the bottom panel shows the energy divided by the NRFFG, so $E_{\textrm{NM}}/E_{\textrm{NRFFG}}$ highlights the contribution of the potential energy given by the models. The band represents the reference uncertainties presented in Sect. 3.5. Interestingly, the FP and APR EoS are located inside the reference band, which is solely defined by different many-body approaches based on similar $\chi$EFT interactions [23, 24].

#### Brueckner–hartree–fock

There are several calculations based on the Brueckner–Hartree–Fock (BHF) approach and employing several two- and three-body interactions. In nuda toolkit, we provide a large number of calculations originating from a few papers. For instance, BHF calculations where a large set of nuclear interactions involving two- and three-nucleons have been performed in Ref. [25] for two-nucleon interactions only, for two- and three-nucleon phenomenological interactions, and for two- and three-nucleon microscopic interactions. The toolkit provides the results for all these calculations and also for other calculations.

A typical call for a given model, see hereafter, is: 
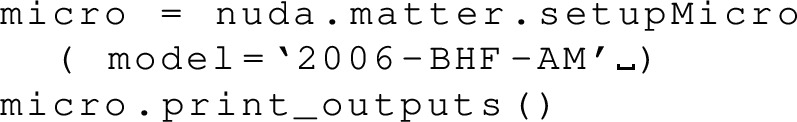
 Each calculation can be obtained by fixing the variable model to one of the following values:

model=‘2006-BHF-AM’:

Calculation based on an extended version of the Brueckner–Hartree–Fock (BHF) approach and presented in Ref. [26].

Only two-nucleon interaction:

- model=‘2024-BHF-AM-2BF-Av18’.
- model=‘2024-BHF-AM-2BF-BONN’.
- model=‘2024-BHF-AM-2BF-CDBONN’.
- model=‘2024-BHF-AM-2BF-NSC97a’.
- model=‘2024-BHF-AM-2BF-NSC97b’.
- model=‘2024-BHF-AM-2BF-NSC97c’.
- model=‘2024-BHF-AM-2BF-NSC97d’.
- model=‘2024-BHF-AM-2BF-NSC97e’.
- model=‘2024-BHF-AM-2BF-NSC97f’.

Two- and three-nucleon phenomenological interaction:

- model=‘2024-BHF-AM-23BF-Av18’.
- model=‘2024-BHF-AM-23BF-BONN’.
- model=‘2024-BHF-AM-23BF-CDBONN’.
- model=‘2024-BHF-AM-23BF-NSC97a’.
- model=‘2024-BHF-AM-23BF-NSC97b’.
- model=‘2024-BHF-AM-23BF-NSC97c’.
- model=‘2024-BHF-AM-23BF-NSC97d’.
- model=‘2024-BHF-AM-23BF-NSC97e’.
- model=‘2024-BHF-AM-23BF-NSC97f’.

Two- and three-nucleon microscopic interaction:

- model=‘2024-BHF-AM-23BFmicro-Av18’.
- model=‘2024-BHF-AM-23BFmicro-BONNB’.
- model=‘2024-BHF-AM-23BFmicro-NSC93’.

We show in Fig. 5 a set of BHF models with 2+3BF interactions. Calculations with only 2BF are provided in the toolkit, as detailed hereinbefore, but they are not interesting to show in Fig. 5 because they do not saturate. The band represents the reference uncertainties presented in Sect. 3.5. Again, it is interesting to note that BHF23 calculations are compatible with the reference band.

**Fig. 6:**
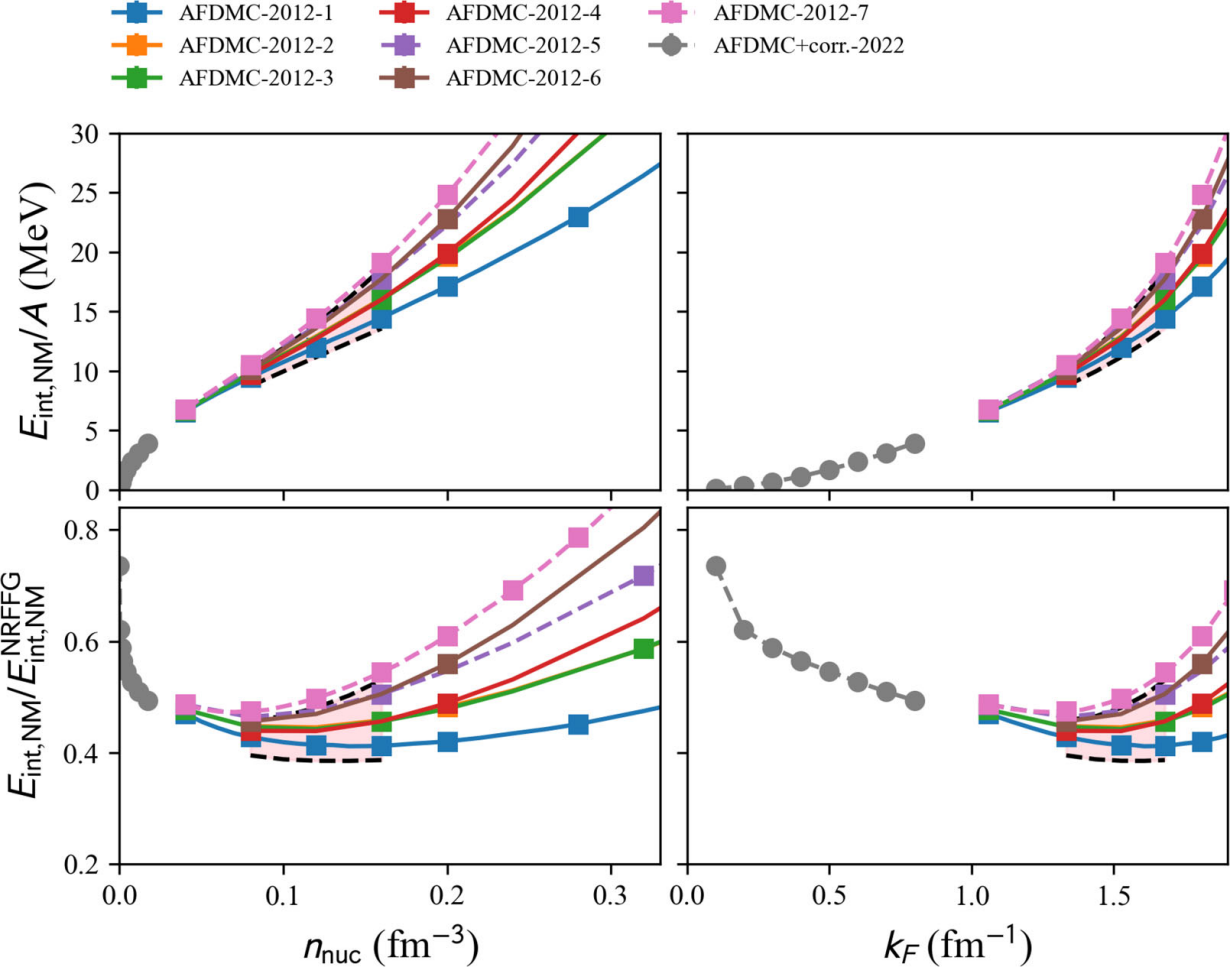
Same as Fig. 4 for AFDMC models with 3BF available in the nucleardatapy toolkit. Note that the models in dashed lines are the ones that do not pass through the reference band. Figure generated with matter_setupMicro_plot.py

#### Auxiliary field diffusion Monte-Carlo

A set of Auxiliary Field Diffusion Monte Carlo (AFDMC) is provided in the nuda toolkit. All these EoS are calculated in NM, even the fit provided in ‘2012-AFDMC-NM-FIT-n’.

A typical call for a given model, see hereafter, is: 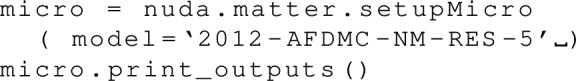 Each calculation can be obtained by fixing the variable model to one of the following values:

model=‘2012-AFDMC-NM-RES-n’ (with n=1,7):

AFDMC calculations with AV8$^\prime$ two-nucleon interaction and seven models for the three-nucleon interaction have been performed in Ref. [27]. The index *n* runs over the different three-nucleon interactions considered: none ($n=1$), $V_{2\pi }^{PW}+V_{\mu =150}^R$ ($n=2$), $V_{2\pi }^{PW}+V_{\mu =300}^R$ ($n=3$), $V_{3\pi }+V_R$ ($n=4$), $V_{2\pi }^{PW}+V_{\mu =150}^R$ ($n=5$), $V_{3\pi }+V_R$ ($n=6$), UIX ($n=7$).

model=‘2012-AFDMC-NM-FIT-n’ (with n=1,7):

Fit of AFDMC calculations with AV8$^\prime$ two-nucleon interaction and seven models for the three-nucleon interaction have been performed in Ref. [27].

model=‘2022-AFDMC-NM’:

The AFDMC approach is employed to calculate the binding energy and the $^1$S$_0$ pairing gap in NM using realistic nuclear Hamiltonians that include two- and three-body interactions in Ref. [28]. The trial state is properly optimized to capture the essential pairing correlations and the results are extrapolated from the finite box to the thermodynamic limit using the symmetry-restored projected Bardeen-Cooper-Schrieffer (PBCS) theory. The pairing gap shows a modest suppression with respect to the mean-field BCS values, see Fig. 14, except around the peak of the pairing gap for $k_F$ around 0.8 fm$^{-1}$.

We show in Fig. 6 a set of AFDMC models. The band represents the reference uncertainties presented in Sect. 3.5. Most of AFDMC calculation are located inside the reference band, except ‘AFDMC-2012-7’, which is more repulsive than the upper bound of the reference band and ‘AFDMC+corr.-2022’, which is only provided for densities below the densities of the reference band.

**Fig. 7:**
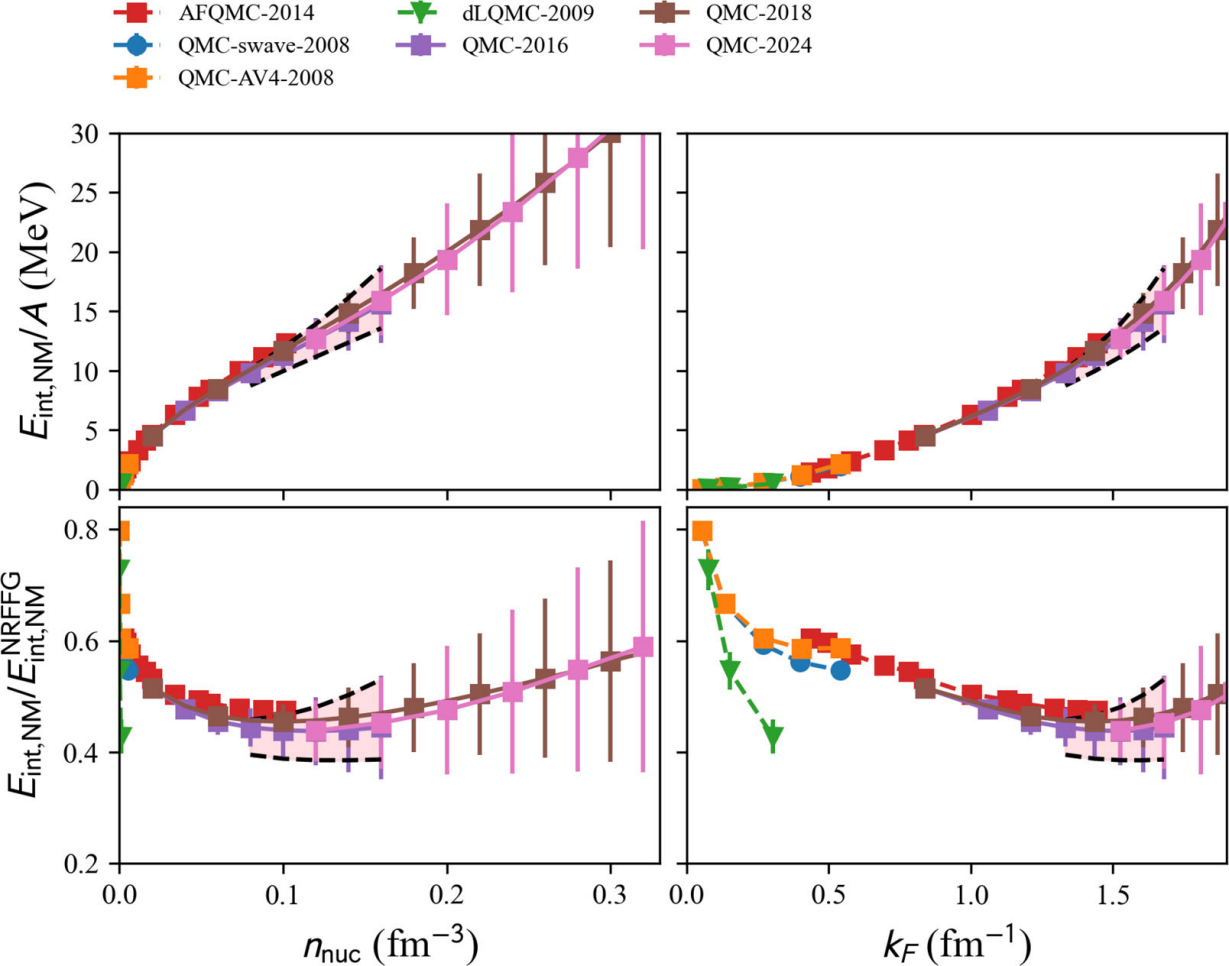
Same as Fig. 4 for QMC models available in the nucleardatapy toolkit. Figure generated with matter_setupMicro_plot.py

#### Quantum monte carlo

Several Quantum Monte Carlo (QMC) calculations based on phenomenological 2BF as well as on $\chi$EFT nuclear interactions are available from the nuda toolkit.

A typical call for a given model, see hereafter, is: 
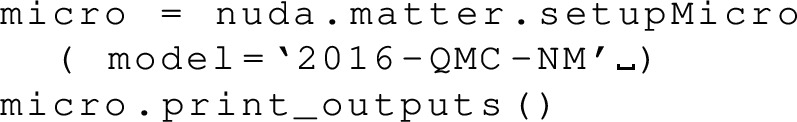
 Each calculation can be obtained by fixing the variable model to one of the following values:

model=‘2008-QMC-NM-swave’,

Quantum Monte-Carlo methods (variational and Green’s function) are employed to study dilute neutron matter using *s* wave terms of the AV18 interaction [29] in Ref. [30]. Calculations are performed for $k_{F_n}=0.05$ fm$^{-1}$ up to 0.55 fm$^{-1}$.

model=‘2009-DLQMC-NM’:

This is the determinantal lattice QMC performed in Ref. [31].

model=‘2010-QMC-NM-AV4’:

Quantum Monte-Carlo methods (variational and Green’s function) are employed to study dilute neutron matter using *s* and *p*-wave terms, where the *s*-wave term is the same as in Ref. [30] and the *p*-wave term is determined by the AV4 interaction [32] in Ref. [33]. Calculations are performed for $k_{F_n}=0.05$ fm$^{-1}$ up to 0.55 fm$^{-1}$.

model=‘2014-AFQMC-NM’:

NM variational Monte Carlo calculation using chiral nuclear forces at N3LO (N2LO) for the two-nucleon (three-nucleon) nuclear force in [34].

model=‘2016-QMC-NM’:

QMC calculations of NM with chiral three-body forces at N2LO are reported in [35]. Calculations are performed for densities $n_\textrm{nuc}=0.02$ fm$^{-3}$ up to 0.16 fm$^{-3}$.

model=‘2018-QMC-NM’:

QMC calculations of NM with N2LO chiral Hamiltonians, including 3N interactions with only the two-pion exchange, and 3N interactions containing the two-pion exchange plus shorter-range contact terms with two different spin-isospin operators. Calculations [23] are performed for densities $n_\textrm{nuc}=0.04$ fm$^{-3}$ up to 0.32 fm$^{-3}$.

model=‘2024-QMC-NM’:

Ref. [36] reported QMC calculations of NM with large-cutoff (400 MeV - 700 MeV) derived within N2LO chiral EFT, adjusted to nucleon-nucleon scattering phase shifts, the triton binding energy, as well as the triton beta-decay half-life. Calculations are performed for densities $n_\textrm{nuc}=0.08$ fm$^{-3}$ up to 0.2 fm$^{-3}$.

We show in Fig. 7 a set of QMC models. The band represents the reference uncertainties presented in Sect. 3.5. All the centroid predictions from QMC calculations are located inside the reference band, except ‘AFQMC-2014’, which is more repulsive than the upper boundary of the reference band, especially at low density.

**Fig. 8:**
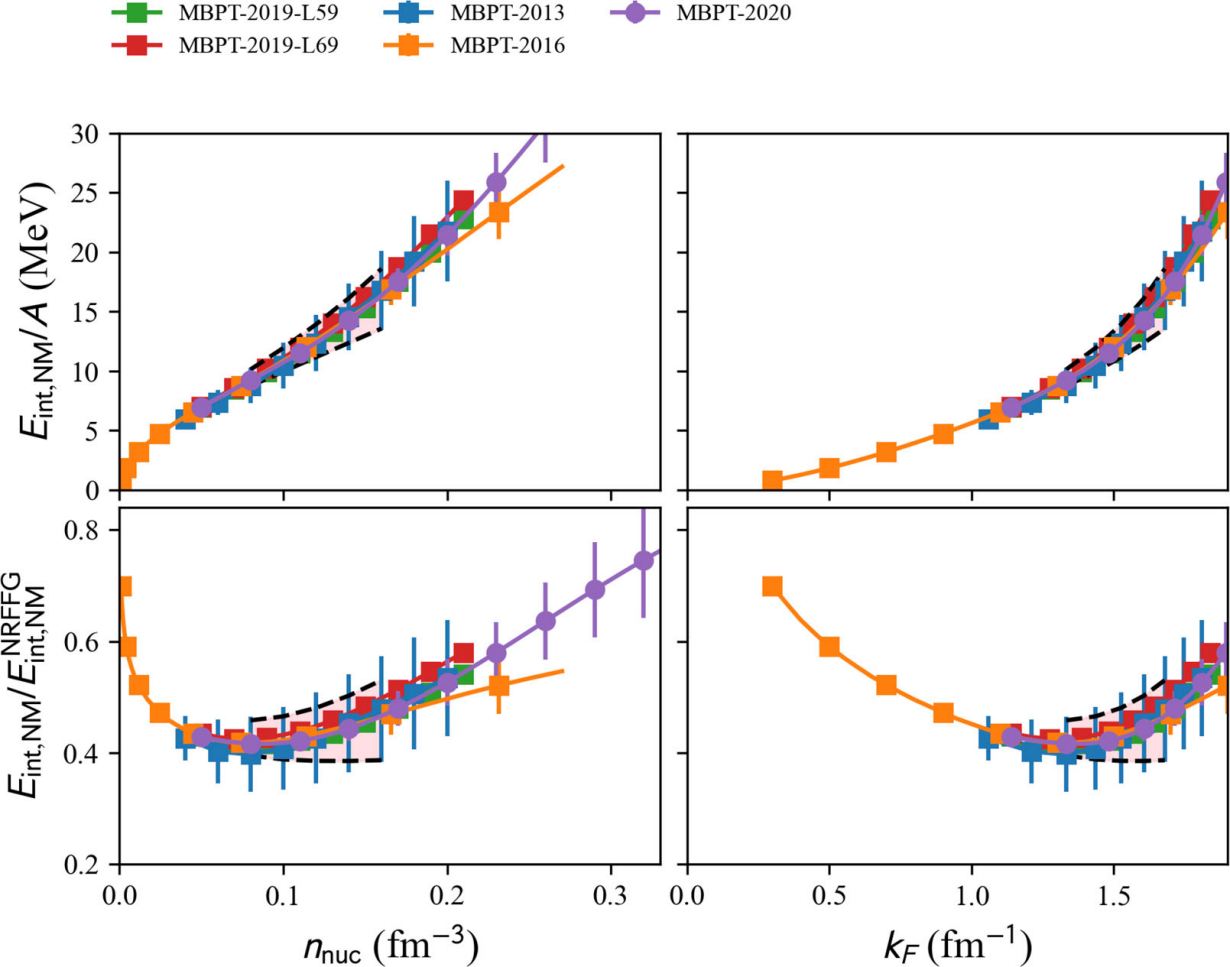
Same as Fig. 4 for MBPT models available in the nucleardatapy toolkit. Figure generated with matter_setupMicro_plot.py

#### Many-body perturbation theory

Several Many-Body Perturbation Theory (MBPT) calculations with N2LO and N3LO $\chi$EFT interactions are available in the nuda toolkit.

A typical call for a given model, see hereafter, is: 
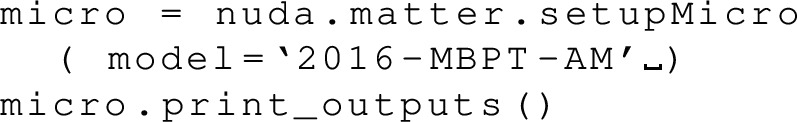
 Each calculation can be obtained by fixing the variable model to one of the following values:

model=‘2013-MBPT-NM’:

MBPT calculations based on the two-nucleon and three-nucleon interaction at N3LO (next-to-next-to-next-to-leading order) have been performed in NM in [37]. The three-nucleon interactions at N3LO were considered only at the Hartree–Fock level.

model=‘2016-MBPT-AM’:

Explicit calculations of isospin-asymmetric nuclear matter based on chiral two-nucleon interactions at N3LO and three-nucleon interactions at N2LO calculated in second-order MBPT are reported in Ref. [38].

Results for isospin asymmetric matter are also provided in Ref. [38]. They can be obtained from the nuda toolkit requesting the following properties: micro.am_e2a_int_av[i] and micro.am_e2a_int_err[i] for the average and uncertainty band for the internal energy in asymmetric matter, where $\delta =i/10$ and $i=0$, 10. The nucleon density in asymmetric matter can be obtained in the following way: micro.am_den[i]. The neutron and proton fractions can be obtained as: micro.am_xn[i] and micro.am_xp[i]. Detail results from each of the hamiltonians H$_j$, see Ref. [38] for more details, can be obtained from the following property: micro.am_e2a_int[i,j].

model=‘2019-MBPT-AM-DHSL59’, ‘2019-MBPT-AM-DHSL69’:

Reference [39] presented a Monte Carlo framework for higher-order MBPT calculations. The results are presented for SM and NM based on chiral two-, three-, and four-nucleon interactions at N2LO and N3LO, respectively. DHSL59 calculations represent $L_{\textrm{sym}} = 59$ MeV while DHSL69 has $L_{\textrm{sym}} = 69$ MeV. The calculations were performed for densities $n_\textrm{nuc}=0.05$ fm$^{-3}$ up to 0.21 fm$^{-3}$.

model=‘2020-MBPT-AM’:

References [40, 41] perform a statistical uncertainty quantification of NM based on the MBPT calculations in Ref. [39] using chiral EFT interactions up to N3LO. The model applies Bayesian machine learning with Gaussian processes to quantify correlated truncation errors due to truncating the $\chi$EFT at a finite order (so-called EFT truncation errors). Predictions for the energy per particle, pressure, and speed of sound of neutron matter up to twice nuclear saturation density, along with constraints on the nuclear symmetry energy and its derivative, are provided. The authors (i.e., the BUQEYE collaboration) made their source codes publicly available [42].

We show in Fig. 8 a set of MBPT calculations. The band represents the reference uncertainties presented in Sect. 3.5. Again, it is interesting that MBPT calculations are compatible with the reference band.

**Fig. 9:**
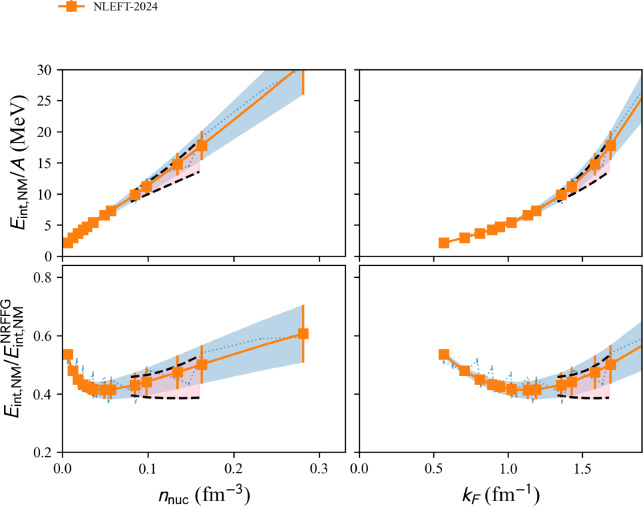
Same as Fig. 4 for NLEFT models available in the nucleardatapy toolkit. Figure generated with matter_setupMicro_plot.py

#### Nuclear lattice effective field theory

The complex problem of systems where the interactions may be complicated is addressed using wavefunction matching, which transforms the interaction between particles into an easily computable interaction. A recent calculation has been done in Ref. [43] for NM and SM using Nuclear Lattice Effective Field Theory (NLEFT). These calculations are performed in different box sizes, and the finite-size effects have to be removed. To do so, we adjust a function $f(k_F)$ which depends only on $k_F$ and which represents the difference between the energy per particle *E*/*A* and the non-relativistic Fermi gas prediction:

38 $$\begin{aligned} E_\text {NLEFT}(k_F) \approx f(k_F) \, E^{\textrm{NRFFG}}(k_F) , \end{aligned}$$

and we consider the following polynomial expansion for the function *f*,

39 $$\begin{aligned} f(k_F) = a + b k_F + c k_F^2 + d k_F^3 , \end{aligned}$$

where the parameters *a*, *b*, *c*, and *d* are obtained by fitting the NLEFT prediction for the energy per particle in SM and NM from Ref. [43], see Table 2. We impose $a=1.0$ to get the low-density limit given by the FFG.

A typical call for a given model, see hereafter, is: 
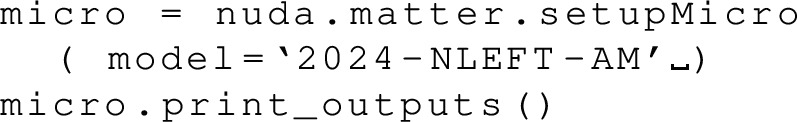
 The present toolkit provides results for only one NLEFT calculation. It is, however, ready for more results by specifying them in the variable model. Here is an example:

model=‘2024-NLEFT-AM’:

Consider the predictions for SM and NM from Ref. [43] and perform the fit defined by Eq. (38). The parameters of the function $f(k_F)$ defined in Eq. (39) are those given in Table 2 for SM and NM.

We show in Fig. 9 results from NLEFT given in Ref. [43]. The blue area represents the result of the fit to NLEFT calculations considering the uncertainties. The band represents the reference uncertainties presented in Sect. 3.5. Here also, there is a nice overlap between the NLEFT results and the reference band.

The bare data extracted from the tables given in Ref. [43] are stored in the properties micro.nm_e2a_int_data and micro.sm_e2a_int_data, they are shown in blue dotted line in Fig. 9, while the results of the fit are stored in micro.nm_e2a_int and micro.sm_e2a_int (with errors in micro.nm_e2a_err and micro.sm_e2a_err) and are shown in solid line in Fig. 9.

### Uncertainties in the model predictions

To compare different results from different models, it is necessary to know the uncertainties associated with these models. Since these uncertainties are not always estimated, we suggest a function that provides generic average uncertainties that could be associated to a given model. These uncertainties are extracted from recent QMC and MBPT approaches, where they are accurately estimated, as we detail hereafter.

There are different sources of uncertainties in the model prediction, which are addressed in different ways by different authors. The first source of uncertainties is related to the Hamiltonian itself, which is mainly due to the truncation of the operator basis of nuclear interactions and is partially connected to the statistical uncertainties in the fits to data. These uncertainties are estimated by some authors, especially in the most recent publications, see Figs. 7 and 8 for instance.

**Fig. 10:**
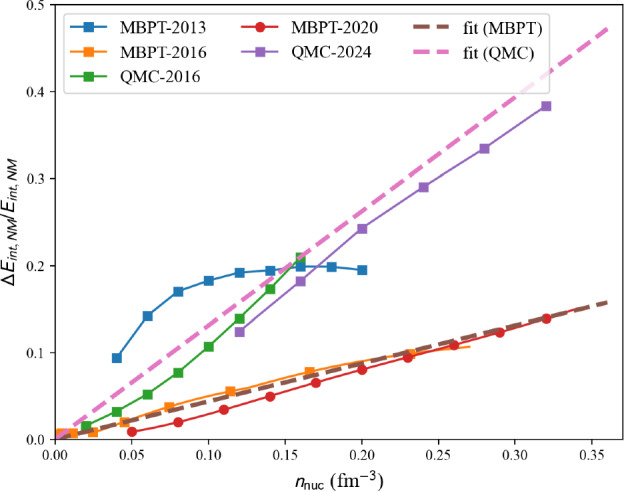
Relative uncertainties in NM estimated by different calculations: MBPT-2013 [37], MBPT-2016 [38], QMC-2016 [35], MBPT-2020 [39, 40], QMC-2024 [36]. The dashed lines represent fits to MBPT, except MBPT-2013, and QMC results, see the text for more details. Figure generated with matter_setupMicroErr_plot.py

We show in Fig. 10, the relative uncertainties in NM, $\varDelta E_{NM}/E_{NM}$, as a function of the density for various calculations: MBPT-2013 [37], MBPT-2016 [38], QMC-2016 [35], MBPT-2020 [39, 40], QMC-2024 [36]. Simple fits of the relative uncertainty,

40 $$\begin{aligned} \frac{\varDelta E_{NM}}{E_{NM}}(n_\textrm{nuc},\hbox {MBPT}) = 0.07 \frac{n_\textrm{nuc}}{n_\textrm{sat}} , \end{aligned}$$

for MBPT, except MBPT-2013, and

41 $$\begin{aligned} \frac{\varDelta E_{NM}}{E_{NM}}(n_\textrm{nuc},\hbox {QMC}) = 0.21 \frac{n_\textrm{nuc}}{n_\textrm{sat}} , \end{aligned}$$

for QMC with $n_\textrm{sat}=0.16$ fm$^{-3}$ [44, 45] are also shown (dashed lines) in Fig. 10. There are two groups of uncertainties, the larger one originating from QMC calculations and the smaller one from MBPT calculations, except MBPT-2013. We have defined these two possibilities in the function defining the uncertainties in nuda toolkit. Here is an example of the use of the function uncertainty_stat() defined in the toolkit: 
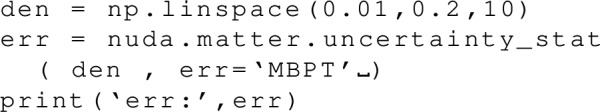
 where the variable err can be ‘MBPT’ (by default) or ‘QMC’.

**Fig. 11:**
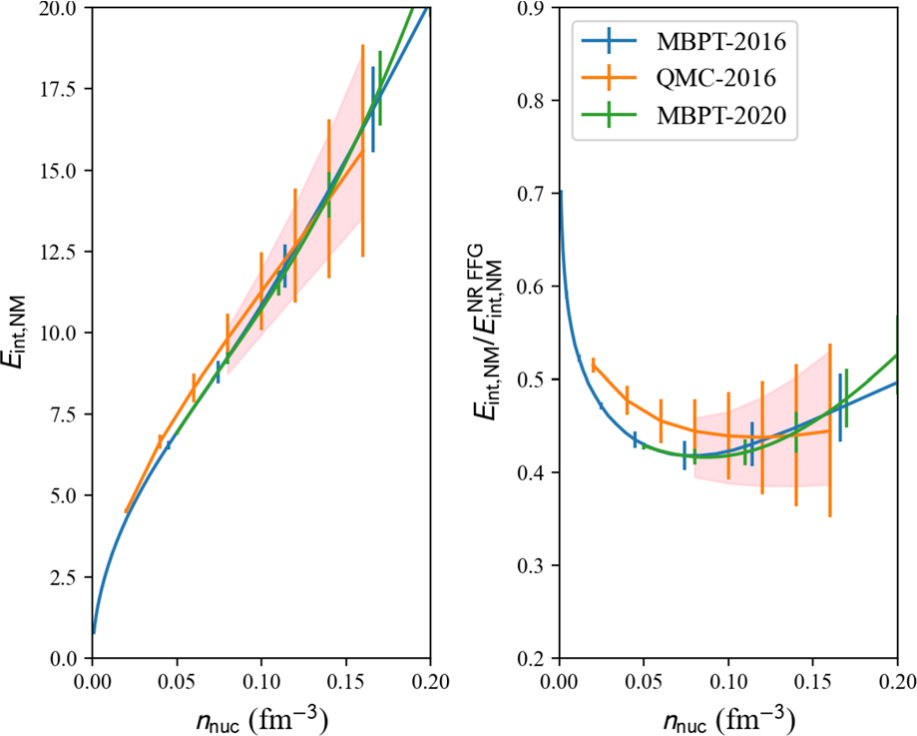
Uncertainty reference band in NM obtained from the analysis of different predictions: MBPT-2016 [38], QMC-2016 [35] and MBPT-2020 [39, 40]. Figure generated with matter_setupMicro_band_plot.py

The uncertainty originating from the many-body model is much more difficult to estimate. It has been suggested, for instance, to compare the results from different many-body techniques, while using the same interaction [46]. Since some of the many-body techniques require simplified interactions, this approach imposes that all interactions align with the simplest one. The authors of Ref. [46] confirm, however, that the tensor and spin-orbit components of the two-nucleon force and their in-medium treatment are responsible for most of the observed discrepancies among these approaches. Similar recent studies are using more realistic nuclear interactions, such as $AV_{18}$ [47, 48]. All these comparisons are very interesting, but they do not provide a simple estimation of the systematic uncertainty. In the following, we propose a method that provides results quantitatively similar to those obtained in Refs. [46–48].

Let us now explain how we obtain uncertainties in NM, SM, and for $e_\textrm{sym}$. First, we must select the models from which the uncertainty will be estimated. The users can make their own selection from different many-body techniques, such as AFDMC, QMC, BHF2, BHF23, AFQMC, and MBPT. Then the dispersion among the selected models is estimated from a Gaussian distribution,

42 $$\begin{aligned} \frac{1}{\sqrt{2\pi }\sigma } \exp \left( -\frac{(e-e_\text {cent})^2}{2\sigma ^2} \right) , \end{aligned}$$

associated to each of the model, considering the centroid $e_\text {cent}$ and the $\sigma$ obtained by each model. For the case where $\sigma$ has not been estimated, we adopt the fit (40) with the uncertainty from MBPT. The global centroids and uncertainties are then extracted from the probability distribution built upon the sum of the individual Gaussian distributions.

**Fig. 12:**
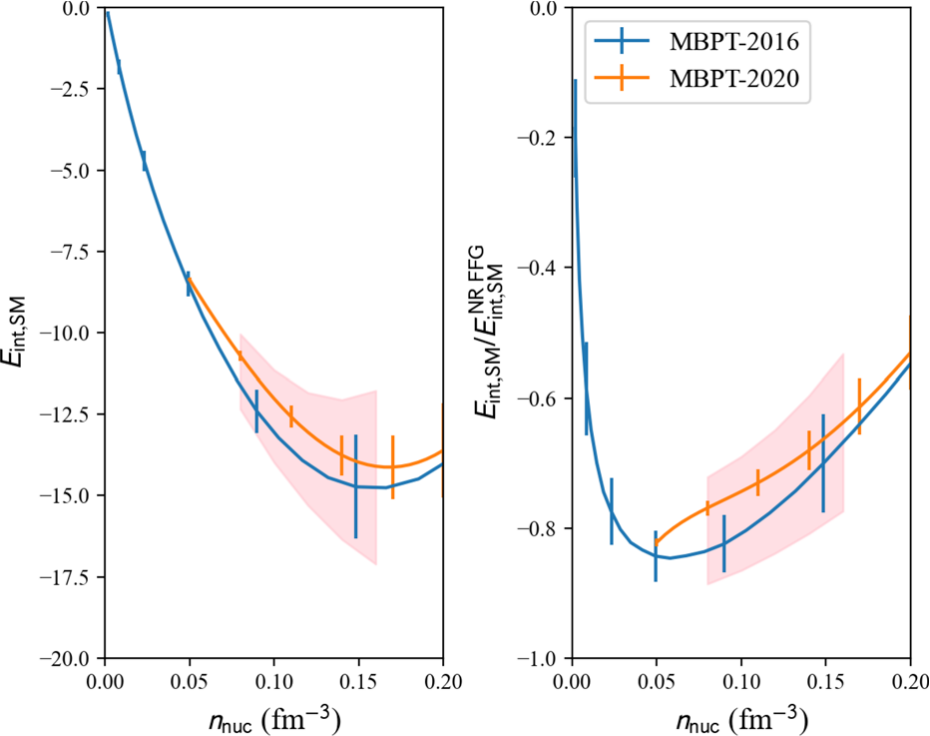
Uncertainty reference band in internal energy for SM obtained from the analysis of different predictions: MBPT-2016 [38] and MBPT-2020 [39, 40]. Figure generated with matter_setupMicro_band_plot.py

**Fig. 13:**
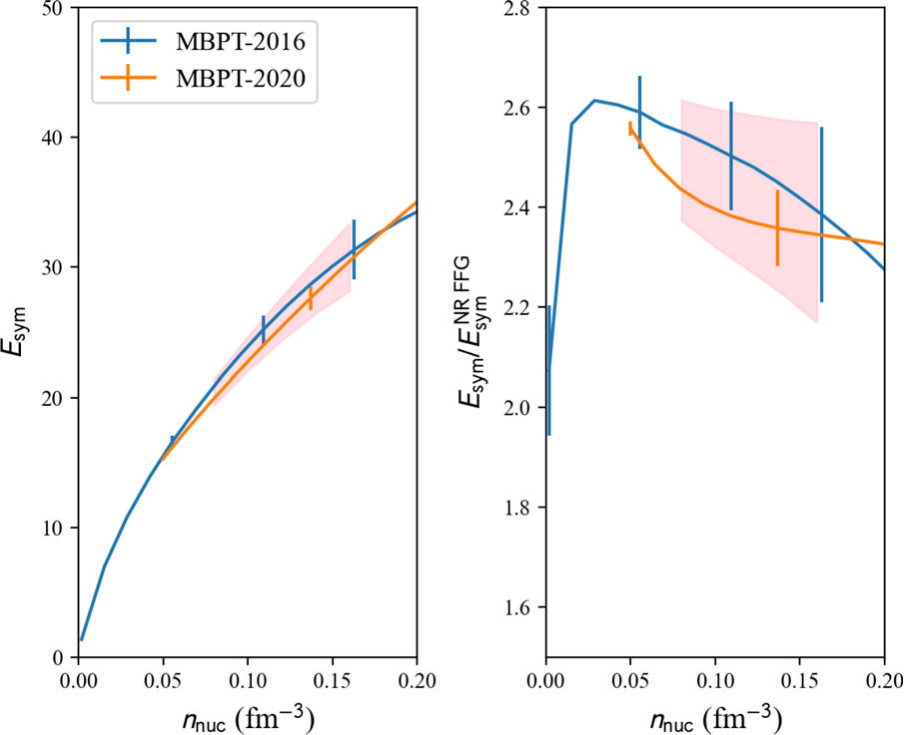
Uncertainty reference band for the symmetry energy obtained from the analysis of different predictions: MBPT-2016 [38] and MBPT-2020 [39, 40]. Figure generated with matter_setupMicro_band_plot.py

Results are shown in Fig. 11 for NM reference band, Fig. 12 for SM reference band, and Fig. 13 for the reference band representing the symmetry energy. Different microscopic calculations have been employed to define these bands. For instance, the reference band in SM does not include QMC-2016 [35], since QMC-2016 only predicts NM. We employ MBPT-2016 [38] and MBPT-2020 [39, 40] for the reference band in SM, and for the symmetry energy since NM and SM are provided in these calculations.

The reference bands shown in Figs. 11, 12 and 13 are functions of the selected models. Choosing other models will produce different reference bands. However, we believe that the choice made here is reasonable and fairly represents the present uncertainties in the prediction for SM and NM.

**Table 3:** Uncertainty reference band for the internal energy per particle in NM, in SM, and for $e_{\textrm{sym}}$ obtained using MBPT-2016, MBPT-2020, and QMC-2016 (only for the band in NM) and MBPT-2016, MBPT-2020 for the band in SM and for Esym. See text for more details

$n_\textrm{nuc}$	$e_{\textrm{NM}}$	$e_{\textrm{SM}}$	$e_{\textrm{sym}}$
(fm$^{-3}$)	(MeV)	(MeV)	(MeV)
0.08	$9.30 \pm 1.07$	$-11.2\pm 0.636$	$20.4 \pm 0.704$
0.10	$10.9 \pm 1.37$	$-12.6 \pm 0.786$	$23.3 \pm 0.938$
0.12	$12.5 \pm 1.76$	$-13.6 \pm 0.963$	$26.1 \pm 1.19$
0.14	$14.4 \pm 2.24$	$-14.2 \pm 1.19$	$28.5 \pm 1.48$
0.16	$16.2 \pm 2.83$	$-14.4 \pm 1.48$	$30.8 \pm 1.85$

A practical way to define the reference band in the nuda toolkit is now provided. The class nuda.matter. setupMicroBand() provided by nuda toolkit performs an average over various microscopic predictions, given as a list to the input models, and in the case defined by the variable matter. The following instructions: 
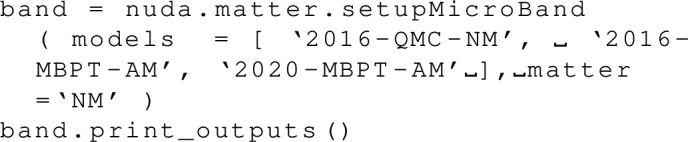
 perform an averaging over the following models: ‘2016-QMC-NM’, ‘2016-MBPT-AM’, and ‘2020-MBPT-AM’, and for NM. The input variable models contains the list of models defining the reference band and the variable matter can be set to ‘NM’ for NM, ‘SM’ for SM, and ‘Esym’ for the symmetry energy.

The class nuda.matter.setupMicroBand first analyzes the density range associated with each model and defines a new density range, avoiding extrapolation. This new interval is split into nden points (by default nden=10), where the reference band will be calculated. The class nuda.matter.setupMicroBand sums the Gaussian distribution associated with each of the input models and then computes total centroids and uncertainties, defined as standard deviation. The variable ne defines the number of points in the energy (vertical) direction (by default ne=200). The final result should be independent of the choice for ne. For the density range considered, it provides as output the centroids and uncertainties.

Here is an example where the variables nden and ne are explicitly defined: 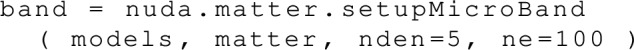


The density mesh can also be imposed by the user. For example, in the present study, we have considered the following density mesh, den=[0.08, 0.10, 0.12, 0.14, 0.16], to obtain the results shown in Table 3. In this case, however, since the density is imposed by the user, there is no check concerning extrapolation. It is the responsibility of the user to fix the density mesh in agreement with the density range of the models defining the reference band. Here is an example where the den is explicitly fixed: 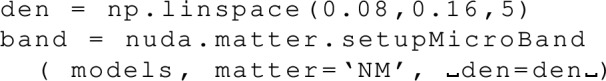


In addition, the energy boundaries (vertical) are fixed by default: e2a_min=-20 MeV and e2a_max=50 MeV. This choice can also be modified by giving explicit values for the variables e2a_min and e2a_min. Here is an example where these boundaries are explicitly fixed: 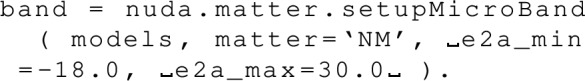


Finally, the reference band can be increased or decreased by an arbitrary factor defined in xfac. By default, xfac=1.0 in NM, xfac=1.8 in SM, and xfac=1.4 for the symmetry energy. The reason for introducing the factor xfac is to reconcile microscopic and phenomenological approaches in SM. Here is an example where the scaling factor xfac is explicitly fixed: 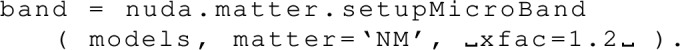


Output quantities are the following: band.den contains the densities, band.e2a_int for the internal energy per nucleon, i.e., the centroids averaging over the different input models and corresponding to the densities given in band.den, and band.e2a_std for the standard deviation among the input models.

### Check models versus a reference band

We now present another class to check models. It compares a given model and a reference band, and it replies depending on whether the model is inside the reference band or outside. To do so, the user should provide the model to check, define a reference band, and then call the class nuda.matter.setupCheck. It can be done as:
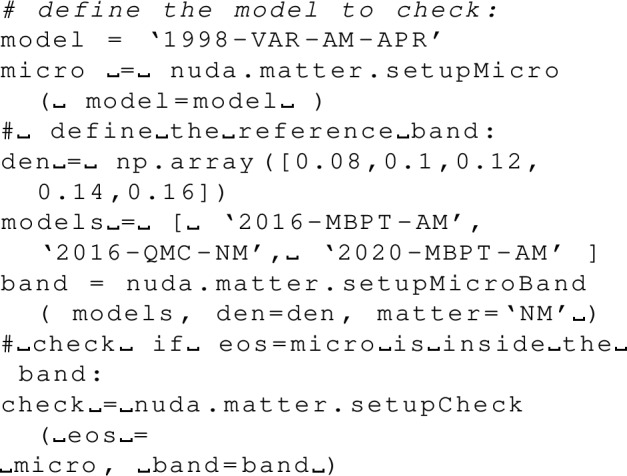
 The object check contains different properties: if the model passes inside (outside) the band, then the properties check.isInside (check.isOutside) is True (False), otherwise, it is False (True). This class has been employed, for instance, in Figs. 4, 5, 6, 7, 8 and 9 to represent the results as solid lines (if check.isInside is True) or dashed lines (if check.isInside is False).

**Fig. 14:**
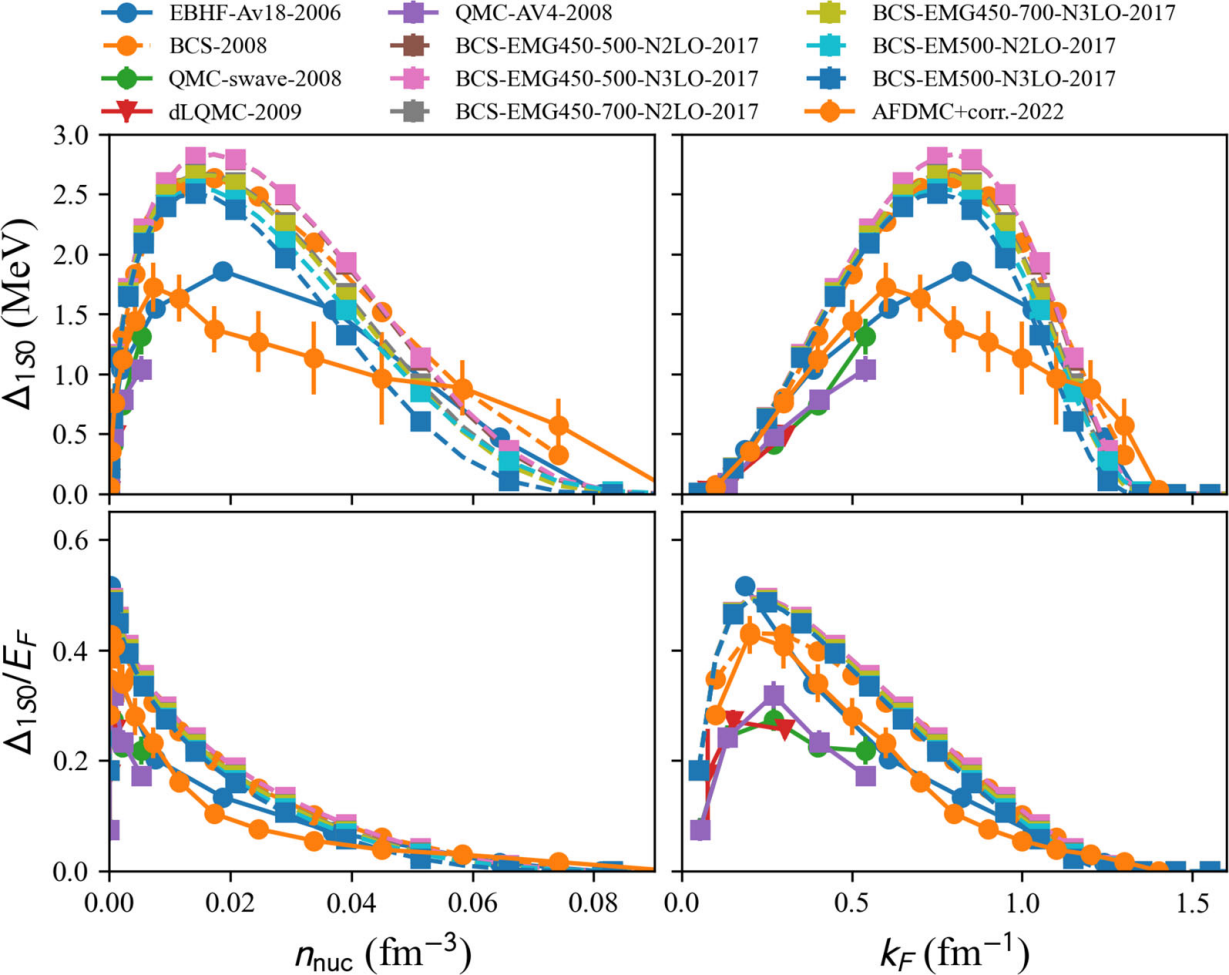
$^1$S$_0$ pairing gap in neutron matter (NM) over the Fermi energy (top) and the pairing gap (bottom) as a function of the density (left) and the neutron Fermi momentum (right) for the complete list of model available in the nuda toolkit. Figure generated with matter_setupMicro_gap_plot.py

**Fig. 15:**
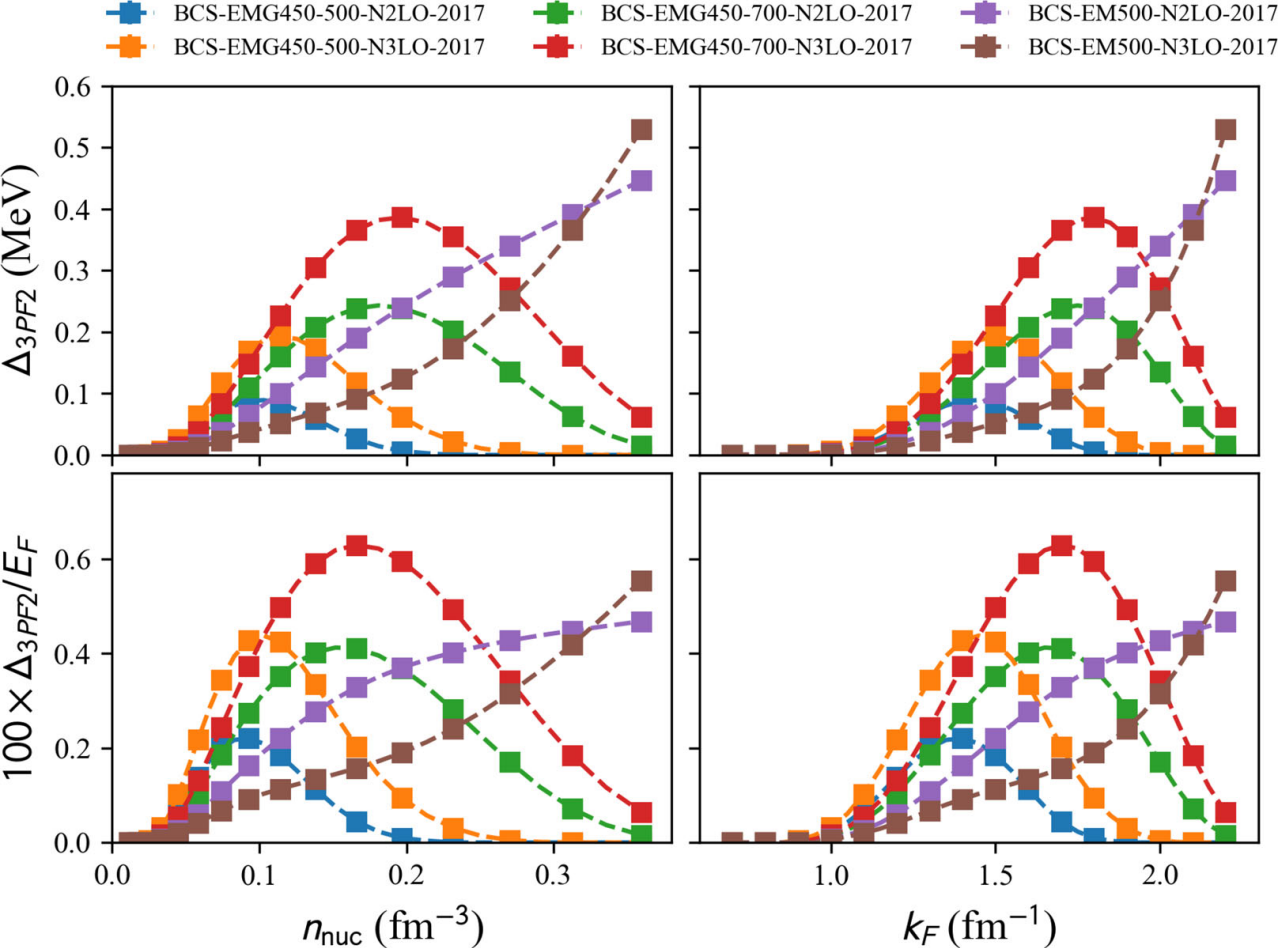
$^3$PF$_2$ pairing gap in neutron matter (NM) over the Fermi energy (top) and the pairing gap (bottom) as a function of the density (left) and the neutron Fermi momentum (right) for the complete list of model available in the nuda toolkit. Figure generated with matter_setupMicro_gap_plot.py

### Pairing gap in $^1$S$_0$ and $^3$PF$_2$ channels in NM

Pairing in nuclear systems results from the interplay between the bare nuclear force and medium polarizations, inducing correlations due to the strong bare nuclear interaction. Correlations beyond BCS are therefore non-negligible. Several many-body approaches have been employed to address the question of the strength of the pairing gap in neutron matter and symmetric matter. These many-body approaches are listed hereafter.

The nuda toolkit provides results for the pairing gap in $^1$S$_0$ and $^3$PF$_2$ channels in NM and SM. The complete list of available phenomenological models is given with the following instructions: 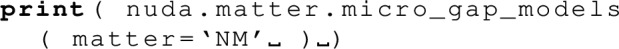 where matter can be ‘NM’ for predictions in NM, or ‘SM’ for SM. Once the variable model is chosen, the results can be obtained by calling the class matter.setupMicroGap() in the following way: 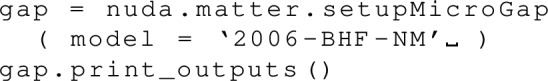 where the variable model can be chosen among the options listed hereafter.

model=‘2006-BHF-NM’:

In this calculation, vertex and self-energy corrections are treated on the same footing. A realistic two-body force (V18) is renormalized in the medium by considering RPA and dispersive corrections. Calculations are performed in NM, see Ref. [49] for more details.

model=‘2006-BHF-SM’:

The same many-body approach as model=‘2006-BHF-NM’ is performed here, except that SM is considered instead of NM, see Ref. [49] for more details.

model=‘2008-BCS-NM’:

BCS calculations based on the Argonne A8$^\prime$ two-nucleon interaction are performed in Ref. [50]. The data provided by nuda are extracted from Ref. [50], more specifically from the uncorrelated BCS case given in Table 1 for the Fermi momentum $k_{F}$, the effective mass $m_n^*/m$ and the chemical potential $\mu _n$ in NM.

model=‘2008-QMC-NM-Swave’:

Presented in Sect. 3.4.

model=‘2009-AFDMC-NM’:

Presented in Sect. 3.4.

model=‘2008-QMC-NM-AV4’:

Presented in Sect. 3.4.

**Fig. 16:**
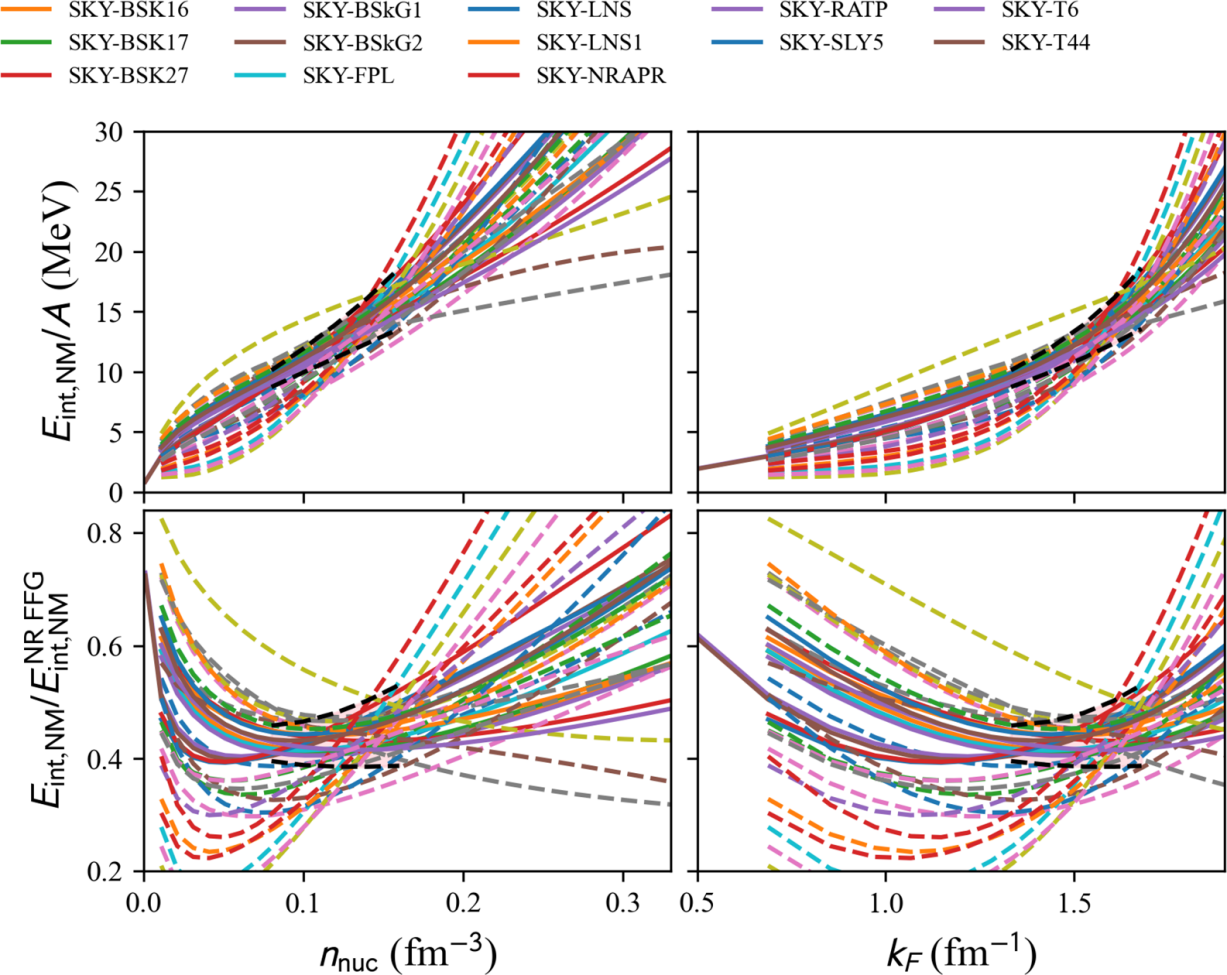
Energy in neutron matter (NM) over the free Fermi gas energy (top) and the energy per particle (bottom) as a function of the density (left) and the neutron Fermi momentum (right) for the complete list of phenomenological models based on the standard Skyrme interaction available in the nuda toolkit. Note that the Skyrme models given in the legend are those which are compatible with the reference band (this prescription applies only to this figure). Figure generated with matter_setupPheno_plot.py

**Fig. 17:**
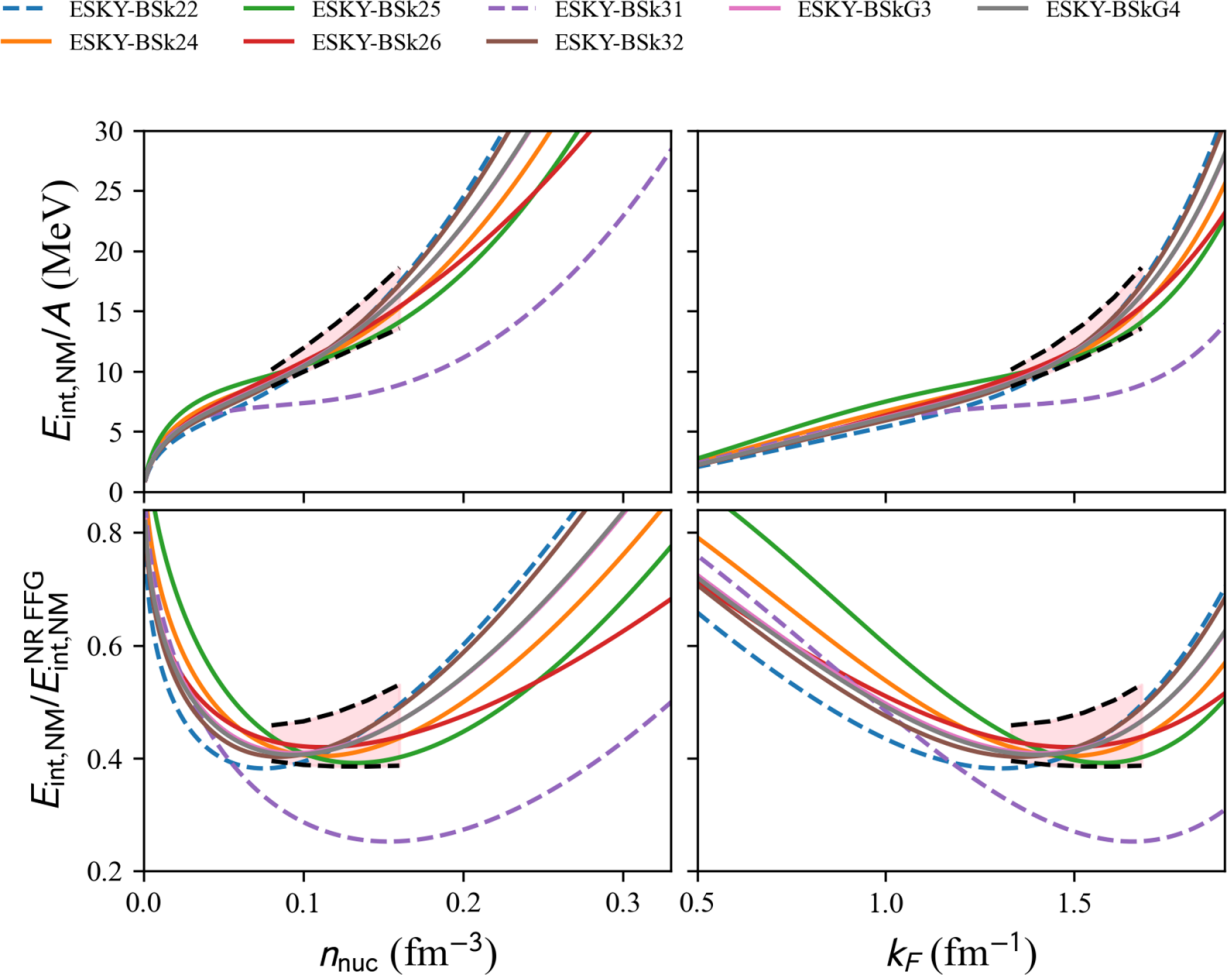
Same as Fig. 16 for the complete list of extended Skyrme EDF available in nuda toolkit. Figure generated with matter_setupPheno_plot.py

model=‘2017-MBPT-NM-GAP-EMG-450-500-N2LO’, ‘2017-MBPT-NM-GAP-EMG-450-500-N3LO’, ‘2017-MBPT-NM-GAP-EMG-450-700-N2LO’, ‘2017-MBPT-NM-GAP-EMG-450-700-N3LO’, ‘2017-MBPT-NM-GAP-EM-500-N2LO’, ‘2017-MBPT-NM-GAP-EM-500-N3LO.

Solutions of the BCS equation in the $^1$S$_0$ and $^3$PF$_2$ channels with various $\chi$EFT interactions given in Ref. [51].

model=‘2022-AFDMC-NM’:

Presented in Sect. 3.4.

Results of various calculations are shown in Fig. 14 for the pairing gap in the $^1$S$_0$ channel in NM. Among these results, the ones labeled as BCS (in dashed lines) are performed by solving the lowest-order BCS gap equation. They predict a pairing gap peaked at $2.75\pm 0.15$ MeV for $k_{F}\approx 0.8$ fm$^{-1}$. The other calculations (in solid lines) consider correlations beyond the bare BCS ones using different many-body techniques, see details here before. Interestingly, all calculations, including correlations beyond BCS, predict a reduction of the pairing gap.

Results of various calculations for the pairing gap in the $^3$PF$_2$ channel in NM are shown in Fig. 15. All the calculations shown in Fig. 15 are performed at the BCS approximation using various $\chi$EFT nuclear interactions. The pairing gaps are small, in absolute values, but there is a strong model dependence of the results.

### Phenomenological models

The complete list of available phenomenological models is given with the following instruction: 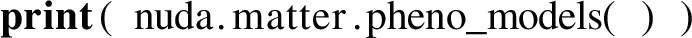


The nuda toolkit provides results for the following models: ‘Skyrme’, ‘ESkyrme’, ‘NLRH’, ‘DDRH’, and ‘DDRHF’.

The complete list of available phenomenological parametrizations for a given model is given with the following instruction: 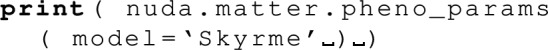 Once the variables model and param are chosen, the results can be obtained by calling the class matter.setupPheno() in the following way: 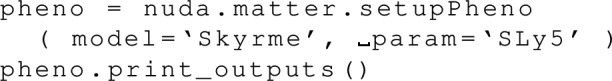 The object pheno contains a large set of properties, some of them listed by the command pheno.print_outputs(). The entire list of properties is obtained in the following way: 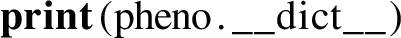


Output quantities are, for instance: pheno.nm_e2a (pheno.nm_e2a_int) and pheno.sm_e2a (pheno.sm_e2a_int) for the nucleon (internal) energy per particle in NM and SM.

#### Skyrme EDFs

Skyrme EDFs are obtained from the Skyrme contact nuclear interaction complemented with density-dependent terms. Reviews can be found in the following Refs. [52, 53].

A typical call for a given model and parameter set param, see hereafter, is: 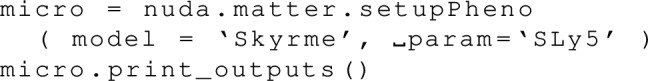 Each calculation can be obtained by fixing the variables model and param to one of the following values:

model=‘Skyrme’.

param= ‘BSK14’ [54], ‘BSK16’ [55], ‘BSK17’ [56], ‘BSK27’ [57], ‘BSkG1’ [58], ‘BSkG2’ [59], ‘F-’ [60], ‘F+’ [60], ‘F0’ [60], ‘FPL’, ‘LNS’ [26], ‘LNS1’ [61], ‘LNS5’ [61], ‘NRAPR’ [62], ‘RATP’ [63], ‘SAMI’ [64], ‘SGII’ [65], ‘SIII’ [66], ‘SKGSIGMA’ (Gs) [67], ‘SKI2’ [68], ‘SKI4’ [68], ‘SKMP’ [69], ‘SKMS’ [70], ‘SKO’ [71], ‘SKOP’ [71], ‘SKP’ [72], ‘SKRSIGMA’ (Rs) [67], ‘SKX’ [73], ‘Skz2’ [74], ‘SLY4’ [75], ‘SLY5’ [75], ‘SLY230A’ [76], ‘SLY230B’ [76], ‘SV’ [66], ‘T6’ [77], ‘T44’ [78], ‘UNEDF0’ [79], ‘UNEDF1’ [80].

model=‘ESkyrme’.

param= ‘BSk22’ [81], ‘BSk24’ [81], ‘BSk25’ [81], ‘BSk26’ [81], ‘BSk31’ [82], ‘BSk32’ [82], ‘BSkG3’ [83], ‘BSkG4’ [84].

**Fig. 18:**
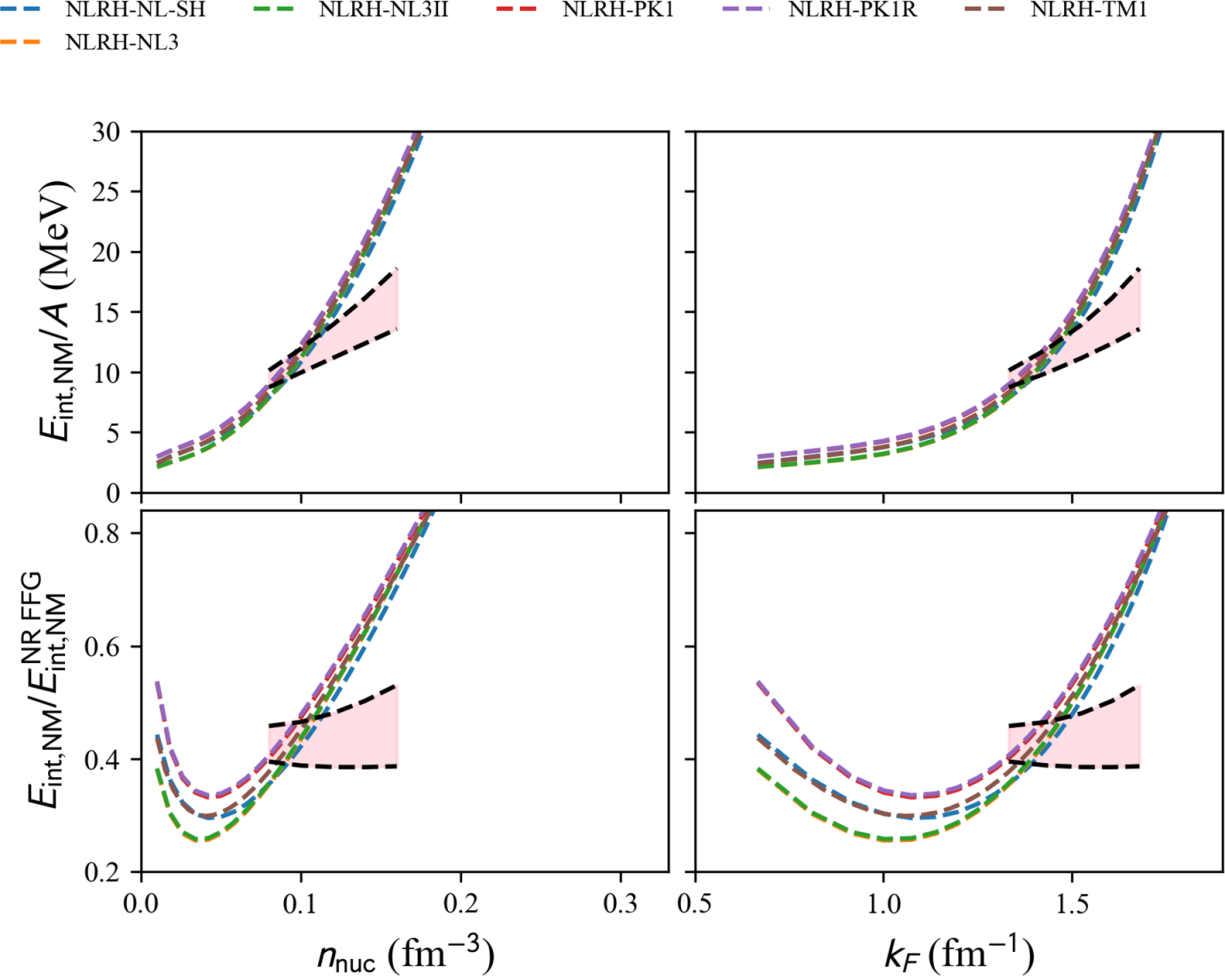
Same as Fig. 16 for the complete list of NLRH EDF available in nuda toolkit. Figure generated with matter_setupPheno_plot.py

Results in NM are shown in Fig. 16 for Skyrme EDFs and Fig. 17 for extended Skyrme EDFs. For Skyrme EDFs, the legend only names the models passing the reference band, since there is a long list of models. For extended Skyrme EDFs, as well as for other models presented hereafter, the entire list of models available in the nuda toolkit is given in the legend of the figures.

#### Non-linear RH (NLRH)

The nonlinear relativistic Hartree approaches, often referred to as relativistic mean-field (RMF) approaches, are an extension of the original Walecka model [85], considering nonlinear meson-nucleon couplings. Nonlinear couplings were introduced initially to soften the EoS and reduce the incompressibility modulus at saturation density. A typical call for a given model and parameter set param, see hereafter, is: 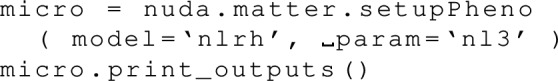 Each calculation can be obtained by fixing the variables model and param to one of the following values:

model=‘NLRH’.

param=‘NL-SH’ [86], ‘NL3’ [87], ‘NL3s’ [87], ‘PK1’ [88], ‘PK1R’ [88], ‘TM1’ [89].

Results in NM are shown in Fig. 18 for NLRH EDFs. The NLRH models available in the nuda toolkit are all represented, and it is interesting to note that they all disagree with the reference band previously discussed. Figure 18 shows that all NLRH are more repulsive than expectations from the NM reference band.

**Fig. 19:**
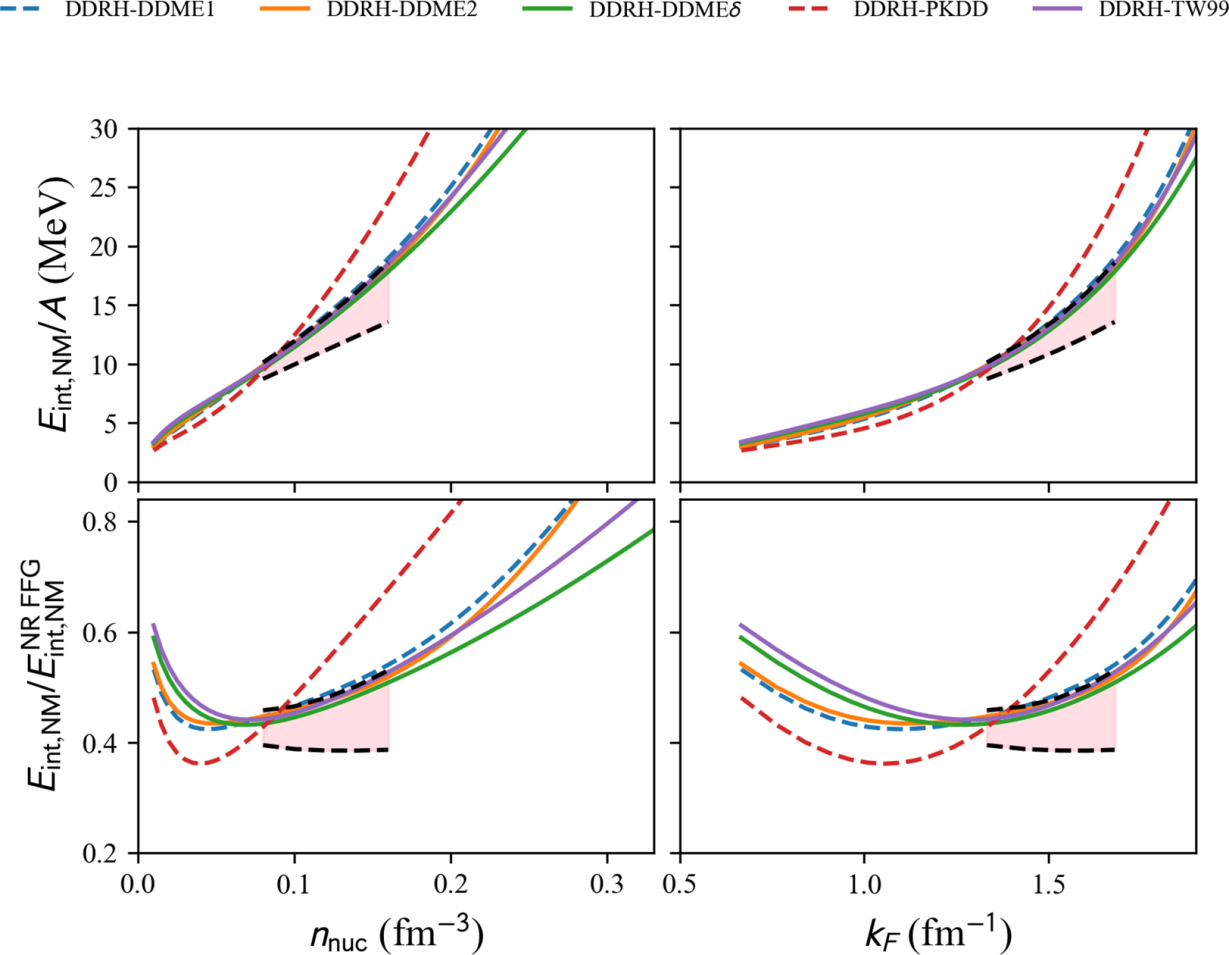
Same as Fig. 16 for the complete list of DDRH EDF available in nuda toolkit. Figure generated with matter_setupPheno_plot.py

#### Density-dependent RH (DDRH)

The density-dependent relativistic Hartree approach has emerged after the NLRH one. It consists of considering density-dependent coupling constants instead of the non-linear meson-nucleon coupling in the NLRH model. These density-dependent coupling constants are adjusted to mimic Dirac-Brueckner–Hartree–Fock results, and they produce softer EoS similar to the NLRH approach.

A typical call for a given model and parameter set param, see hereafter, is: 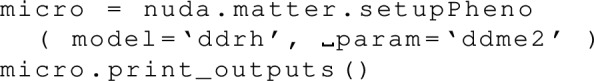 Each calculation can be obtained by fixing the variables model and param to one of the following values:

model=‘DDRH’.

param=‘DDME1’ [90], ‘DDME2’ [91], ‘DDMEd’ (DD-ME$\delta$) [92], ‘PKDD’ [88], ‘TW99’ [93].

Results in NM are shown in Fig. 19 for DDRH EDFs. The relativistic DDRH models available in the nuda toolkit are all represented. The density-dependent coupling constant softens the energy per particle, and among the models plotted in Fig. 19, three are in agreement with the reference band previously discussed: TW99, DDME$\delta$, and DDME2. The model DDME1 is very close to the reference band, while the model PKDD is the most repulsive one.

**Fig. 20:**
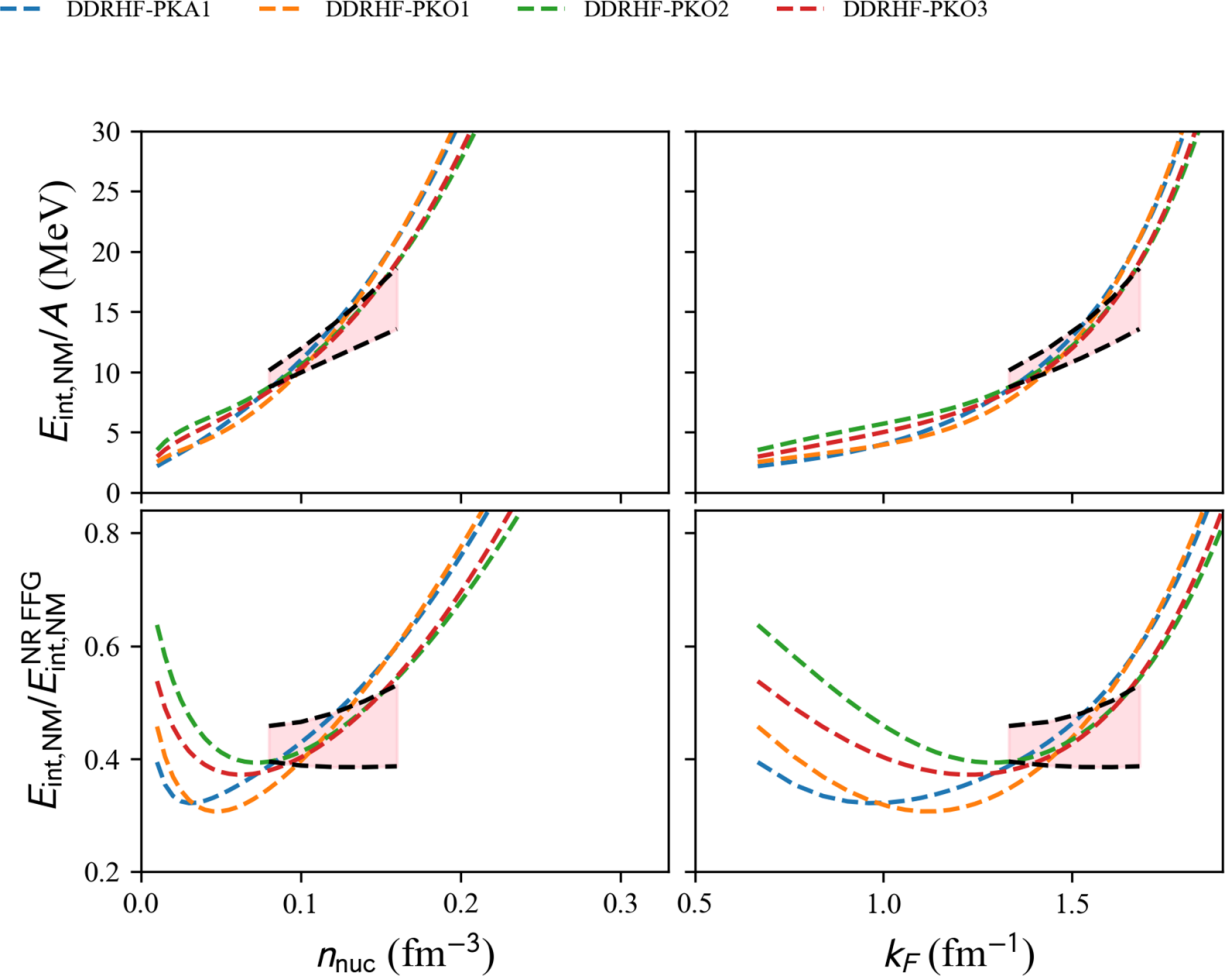
Same as Fig. 16 for the complete list of DDRHF EDF available in nuda toolkit. Figure generated with matter_setupPheno_plot.py

#### Density-dependent RHF (DDRHF)

The density-dependent relativistic Hartree–Fock (DDRHF) approach adds the Fock term in the mean field to the DDRH model. By introducing the Fock term, it allows contributions from pions and rho-tensors in uniform matter.

A typical call for a given model and parameter set param, see hereafter, is: 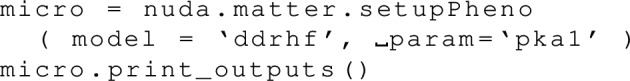 Each calculation can be obtained by fixing the variables model and param to one of the following values:

model=‘DDRHF’.

param=‘PKO1’ [94], ‘PKO2’ [95], ‘PKO3’ [95], ‘PKA1’ [96].

Results in NM are shown in Fig. 20 for DDRH EDFs. The relativistic DDRH models available in the nuda toolkit are all represented. The models represented are not compatible with the reference band, but they are less repulsive in average than the previously represented NLRH models. The impact of the Fock term is to make the energy per particle more repulsive than DDRH models shown in Fig. 19. Among the models shown in Fig. 20, PKO2 and PKO3 are the closest to the reference band.

### Energy per particle in NM and SM from a meta-analysis

We now collect all microscopic and phenomenological models’ predictions available in the toolkit to perform meta-analyses.

**Fig. 21:**
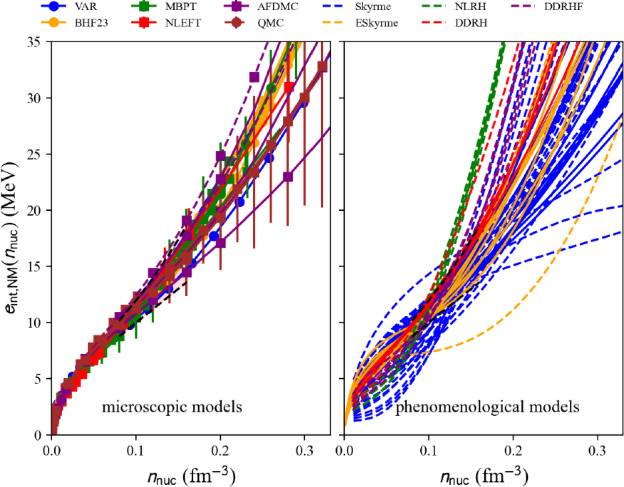
Internal energy per nucleon in NM for the list of microscopic (left) and phenomenological (right) models available in the nuda toolkit. Figure generated with matter_all_plot.py

A typical call for given input variables, see above, is: 
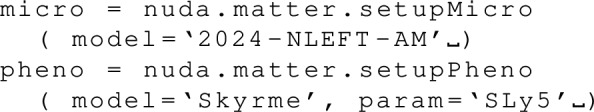
 The energy per nucleon in SM and NM is defined as an attribute of the objects micro and pheno: 
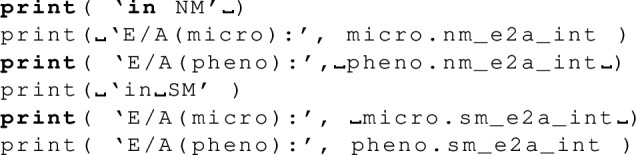


In Fig. 21 the model predictions for the internal energy per nucleon in NM for microscopic approaches (left) and phenomenological ones (right) as a function of the nucleon density are compared. Models belonging to the same many-body approach (mb, micro) or model (model, pheno) are shown with the same color. We use the class nuda.matter. setupCheck() to distinguish between the model passing inside the reference band in NM (solid line) from the others being outside (dashed line): all the models drawn in Fig. 21 in solid line pass through the reference band in NM. Almost all microscopic models pass through the reference band in NM, while it selects a lot of phenomenological models.

**Fig. 22:**
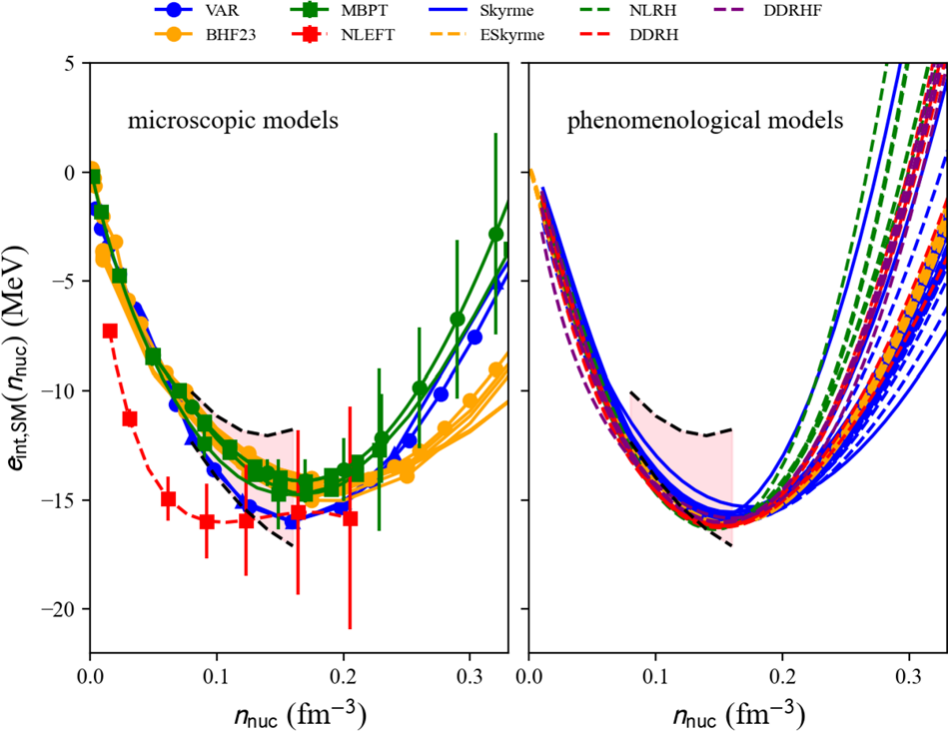
Same as Fig. 21 for SM. Figure generated with matter_all_plot.py

We now represent in Fig. 22 a comparison of the internal energy per nucleon in SM predicted by microscopic approaches (left) and phenomenological ones (right) as a function of the nucleon density. Models belonging to the same many-body approach (micro) or model (pheno) are shown with the same color. Similarly to the NM case, the solid lines in Fig. 22 refer to models passing inside the reference band, but now defined in SM. NLEFT predicts that the internal energy per nucleon in SM is more attractive at low density than the reference band. This is most probably due to the strong deuteron correlation in low-density symmetric matter. In NLEFT, there are indeed cluster correlations in the ground state that are present in the results. The dispersion between the phenomenological model is much reduced in SM, compared to NM. The reference band remains, however, quite selective, especially at low density.

### Symmetry energy in uniform matter

The symmetry energy is defined as, see also Eq. (14),

43 $$\begin{aligned} e_\textrm{sym}(n_\textrm{nuc}) = e_{\textrm{NM}}(n_\textrm{nuc})-e_{\textrm{SM}}(n_\textrm{nuc}) . \end{aligned}$$

The calculation of the symmetry energy requires the energy per nucleon in SM and NM. The microscopic models providing results in NM and SM can be listed in the following way (for a given choice of micro_mb inside the list provided by micro_mbs): 
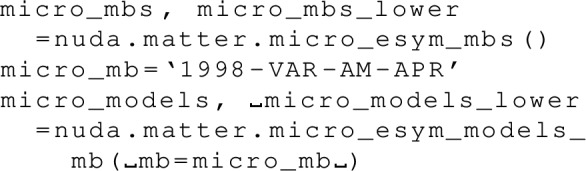
 Similarly, for phenomenological models: 
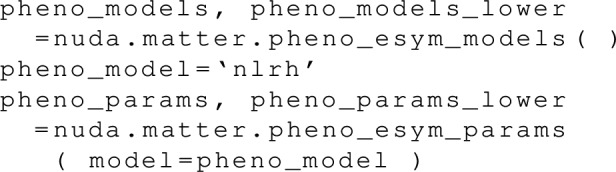


One should then select the variables micro_model in the list micro_models, and pheno_param from pheno_params to obtain the symmetry energy from the microscopic and phenomenological models as: 
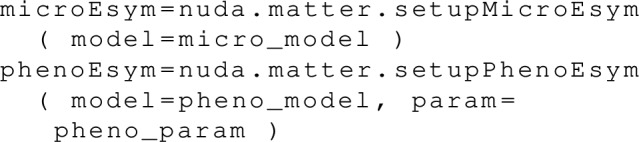


The symmetry energy is defined as an attribute of the microscopic and phenomenological models: microEsym. esym and phenoEsym.esym. These quantities are calculated for a density mesh defined in microEsym.den and phenoEsym.den.

**Fig. 23:**
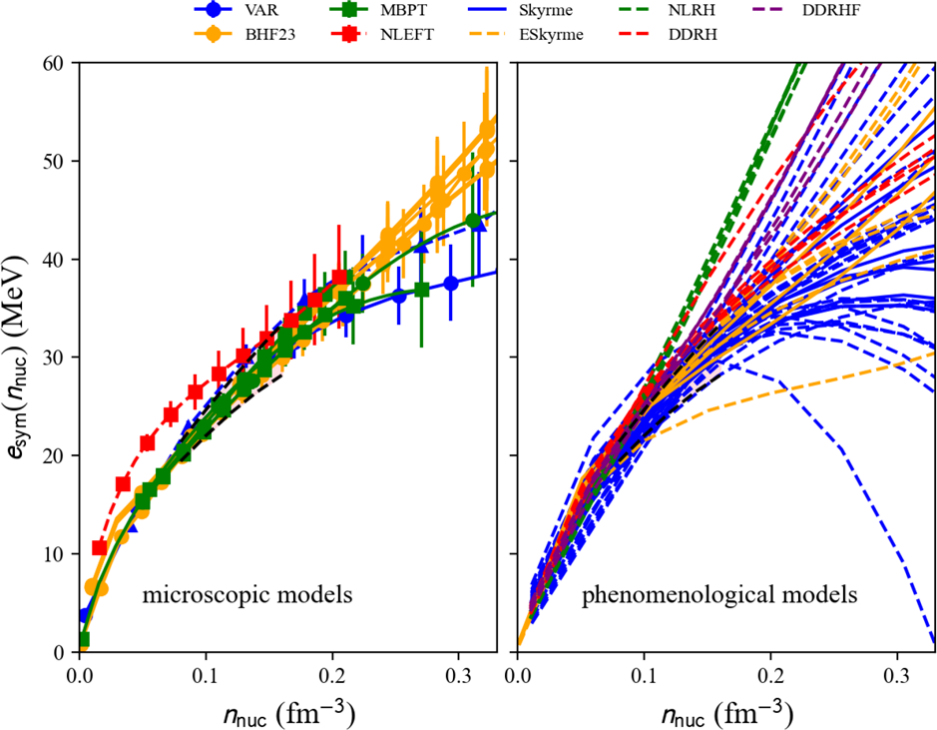
Same as Fig. 21 for $e_\textrm{sym}$. Figure generated with matter_all_plot.py

The symmetry energy is shown in Fig. 23 for the microscopic models providing results in SM and NM. The models compatible with the reference band are shown as solid lines, while the others are shown as dashed lines. NLEFT predicts a large symmetry energy at low density, as expected from its prediction in SM. The prediction for the symmetry energy from NLEFT is outside the reference band. The other microscopic predictions are quite consistent, while the phenomenological predictions for the symmetry energy are quite dispersed above saturation density. This reflects the large dispersion among the phenomenological models in NM.

### Nucleon pressure in NM and SM

The pressure measures the change in energy resulting from a modification of the volume, or density, of matter at constant number of particles. In uniform matter, the nucleon pressure is defined as:

44 $$\begin{aligned} p_\textrm{nuc}(n_\textrm{nuc}) = n_\textrm{nuc}^2\frac{\partial e_\textrm{nuc}(n_\textrm{nuc},\delta )}{\partial n_\textrm{nuc}}\Bigr |_{\delta } . \end{aligned}$$

The nucleon pressure is related to the first derivative of the energy per nucleon, discussed in previous sections. Pressure is a thermodynamic property of the system that counterbalances against compression, e.g., due to gravity, providing the stability of neutron stars. It is therefore important to calculate the pressure.

**Fig. 24:**
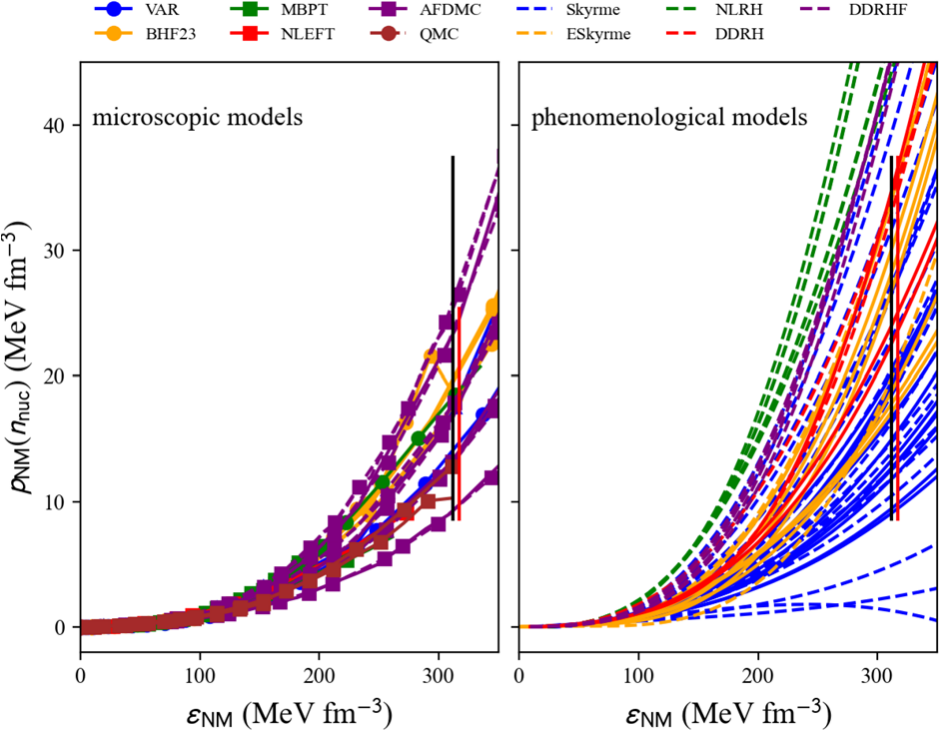
Pressure in NM as a function of the energy density $\epsilon$ for the list of microscopic (left) and phenomenological (right) models available in the nuda toolkit. Figure generated with matter_all_plot.py

A typical call for given input variables, see above, is: 
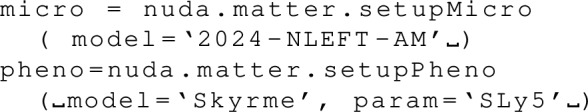
 The nucleon pressure in SM and NM is defined as an attribute of the objects micro and pheno: 
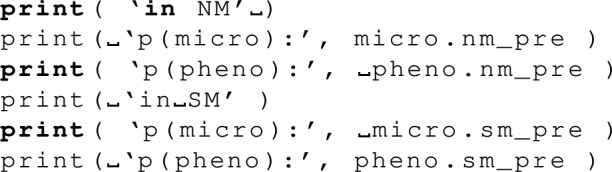


Figure 24 shows a comparison of the nucleon pressure in NM predicted by microscopic approaches (left) and phenomenological ones (right) as a function of the nucleon energy density. Models belonging to the same many-body approach (micro) or model (pheno) are shown with the same color. Solid lines are associated with models that satisfy the reference band in NM, while dashed lines are for the models that disagree with the reference band in NM. We are interested in the prediction for the nucleon pressure at twice saturation energy-density ($\epsilon _\textrm{sat}\approx 155$ MeV fm$^{-3}$). The vertical bars represent the dispersion between the model predictions, for those in agreement with the reference band in NM. The red bars are different for microscopic and phenomenological models, while the black ones are identical for both cases and include the red ones. Note that the dispersion is larger for the phenomenological models compared to the microscopic ones. This was already observed for the energy per particle in NM, see Fig. 21 for instance.

**Fig. 25:**
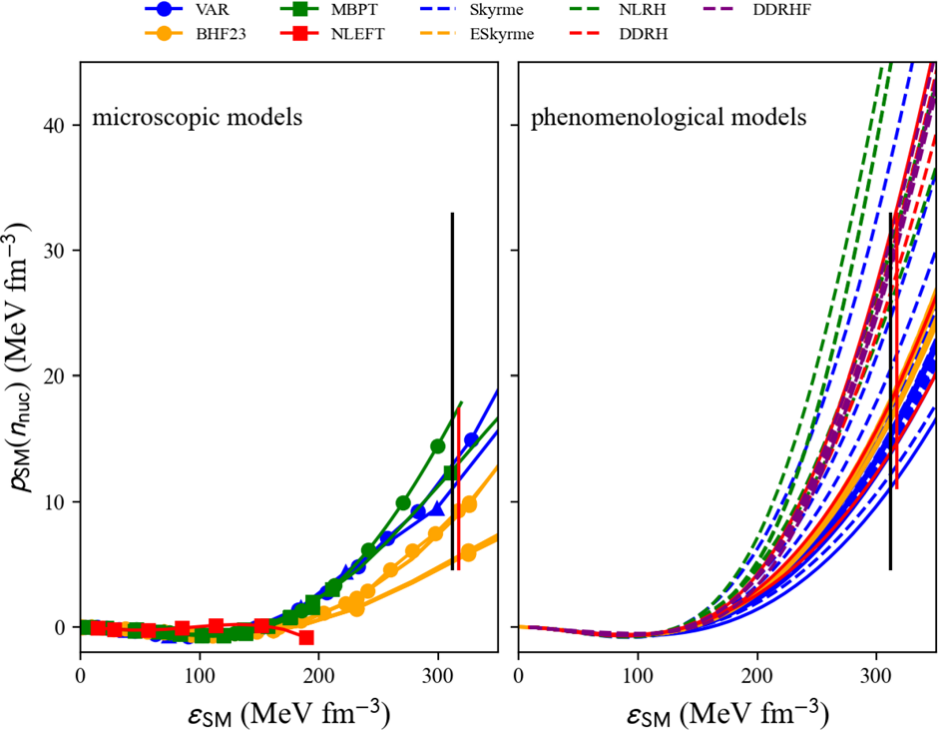
Same as Fig. 24 for SM. Figure generated with matter_all_plot.py

Figure 25 is similar to Fig. 24, except that it shows the pressure in SM instead of NM. The reference band in NM is still employed to separate the models in solid lines from the ones in dashed lines. While the model dispersion is larger for the phenomenological models compared to the microscopic ones (as in NM), the softest EoS predicted by microscopic models (BHF23) are not present in the phenomenological models encoded in the nuda toolkit. It could be due to the finite and low number of phenomenological models in the toolkit. It therefore illustrates that it is necessary to combine microscopic and phenomenological models for our meta-analysis. The vertical bars are constructed as explained in the discussion of Fig. 24. The size of the bars in NM and SM is reported in Table 7, discussed in Sect. 8.3.

### Nuclear empirical parameters

The nuclear empirical parameters (NEP) are defined as the derivatives for the energy per nucleon in SM, $e_\textrm{SM}$, and the symmetry energy $e_\textrm{sym}$ in the following way:

45 $$\begin{aligned} P^N_{\textrm{sat}}= &   \left( 3n_\textrm{sat}\right) ^N \frac{\partial ^N e_\textrm{SM}(n_\textrm{nuc})}{\partial n_\textrm{nuc}^N}\Bigr |_{n_\textrm{sat}} , \end{aligned}$$

46 $$\begin{aligned} P^N_{\textrm{sym}}= &   \left( 3n_\textrm{sat}\right) ^N \frac{\partial ^N e_\textrm{sym}(n_\textrm{nuc})}{\partial n_\textrm{nuc}^N}\Bigr |_{n_\textrm{sat}} , \end{aligned}$$

where $P=(E,L,K,Q,Z)$. We have $L_\textrm{sat}=0$, since the value of $n_\textrm{sat}$ is defined as the density for which the pressure in SM is zero.

The complete list of models for which NEP is available is provided in the following way: 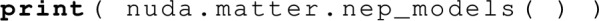 For the moment, only phenomenological models are listed.

A typical call for a given model and parameter set param, see hereafter, is: 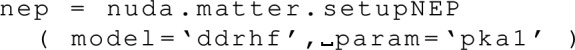 For the choice of variables model and param, the NEP can be obtained in the following way: 
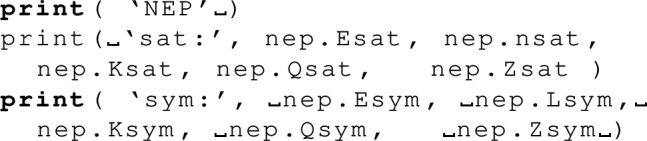


The statistical distribution of NEP for a given model can be obtained by calling the class nuda.matter. setupNEPStat_model() in the following way: 
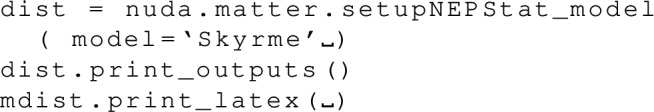
 It is performed in the sample script matter_setupNEP Stat_script.py.

**Fig. 26:**
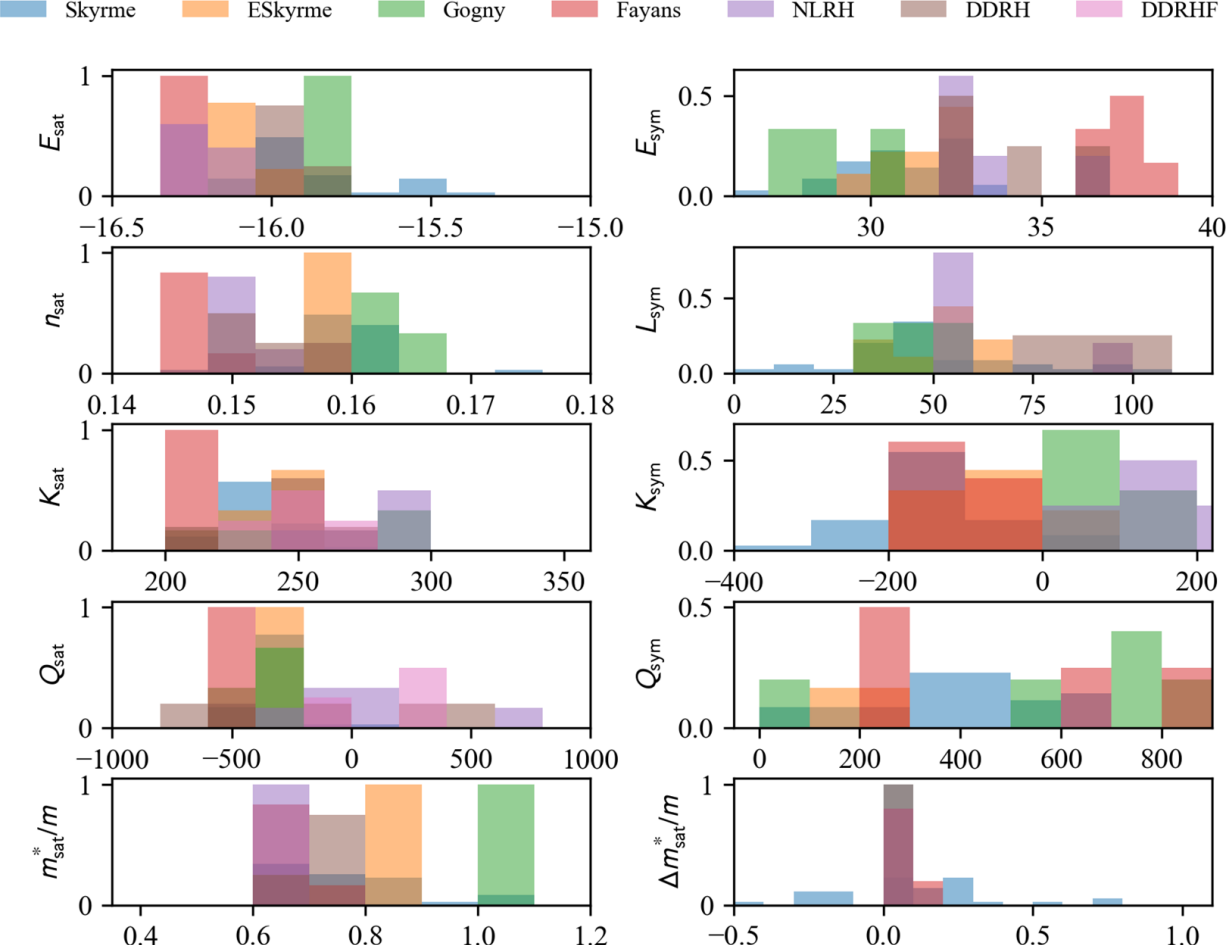
Distribution of NEP for phenomenological models available in the nuda toolkit. Figure generated with matter_setupNEPDiststats_plot.py

**Table 4:** Centroids and standard deviations averaging over several models from the nuda toolkit. This table has been generated with matter_setupNEPstat_script.py and matter_setupNEPStats_script.py

NEP	$E_{\textrm{sat}}$	$n_{\textrm{sat}}$	$K_{\textrm{sat}}$	$Q_{\textrm{sat}}$	$Z_{\textrm{sat}}$	$E_{sym}$	$L_{sym}$	$K_{sym}$	$Q_{sym}$	$Z_{sym}$	$m^*_{sat}/m$	$\varDelta m^*_{sat}/m$
	MeV	fm$^{-3}n$	MeV	MeV	MeV	MeV	MeV	MeV	MeV	MeV		
Skyrme (38): ‘BSK14’, ‘BSK16’, ‘BSK17’, ‘BSK27’, ‘BSkG1’, ‘BSkG2’,‘F-’, ‘F+’, ‘F0’, ‘FPL’, ‘LNS’,												
‘LNS1’, ‘LNS5’, ‘NRAPR’, ‘RATP’, ‘SAMI’, ‘SGII’, ‘SIII’, ‘SKGSIGMA’, ‘SKI2’, ‘SKI4’, ‘SKMP’, ‘SKMS’,												
‘SKO’, ‘SKOP’, ‘SKP’, ‘SKRSIGMA’, ‘SKX’, ‘Skz2’, ‘SLY4’, ‘SLY5’, ‘SLY230A’, ‘SLY230B’, ‘SV’, ‘T6’,												
‘T44’, ‘UNEDF0’, ‘UNEDF1’.												
Centroid	− 15.89	0.159	238.0	− 350	1445	30.88	49.3	− 135	372	− 2180	0.77	0.126
Std.dev.	0.18	0.004	25.7	86	494	1.52	21.2	88	184	1044	0.14	0.300
GSkyrme (5): ‘SkK180’, ‘SkK200’, ‘SkK220’, ‘SkK240’, ‘SkKM’.												
Centroid	− 15.83	0.156	212.0	− 936	–	30.00	16.2	− 368	195	–	1.10	1.876
Std.dev.	0.03	0.003	20.4	677	–	0.00	13.7	114	249	–	0.00	0.000
ESkyrme (8): ‘BSk22’, ‘BSk24’, ‘BSk25’, ‘BSk26’, ‘BSk31’, ‘BSk32’, ‘BSkG3’, ‘BSkG4’.												
Centroid	− 16.08	0.158	241.7	− 312	–	31.00	52.3	− 53	–	–	0.83	0.000
Std.dev.	0.03	0.000	3.3	35	–	1.00	9.7	64	–	–	0.03	0.000
NLRH (6): ‘NL-SH’, ‘NL3’, ‘NL3II’, ‘PK1’, ‘PK1R’, ‘TM1’.												
Centroid	− 16.28	0.147	288.3	98	5734	37.40	116.2	72	24	− 3218	0.68	0.062
Std. dev.	0.03	0.002	31.2	271	2920	0.80	3.8	26	129	940	0.01	0.002
DDRH (5): ‘DDME1’, ‘DDME2’, ‘DDMEd’, ‘PKDD’, ‘TW99’.												
Centroid	− 16.20	0.152	243.8	− 121	4248	33.48	61.1	− 102	578	− 4206	0.66	0.064
Std. dev.	0.06	0.001	14.7	473	390	1.73	14.8	17	296	1970	0.02	0.033
DDRHF (4): ‘PKA1’, ‘PKO1’, ‘PKO2’, ‘PKO3’.												
Centroid	− 15.97	0.154	248.1	389	5269	33.97	90.0	128	523	− 9955	0.73	0.015
Std. dev.	0.08	0.004	11.6	350	838	1.37	11.1	51	237	4156	0.03	0.003
Total (74): ‘Skyrme’, ‘GSkyrme’, ‘ESkyrme’, ‘Gogny’, ‘Fayans’, ‘NLRH’, ‘DDRH’, ‘DDRHF’.												
Centroid	− 15.97	0.157	242.1	− 298	2542	31.67	56.3	− 103	349	− 3141	0.79	0.248
Std. dev.	0.20	0.005	29.0	369	2058	2.52	29.2	137	251	2670	0.15	0.553

The statistical distribution of NEP is shown in Fig. 26 for the model available in the nuda toolkit. The weights of the models have been adjusted to make this distribution independent of the number of parameter sets for each model. For low-order NEP, such as $E_\textrm{sat}$, $n_\textrm{sat}$, and $E_\textrm{sym}$, the predictions of the phenomenological models are rather well grouped, even for $E_\textrm{sym}$, for which the distribution is the broadest. Then comes $K_\textrm{sat}$ and $L_\textrm{sym}$, which are less well-known. The NEP $Q_\textrm{sat}$, $K_\textrm{sym}$, and $Q_\textrm{sym}$ are yet totally unknown. Note, however, that for $Q_\textrm{sat}$ the distribution for non-relativistic models is different from the distribution from relativistic ones: the NR models prefer a large and negative value for $Q_\textrm{sat}$ while the relativistic models prefer $Q_\textrm{sat}$ to be large and positive. Such a difference between relativistic and NR models has already been noted in Ref. [44], and could also be at the origin of the different values for $K_\textrm{sat}$ that these models prefer [97–99].

The statistical analysis summing over a set of models is performed in the sample script matter_setupNEPStats_script.py: 
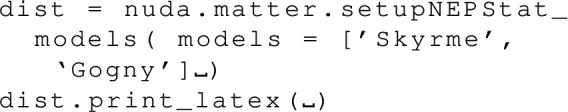
 The input variable models contains a list of models for which we want to perform the statistical averaging. It can be a choice of a few models, as shown in the previous example, or it can be made over all models available in the nuda toolkit. It the latter case, the script is: 
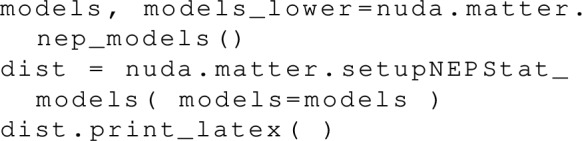


Results are shown in Table 4, where the average of the NEP for each class of phenomenological model is provided, as well as a global average over all available models (meta-average). The NEPs $Q_\textrm{sat}$ and $K_\textrm{sym}$ are found to be very model-dependent. The centroid for $Q_\textrm{sat}$ is negative for all non-relativistic models, and it is found to be positive for relativistic models, except ‘DDRH’ ones, for which the distribution is compatible with zero. For $K_\textrm{sym}$, the distribution of non-relativistic models is peaked at negative values, while relativistic models, except ‘DDRH’ predict it to be positive. Since there is a large number of non-relativistic Skyrme models in the nuda toolkit, the meta-analysis is influenced by their prediction, and the peaks for $Q_\textrm{sat}$ and $K_\textrm{sym}$ are negative. However, the standard deviation is large, reflecting the contribution of relativistic models for these parameters.

The Landau effective mass and the splitting of the Landau effective mass are introduced in the following Sect. 3.13. For most of the models, the effective mass $m_\textrm{sat}^*/m$ is lower than 1.0, except for the generalized Skyrme models (GSkyrme) for which it is fixed to be 1.1. The splitting of the effective mass is peaked at positive values, but its standard deviation reflects the fact that many models predict it to be negative.

### Landau effective mass

The nuda toolkit provides the effective mass calculated by the authors (when it is calculated). It is related to the group velocity as:

47 $$\begin{aligned} \frac{m_q}{m_q^*} = \frac{m_q}{\hbar ^2} \left( \frac{1}{k}\frac{d e_q(k)}{d k}\right) \Bigr |_{k=k_{F_q}} , \end{aligned}$$

where $e_q(k)$ is the single particle energy defined as

48 $$\begin{aligned} e_q(k) = \frac{\hbar ^2 k^2}{2m_q}+\varSigma _q(k)+\varSigma _{q,0} , \end{aligned}$$

where $\varSigma _q(k)$ is the momentum dependent self-energy. The quantity $m_q^*$ is also called the Landau effective mass.

The effective mass is often represented in terms of two quantities $\kappa _\textrm{sat}$ ($=\kappa _s$) and $\kappa _\textrm{sym}$, where $\kappa _\textrm{sym}=\kappa _\textrm{sat}-\kappa _v$, where $\kappa _v$ is the enhancement factor of the Thomas-Reiche-Kuhn sum rule [100]. We have:

49 $$\begin{aligned} \frac{m_q}{m^*_q(n_\textrm{nuc},\delta )} = 1 + \left( \kappa _\textrm{sat}+\tau _3 \kappa _\textrm{sym}\delta \right) \frac{n_\textrm{nuc}}{n_\textrm{sat}} . \end{aligned}$$

We obtain in SM, $m_\textrm{sat}^*/m=1/(1+\kappa _\textrm{sat})$, and the isospin splitting of the effective mass in NM:

50 $$\begin{aligned} \frac{\varDelta m_\textrm{sat}^*}{m} = \frac{m_n^*}{m_n} - \frac{m_p^*}{m_p} = -\frac{2\kappa _\textrm{sym}}{(1+\kappa _\textrm{sat})^2-\kappa _\textrm{sym}^2} . \end{aligned}$$

The complete list of available models is given with the following instructions: 
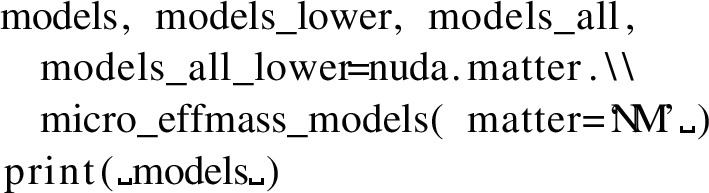


A typical call for given input variables, see above, is: 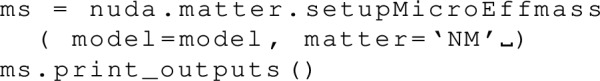 where the variable matter can be ‘NM’ (default), ‘SM’ or ‘AM’. Each calculation can be obtained by fixing the variables model to one of the following values:

model=‘2008-BCS-NM’.

Presented in Sect. 3.7.

model=‘2017-MBPT-NM-GAP-EMG-450-500-N2LO’, ‘2017-MBPT-NM-GAP-EMG-450-500-N3LO’, ‘2017-MBPT-NM-GAP-EMG-450-700-N2LO’, ‘2017-MBPT-NM-GAP-EMG-450-700-N3LO’, ‘2017-MBPT-NM-GAP-EM-500-N2LO’, ‘2017-MBPT-NM-GAP-EM-500-N3LO.

Presented in Sect. 3.7.

model=‘2022-AFDMC-NM’.

Presented in Sect. 3.7.

The object ms contains the effective masses. The entire list of properties is obtained in the following way: 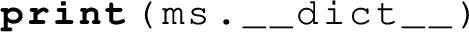


Output quantities are, for instance: ms.nm_effmass and ms.sm_effmass for the nucleon effective masses in NM and SM. ms.am02_effmass_n (ms.am02_effmass_p) for the neutron (proton) effective mass with $\delta =0.2$, and ms.am04_effmass_n (ms.am04_effmass_p) for $\delta =0.4$.

**Fig. 27:**
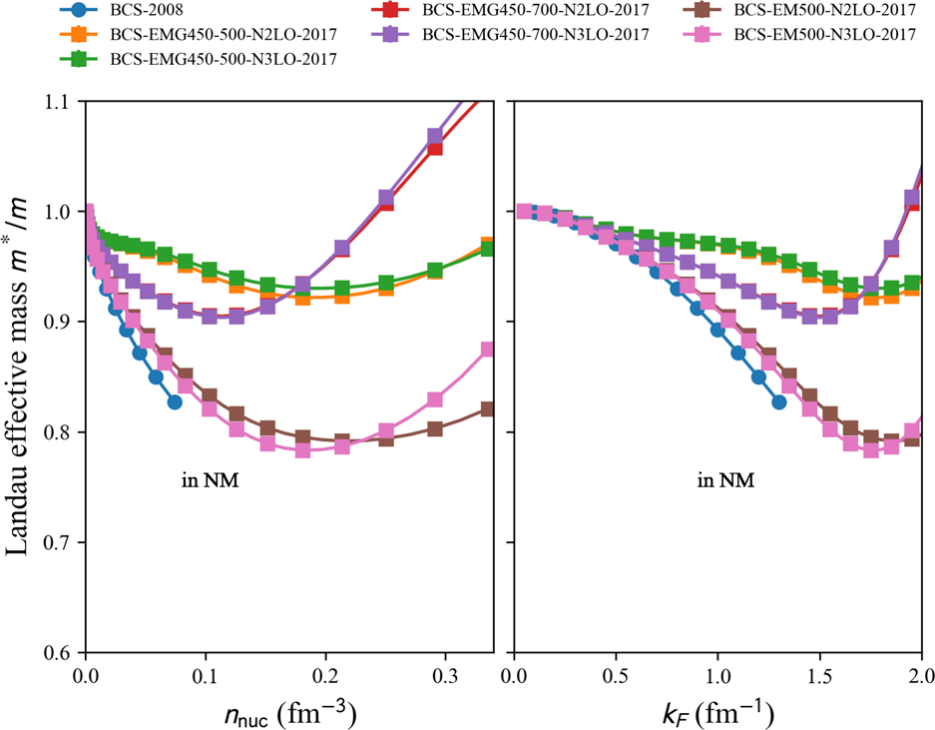
Landau effective mass in NM predicted by the microscopic models available in the nuda toolkit. Figure generated with matter_setupMicro_effmass_plot.py

**Fig. 28:**
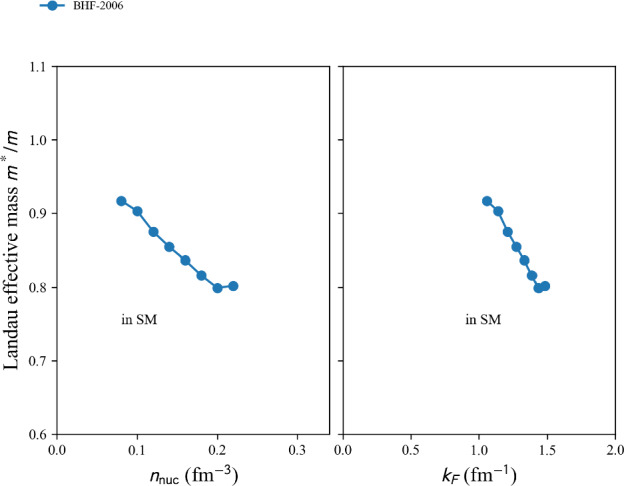
Same as Fig. 27 in SM. Figure generated with matter_setupMicro_effmass_plot.py

**Fig. 29:**
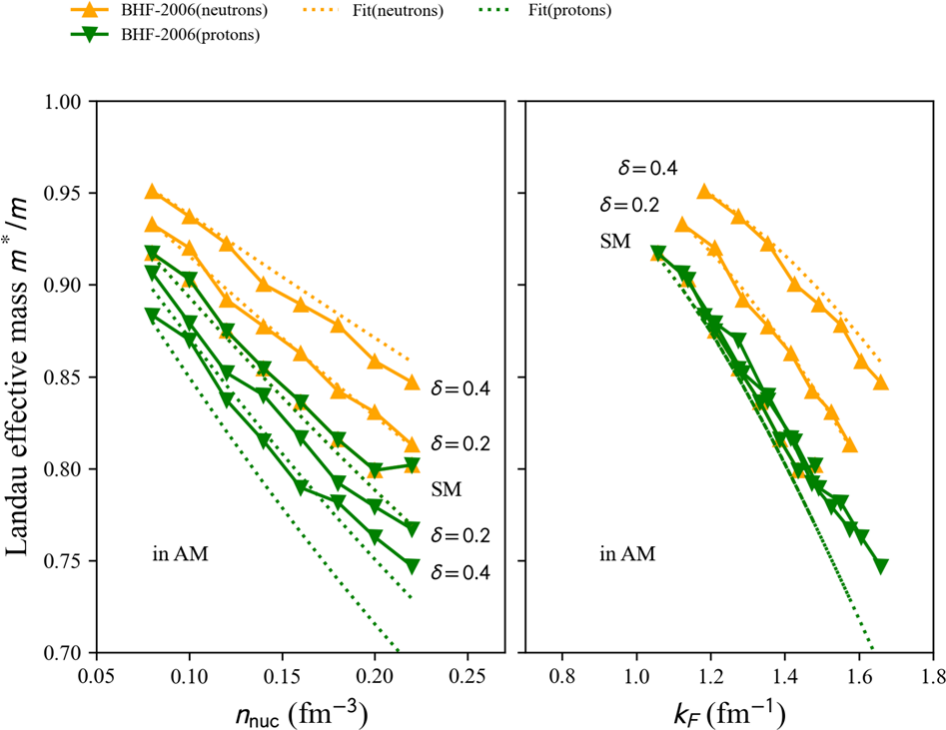
Same as Fig. 27 in AM. Figure generated with matter_setupMicro_effmass_plot.py

The Landau effective masses are shown in Fig. 27 (for NM), Fig. 28 (for SM), and Fig. 29 (for AM) for a set of microscopic models. Based on the BHF results in AM, we suggest the following empirical relation:

51 $$\begin{aligned} \frac{m^*_q}{m_q}(n_\textrm{nuc},\delta ) = \left[ 1+0.2\left( \frac{k_{F\bar{q}}}{k_{F_\textrm{sat}}}\right) ^{3.5}\right] ^{-1} , \end{aligned}$$

where $k_{F\bar{q}}$ is $k_{Fn}$ for $m^*_p/m_p$, and vice-versa. The empirical relation (51) is shown in Fig. 29 for $m^*_n/m_n$ and $m^*_p/m_p$ as a function of the density $n_\text {nuc}$ or Fermi momentum $k_F$ and isospin parameter $\delta$ (dotted lines).

The empirical relation (51) can be obtained from nuda toolkit in the following way: 
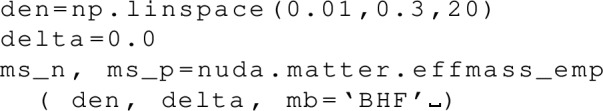


### Landau parameters

The Landau parameters represent the residual nuclear interaction calculated at the Fermi momentum $k_F$ and decomposed into partial waves. They are given as:

52 $$\begin{aligned} V_\text {ph}(k,k^\prime )= &   \frac{1}{N_0} \sum _{L=0}^\infty P_L(\cos \theta ) \Big [ F_L + F^\prime _L \tau _1\cdot \tau _2 \nonumber \\  &   + G_L \sigma _1\cdot \sigma _2 + G_L^\prime (\tau _1\cdot \tau _2)(\sigma _1\cdot \sigma _2) \Big ], \end{aligned}$$

where $P_L$ is the Legendre polynomial for the angular momentum *L*. We have $\vert k\vert =\vert k^\prime \vert =k_F$ and $\cos \theta = k\cdot k^\prime$. The density of states $N_0$ reads $N_0=g m^* k_F /(2\hbar ^2\pi ^2)$.

The complete list of available microscopic predictions for the Landau parameters is given with the following instruction: 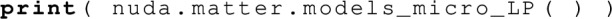 A typical call for given input variables, see above, is: 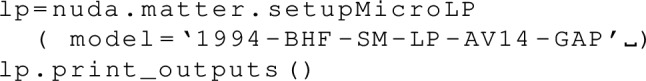 Results can be obtained by fixing the variables model to one of the following values:

model=‘1994-BHF-SM-LP-AV14-GAP’ [101], ‘1994-BHF-SM-LP-AV14-CONT’ [101], ‘1994-BHF-SM-LP-REID-GAP’ [101], ‘1994-BHF-SM-LP-REID-CONT’ [101], ‘1994-BHF-SM-LP-AV14-CONT−0.7’ [101], ‘2006-BHF-SM- Av18’, ‘2006-EBHF-SM-Av18’.

In SM.

model=‘2007-BHF-NM-BONNC’ [102], ‘2006-BHF-SM-Av18’, ‘2006-EBHF-SM-Av18’.

**Fig. 30:**
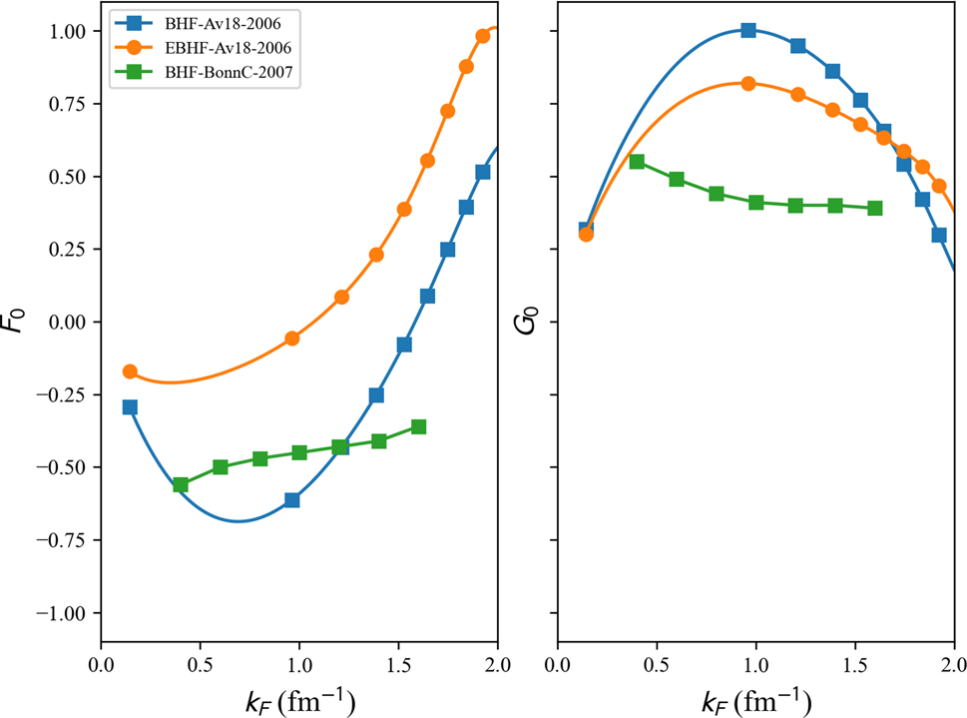
$L=0$ Landau parameters in NM predicted by BHF calculations as given by the models available in the nuda toolkit. Figure generated with matter_setupMicro_LP_plot.py

In NM.

The object lp contains the Landau parameters, some of the attributes listed by the command lp.print_ outputs(). The entire list of properties is obtained in the following way: 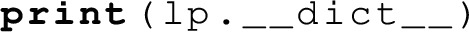


Output quantities are, for instance: lp.sm_LP[cha] [ell] where cha can be ‘F’, ‘G’, ‘Fp’ or ‘Gp’ for the different channels in SM and ell is the angular momentum 0,1, etc. In NM, we have: lp.nm_LP[cha][ell] with cha=‘F’ or ‘G’.

**Fig. 31:**
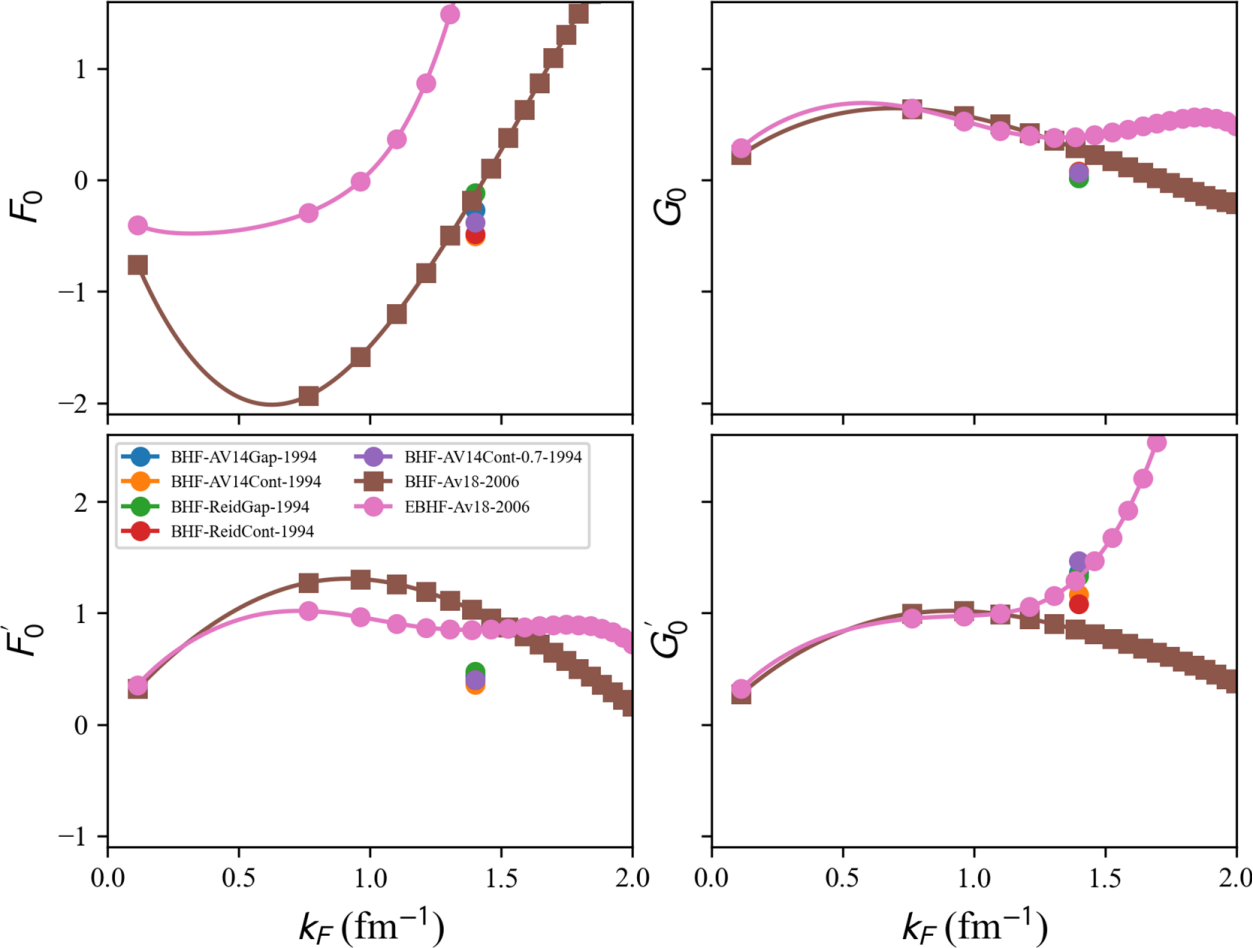
$L=0$ Landau parameters in SM predicted by BHF calculations as given by the models available in the nuda toolkit. Figure generated with matter_setupMicro_LP_plot.py

Predictions for the Landau parameters in NM are shown in Figs. 30 and in  31 for SM. These predictions have been performed for BHF many-body approach, and one prediction is made for an extended BHF approach.

### Experimental constraints from heavy-ion collisions

Heavy-ion collisions are reactions between heavy nuclei, which are described and analyzed using transport simulations, see, for instance, a recent white paper [103]. In the following, we do not describe HIC performed in laboratory experiments and their analysis, but simply provide results in terms of EoS inference performed by various authors.

The complete list of available inferences is given with the following instructions: 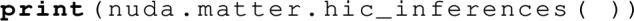

**Fig. 32:**
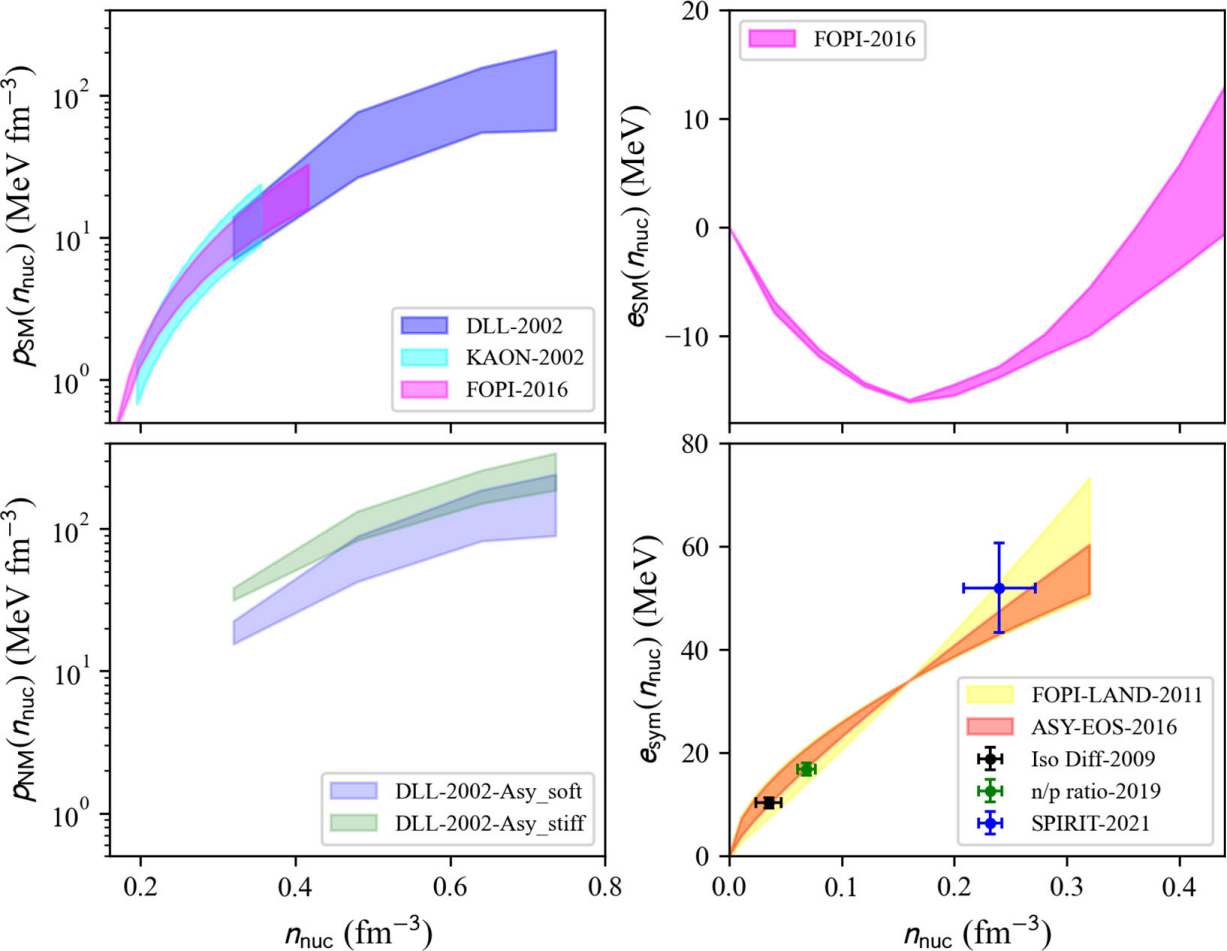
HIC experimental inferences for the pressure and energy per particle (left) in SM (left top) and NM (left bottom), and the internal energy per nucleon (right top) and symmetry energy (right bottom) as a function of the nucleon density for different inferences available in the nuda toolkit. Figure generated with matter_setupHIC_plot.py

Once the variable inference is chosen in the previous list, the call for the EOS inferences can be performed in the following way: 
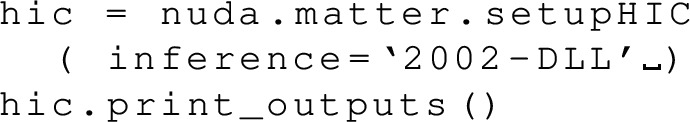
 We have eight different inferences available in the nuda toolkit (’2002-DLL’, ‘2002-KAON’, ‘2016-FOPI’, ‘2009-ISO-DIFF’, ‘2011-FOPI-LAND’, ‘2016-ASY-EOS’, ‘2019-NP-RATIO’, ‘2021-SPIRIT’). These inferences are made for various terms of the EoS: the first three inferences are for SM, and the others are for the symmetry energy. We now detail these inferences:

inference=‘2002-DLL’.

This is the constraint on the pressure obtained from the collective flow of matter [104] for densities going from $2.2n_\textrm{sat}$ up to $4.6n_\textrm{sat}$. The constraints are obtained from the studies on sideward and elliptical anisotropies of protons in Au + Au collisions at energies ranging from low incident energy, $E_\text {inc} \approx$0.15–2 *A*GeV, (at SIS) up to high energies, $E_\text {inc} \approx$2–11 *A*GeV, (at AGS). These constraints rule out strongly repulsive nuclear equations of state ($K_\textrm{sat}> 300$ MeV) from relativistic mean field theory and weakly repulsive equations of state ($K_\textrm{sat}< 167$ MeV) with phase transitions at densities less than three times saturation density $n_\textrm{sat}$. Note, however, that EoS softens by the onset of a phase transition at high density is possible. Results are available for the pressure in SM and NM. Note that in NM, there are two predictions based on two different parametrizations for the symmetry energy (soft and stiff in Fig. 32). Outputs are given in hic.sm_pre_up and hic.sm_pre_lo for the upper and lower boundaries of the pressure in SM, and in hic.sm_pre_so_up (hic.sm_pre_st_up) and hic.sm_pre_so_lo (hic.sm_pre_st_lo) for the soft (stiff) boundary prediction in NM. The densities are given in hic.den_pre. These inferences are shown in Fig. 32.

inference=‘2006-KAON’.

This is the inference for the pressure in SM obtained using the excitation function of $K^+$ multiplicities obtained in Au+Au over C+C systems [105, 106]. The inferences are obtained by comparing data from KaoS experiments for incident energy ranging from 0.8 to 1.5 *A*GeV using QMD and IQMD transport models. The inference extends from $1.2n_{\textrm{sat}}$ to $2.2n_{\textrm{sat}}$ densities [106]. Outputs are given in hic.sm_pre_up and hic.sm_pre_lo for the upper and lower boundaries of the pressure in SM, and the densities are given in hic.den_pre.

inference=‘2016-FOPI’.

This inference is for the internal energy per particle and for the pressure in SM. The data from FOPI experiments on the elliptic flow of protons and light clusters up to mass 3 for the Au+Au collisions are analyzed for incident energy ranging from 0.4 and 1.5 *A*GeV. Inferences for the EoS of compressed SM up to $3n_\textrm{sat}$ are extracted using the transport simulation IQMD [107]. Outputs are given in hic.sm_e2a_int_up (hic.sm_pre_up) and hic.sm_e2a_int_lo (hic.sm_pre_lo) for the upper and lower boundaries of the energy per nucleon (pressure) in SM, and the densities are given in hic.den_e2a (hic.den_pre).

inference=‘2009-ISO-DIFF’.

This inference for the symmetry energy for densities $0.22 \pm 0.07 n_{\textrm{sat}}$ [108] has been obtained from the experimental data on isospin diffusion in collisions of Sn+Sn isotopes at 0.05 *A*GeV incident energy. Constraints are extracted using the ImQMD transport model. Outputs are given in hic.esym and hic.esym_err for the centroid and uncertainty of the symmetry energy at the density hic.den_esym with the uncertainty hic.den_ esym_err.

inference=‘2011-FOPI-LAND’.

The symmetry energy is inferred from the studies of the ratio of the elliptic flow of neutrons and hydrogen isotopes measured for Au+Au collisions at incident energies of 0.4 *A*GeV and using UrQMD transport simulation [109]. This inference goes up $2n_{\textrm{sat}}$ and indicates moderately soft symmetry energy. Outputs are given in hic.esym and hic.esym_err for the centroid and uncertainty of the symmetry energy at the density hic.den_esym with the uncertainty hic.den_esym_err.

inference=‘2016-ASY-EOS’.

The directed and elliptical flows of neutrons and light-charged clusters were measured for Au+Au at 0.4 AGeV by the ASY-EOS experimental campaign [110]. The comparison of this data with the UrQMD transport model helped to get an inference for the symmetry energy up to $2n_\textrm{sat}$. Again, the moderately soft to linear density dependence was seen as consistent with FOPI-LAND results but with reduced uncertainties. Outputs are given in hic.esym_up and hic.esym_lo for the upper and lower boundaries of the symmetry energy at the density hic.den_esym.

inference=‘2019-NP-RATIO’.

The symmetry energy for densities $0.43 \pm 0.05 n_{\textrm{sat}}$ [111] is inferred using the experimental data on spectral ratios of neutron and proton spectra in collisions of Sn+Sn isotopes at 0.12 *A*GeV incident energy and using the ImQMD transport model. Outputs are given in hic.esym and hic.esym_err for the centroid and uncertainty of the symmetry energy at the density hic.den_esym with the uncertainty hic.den_esym_err.

inference=‘2021-SPIRIT’.

The study of the spectral ratios of pions in the Sn+Sn reactions at an incident energy of 270 AMeV [112] provides an inference for the symmetry energy, 52±13 MeV, and for the symmetry pressure $p_\textrm{sym}$, 10.9±8.7 MeV/fm$^3$, at the density of 1.45$\pm 0.2~n_\textrm{sat}$ using the transport model dcQMD. Outputs are given in hic.esym (hic.psym) and hic.esym_err (hic.psym_err) for the centroid and uncertainty of the symmetry energy (symmetry pressure), at the density hic.den_esym with the uncertainty hic.den_esym_err.

**Fig. 33:**
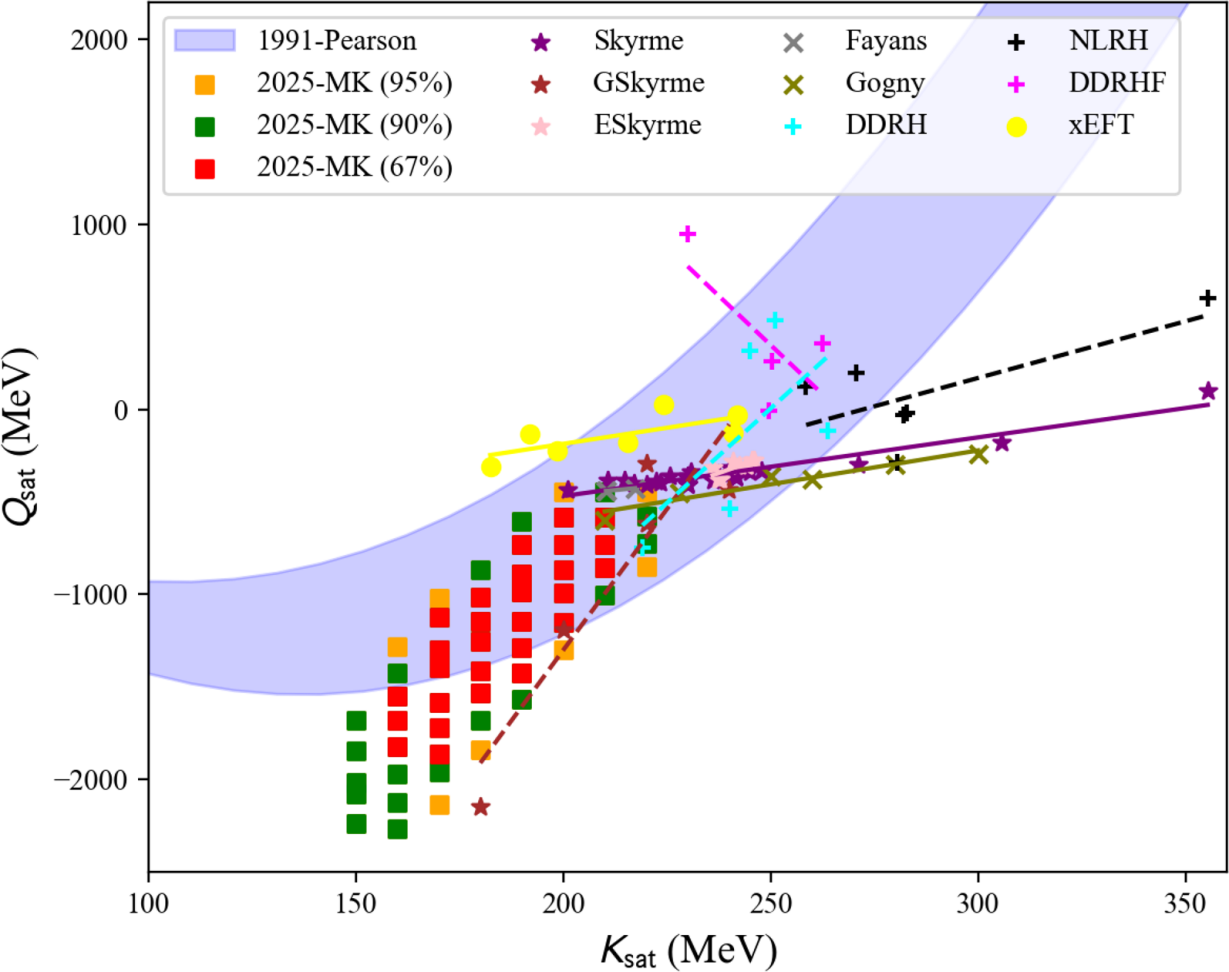
$K_{\textrm{sat}}$-$Q_{\textrm{sat}}$ correlation from the different constraints provided in the nuda toolkit. Figure generated with corr_setupKsatQsat_plot.py

The HIC constraints for the symmetry energy are shown in Fig. 32 (bottom right panel) for the different inferences presented above. It is interesting to see that these different inferences are quite consistent with each other, although they come from different analyses based on different HIC and different transport models.

## Correlation diagrams: the corr module

We now introduce the model corr of the nuda toolkit that is aimed at producing correlations among NEPs originating from experimental or theoretical constraints.

### The correlation between $K_\textrm{sat}- Q_\textrm{sat}$

The values for the NEP $K_\textrm{sat}$ and $Q_\textrm{sat}$ are very instructive for the inference of the density dependence of the EoS in SM around saturation density. While $K_\textrm{sat}$ is determined by the analysis of the isoscalar Giant Monopole Resonance (ISGMR) in heavy nuclei, providing $K_\textrm{sat}=240\pm 20$ MeV [113], following the procedure suggested by Blaizot [114], the value for $Q_\textrm{sat}$ remains quite unknown. It was, furthermore, observed that most EDFs induce strong correlations between $K_\textrm{sat}$ and $Q_\textrm{sat}$, see Fig. 33 and Refs. [97, 98]. It is also known that the accurate determination of $K_\textrm{sat}$ is related to a narrow exploration of $Q_\textrm{sat}$ [99]. So one may wonder if the small uncertainty in the extraction of $K_\textrm{sat}$, $\pm 20$ MeV, is underestimated [115]. The nuda toolkit provides a method to easily extract and represent the correlation between $K_\textrm{sat}$ and $Q_\textrm{sat}$, see Fig. 33 and the following.

The complete list of constraints is given with the following instructions: 
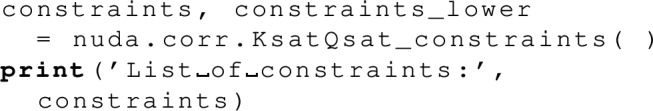


A typical call for given input variable constraint, see above, is: 
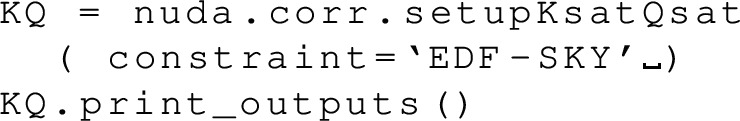
 Results can be obtained by fixing the variable constraint to one of the following values:

constraint=‘1991-Pearson’.

Experimental band for $K_\textrm{sat}$ and $Q_\textrm{sat}$ given in Ref. [116] and obtained from Ref. [117]. Outputs are given in KQ.Ksat_ band and the lower and upper boundary of the contour are given in KQ.Qsat_band_lo and KQ.Qsat_band_up.

constraint=‘2025-MK-67’, ‘2025-MK-90’, ‘2025-MK-95’.

The 67%, 90%, and 95% confidence levels for a set of extended Skyrme interactions reproducing the binding energy, charge radii, and isoscalar giant monopole resonance in $^{120}$Sn and 208Pb, more details in Ref. [115]. Outputs are given in KQ.Ksat and KQ.Qsat.

constraint=‘EDF-SKY’, ‘EDF-GSKY’, ‘EDF-ESKY’, ‘EDF-Gogny’, ‘EDF-xEFT’, ‘EDF-NLRH’, ‘EDF-DDRH’, ‘EDF-DDRHF’.

Values for $K_\textrm{sat}$ and $Q_\textrm{sat}$ predicted by a set of nuclear interactions [97, 98, 115]. For each one of these constraints, outputs are given in KQ.Ksat and KQ.Qsat, and a linear regression is provided in KQ.Ksat_lin and KQ.Qsat_lin.

We show constraints for the $K_\textrm{sat}$-$Q_\textrm{sat}$ correlation plot in Fig. 33. Some models are very correlated (solid lines) while others are more loosely correlated (dashed lines). However, the region of $Q_\textrm{sat}$ explored by the different models is narrow. For each model, the value for $Q_\textrm{sat}$ is quite constrained by the value of $K_\textrm{sat}$. The experimental domain for Ref. [118] is shown in blue, and the extended Skyrme models obtained in Ref. [115] are also shown, with different colors reflecting the confidence level as shown in the legend. Based on this correlation plot, it is argued in Ref. [115] that the experimental uncertainty in $K_\textrm{sat}$ is underestimated. While the microscopic approach suggested in Ref. [114] is the most accurate way to extract the value of $K_\textrm{sat}$ from the analysis of experimental data for the ISGMR, the use of models presenting strong correlations among parameters is to be taken with caution. More flexible microscopic models exploring the parameter space widely for $K_\textrm{sat}$ and $Q_\textrm{sat}$ may be more appropriate to better represent the experimental uncertainty.

### The correlation between $E_{\textrm{sym},2} - L_{\textrm{sym},2}$

In the following, we differentiate the correlation originating from measurements in finite nuclei, which probe the symmetry energy around SM, from the one originating from dense matter and neutron star observation, which probe the very asymmetric behavior of the symmetry energy ($\beta$-equilibrium). Since the first one (finite nuclei) constrains the quadratic contribution to the symmetry energy, we will represent the constraint in terms of $E_{\textrm{sym},2}$ and $L_{\textrm{sym},2}$, while the second one (neutron star) provide constraints for the symmetry energy in very asymmetric matter and NEP $E_{\textrm{sym},2}$ and $L_{\textrm{sym},2}$.

The correlation between $E_{\textrm{sym},2}$ and $L_{\textrm{sym},2}$ has been investigated by many different approaches over the last decade, see for instance Refs. [119, 120] for more details. Note that since most of these constraints are extracted from analyses of experimental nuclear data, they concern the quadratic contribution of the symmetry energy $e_{\textrm{sym},2}$. The nuda toolkit provides a large list of these approaches. The complete list of available constraints is given with the following instruction: 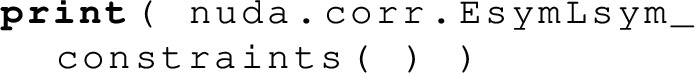


Once the variable constraint is chosen in the previous list, the call for the correlation induced by this constraint in the $E_{\textrm{sym},2}$-$L_{\textrm{sym},2}$ correlation plot can be performed in the following way: 
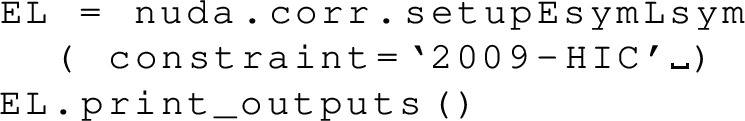


Results can be obtained by fixing the variable constraint to one of the following values:

constraint=‘2009-HIC’.

Constraints inferred from isospin diffusion in heavy ion collisions (HICs) [108]. Outputs are given in EL.Esym and EL.Lsym with EL.Lsym_err.

constraint=‘2010-RNP’.

Constraints deduced from the analysis of neutron-skin thickness $\varDelta r_{np}$(Sn) in Sn isotopes [121]. Outputs are given in EL.Esym and EL.Lsym with EL.Lsym_err.

constraint=‘2012-FRDM’.

Constraint from the finite-range droplet mass model (FRDM) calculations [122]. Outputs are given in EL.Esym and EL.Lsym with EL.Lsym_err.

constraint=‘2014-IAS’.

Constraint deduced from the analysis of the excitation energy of the isobaric analog state (IAS) based on Skyrme–Hartree–Fock calculations [123]. Outputs are given in EL.Esym and EL.Lsym with EL.Lsym_err.

constraint=‘2014-IAS+Rnp’.

Combination of the IAS constraint and neutron skin $\varDelta r_{np}$ in $^{208}$Pb [123]. Outputs are given in EL.Esym and EL.Lsym with EL.Lsym_err.

constraint=‘2015-POL-68NI’, ‘2015-POL-120SN’, ‘2015-POL-208PB’.

Constraints on the electric dipole polarizability of $^{208}$Pb, $^{120}$Sn and $^{68}$Ni [124]. Outputs are given in EL.Lsym and EL.Esym with EL.Esym_err.

constraint=‘2021-PREXII-Reed’ [125], ‘2021-PREXII-Reinhard’ [126], ‘2023-PREXII+CREX-Zhang’ [127].

**Fig. 34:**
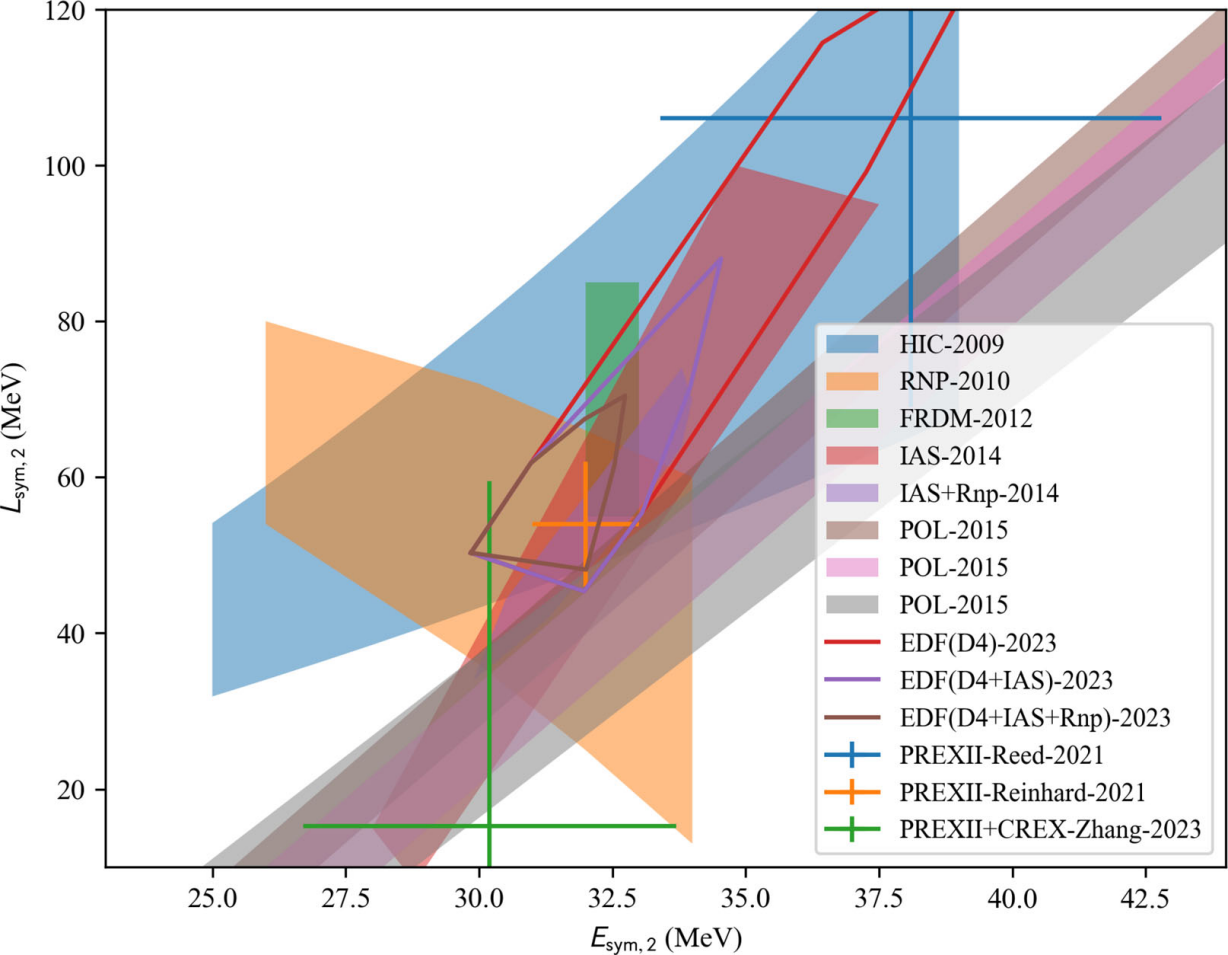
$E_{\textrm{sym},2}$-$L_{\textrm{sym},2}$ correlation from the different constraints provided in the nuda toolkit. Figure generated with corr_setupEsym2Lsym2_plot.py

**Fig. 35:**
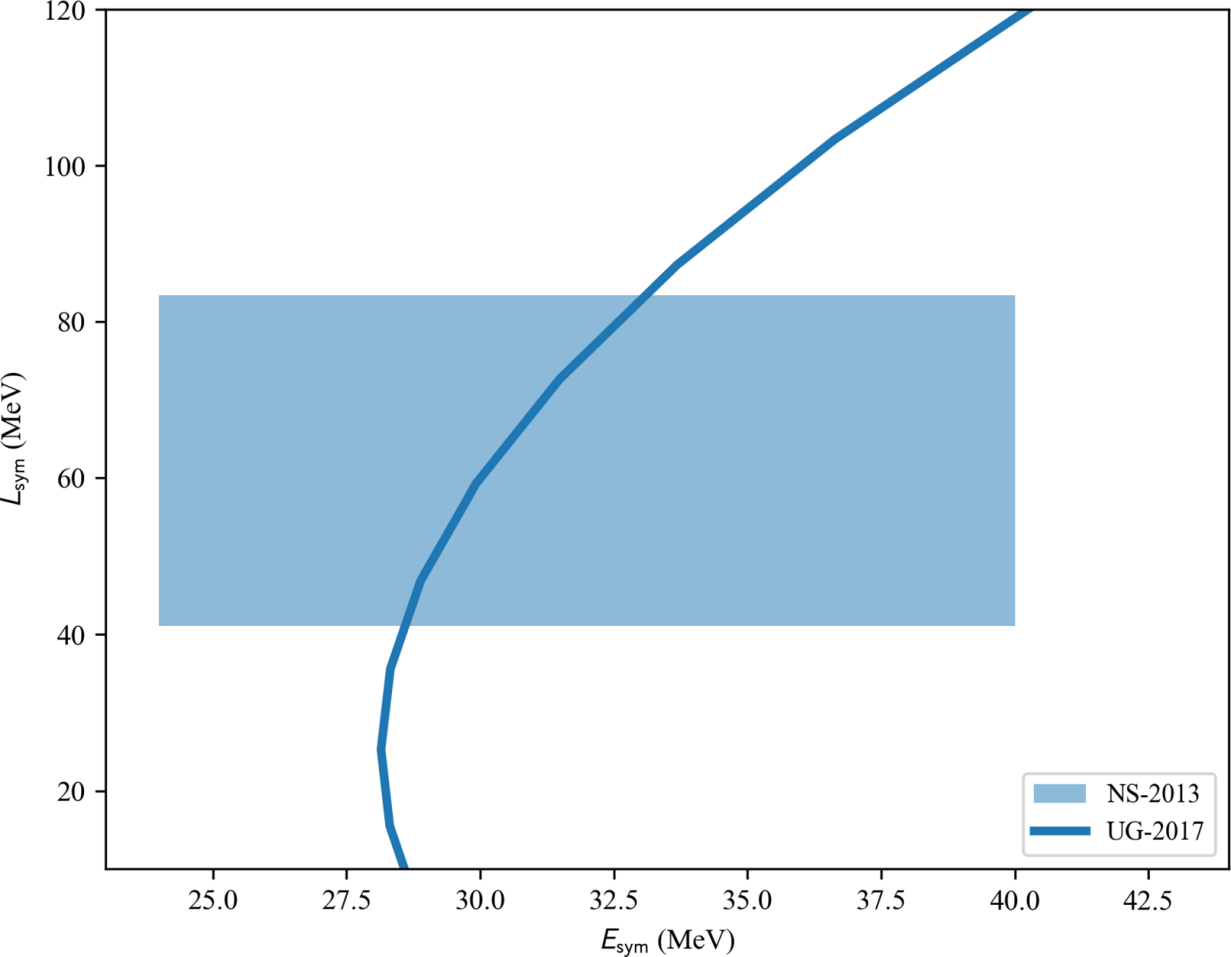
$E_\textrm{sym}$-$L_\textrm{sym}$ correlation from the different constraints provided in the nuda toolkit. Figure generated with corr_setupEsymLsym_plot.py

PREX-II [128] and CREX [129] are parity-violating electron scattering (PVES) experiments that have been performed at JLab. There are big differences between the original analysis by Reed et al. [125] suggesting $E_{\textrm{sym},2}=38.1\pm 4.7$ MeV, $L_{\textrm{sym},2}=106\pm 37$ MeV, and the one by Reinhard et al. [126], which also includes the constraint from electric dipole polarizability, suggesting $E_{\textrm{sym},2}=32\pm 1$ MeV, $L_{\textrm{sym},2}=54\pm 8$ MeV. Another analysis by Zhang and Chen [127] combining PREX-II and CREX using a Bayesian inference find a very low centroid for $L_{\textrm{sym},2}$ ($E_{\textrm{sym},2}=30.2^{+3.0}_{-4.1}$ MeV, $L_{\textrm{sym},2}=15.3^{+41.5}_{-46.8}$ MeV). It has indeed been pointed out that the results of PREX-II and CREX are in disagreement [130, 131]. Outputs are given in EL.Esym with EL.Esym_err and EL.Lsym with EL.Lsym_err.

constraint=‘2023-EDF-D4’, ‘2023-EDF-D4-IAS’, ‘2023-EDF-D4-IAS-Rnp’.

The capacity of a total of 415 Skyrme and relativistic energy density functional (EDF) models is analyzed in Ref. [132]. The constraint ‘2023-EDF-D4’ requires that EDF models describe $N=Z$ and $N\ne Z$ spherical nuclei with the same accuracy (binding energy, charge radii and ISGMR). The constraint ‘2023-EDF-D4-IAS’ implies that the isobaric analog state (IAS) is reproduced as well, i.e., the contour for $e_\textrm{sym}(n_\textrm{nuc})$ suggested in Ref. [123]. Finally, the constraint ‘2023-EDF-D4-IAS-Rnp’ implies that the neutron radius is reproduced on top of previous constraints, i.e., the contour for $e_\textrm{sym}(n_\textrm{nuc})$ suggested in Ref. [123].

All these constraints are shown in Fig. 34. This figure is similar to previous ones, see for instance Refs. [119, 120]. The constraints imposed by the different analyses of PREX-II and CREX parity-violating electron scattering (PVES) experiments are shown as crosses in Fig. 34. There is almost no overlap between these crosses, but it should be remembered that the uncertainties are given for only 67% confidence interval. It is however interesting to note that there is a small region in the correlation plot $E_{\textrm{sym},2}$-$L_{\textrm{sym},2}$, coinciding with the one suggested by the constraint ‘2021-PREXII-Reinhard’ [126], which satisfy all constraints, except for the constraints related to electric dipole polarisability, which shifts $E_{\textrm{sym},2}$ towards slightly larger values and/or $L_{\textrm{sym},2}$ towards lower values.

### The correlation between $E_{\textrm{sym}} - L_{\textrm{sym}}$

As discussed in the previous section, the constraints from finite nuclei from those from dense matter and neutron stars are differentiated. In this section, we focus on the constraints from neutron stars.

The variable constraint can be ‘2013-NS’ or ‘2017-UG’ as detailed in the following:

constraint=‘2013-NS’.

The constraint on $L_\textrm{sym}$ has been obtained from a Bayesian analysis of mass and radius observations of NSs by considering the 95% confidence level for $L_{\textrm{sym}}$ [133]. Outputs are given in EL.Esym and EL.Lsym with EL.Lsym_err.

constraint=‘2017-UG’.

The analysis of the unitary gas predictions for the symmetry energy parameters [134] permits the values to the right of the curve. Outputs are given in EL.Esym and EL.Lsym.

These two constraints are shown in Fig. 35. There is still a large domain in $E_\textrm{sym}$ and $L_\textrm{sym}$ compatible with these constraints. It would therefore be interesting to investigate further the constraints on $E_\textrm{sym}$ and $L_\textrm{sym}$ imposed by the observation data from neutron stars.

### The symmetry energy: $e_{\textrm{sym},2}(n_\textrm{nuc})$

**Fig. 36:**
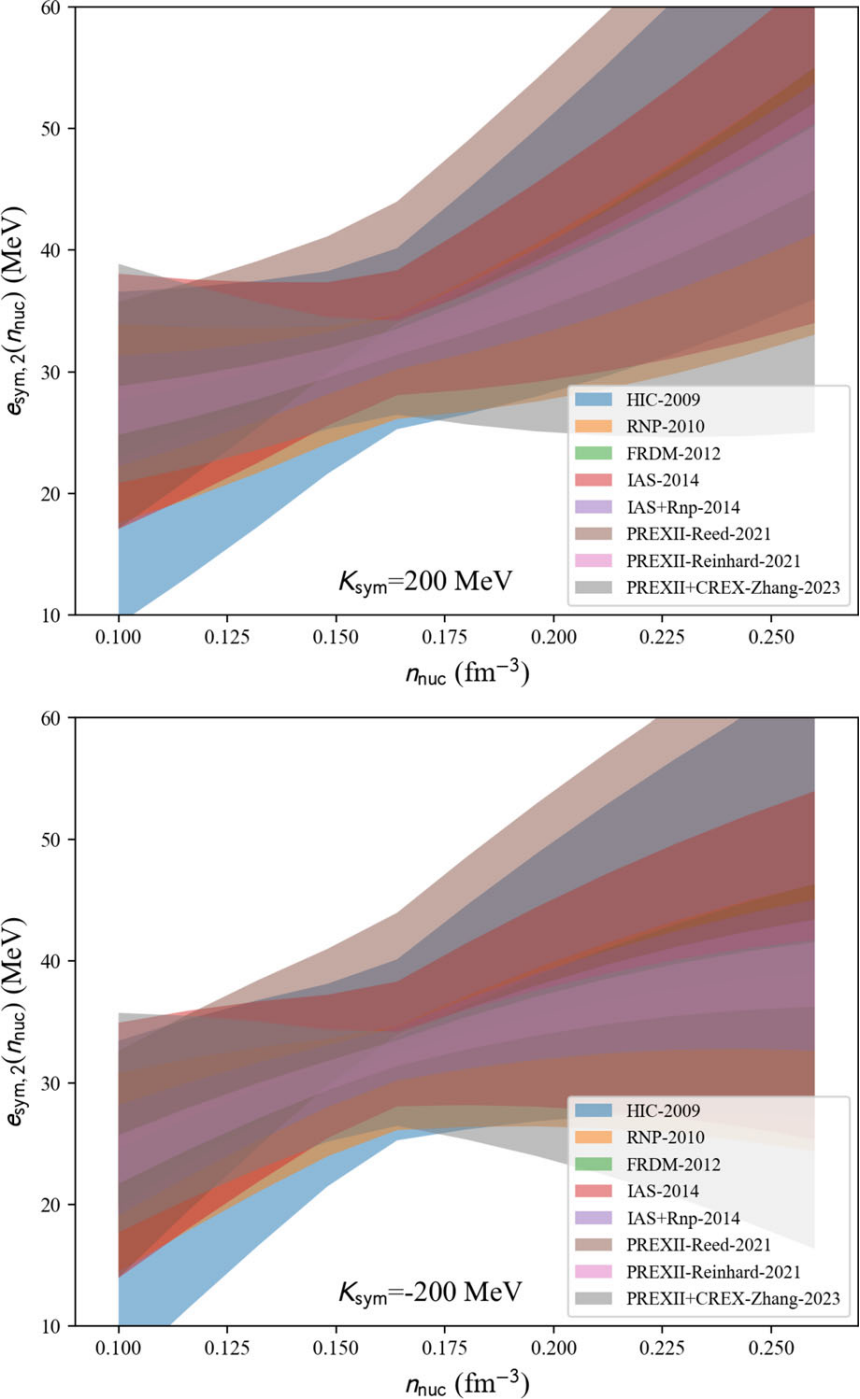
The density dependent $e_{\textrm{sym}}(n_\textrm{nuc})$ from the different constraints provided in the nuda toolkit and for three arbitrary choices of $K_\textrm{sym}$=200 (top) and -200 MeV (bottom). Figure generated with corr_setupEsymDen_plot.py

From the constraints on $E_{\textrm{sym},2}$ and $L_{\textrm{sym},2}$ provided by the different constraints previously listed, one can reconstruct the quadratic contribution to the symmetry energy $e_{\textrm{sym},2}(n_\textrm{nuc})$ around $n_\textrm{sat}$ as:

53 $$\begin{aligned} e_{\textrm{sym},2}(n_\textrm{nuc})= &   E_{\textrm{sym},2} + L_{\textrm{sym},2}\frac{n_\textrm{nuc}-n_\textrm{sat}}{3n_\textrm{sat}} \nonumber \\  &   +\frac{1}{2} K_{\textrm{sym},2}\left( \frac{n_\textrm{nuc}-n_\textrm{sat}}{3n_\textrm{sat}}\right) ^2 + \cdots \end{aligned}$$

The call for the density-dependent symmetry energy for a given constraint can be done in the following way: 
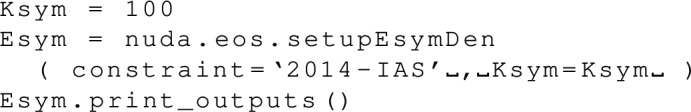
 where the variable constraint takes the same input values as the class corr.setupEsymLsym. They are listed in the previous subsection.

The value for the NEP $K_{\textrm{sym},2}$ is largely unknown, see the discussion in Sect. 3.12. From Sect. 3.12, it can, however, be safely varied from $K_{\textrm{sym},2}$=-200, to 200 MeV, as suggested as well in Ref. [44]. For these two values for $K_\textrm{sym}$, it is interesting to explore the prediction for the density dependence of the symmetry energy $e_{\textrm{sym},2}(n_\textrm{nuc})$ allowed by the previously presented constraints, see Fig. 36.

## Data from finite nuclei: the nuc module

In this section, we describe how the nuda toolkit can be employed to obtain some finite nuclei properties, e.g., binding energies, charge radii, ISGMR energies, etc. It is then possible, for instance, to use these experimental data for finite nuclei to calibrate models for the crust of neutron stars.

### Experimental binding energies over the nuclear chart

To obtain experimental binding energies for finite nuclei, a mass table should first be selected. The complete list of available experimental mass tables in nuda toolkit can be obtained in the following way: 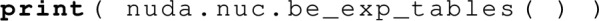 In the present version of the nuda toolkit, there is only one mass table available: ‘AME’. The toolkit is however ready to read more mass tables.

The ‘AME’ mass table has been released several times, with more and more nuclei. The different versions of the table are associated with the year of their release. The complete list of available versions of the experimental mass tables is given with the following instructions: 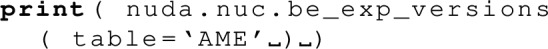 For the table ‘AME’, the nuda toolkit provide the following versions: ‘2012’ [135], ‘2016’ [136], ‘2020’ [137]

Once the variables table and version are chosen, the call for the experimental nuclear chart can be performed in the following way: 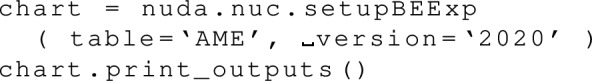


We now provide more details about the experimental nuclear tables available in the nuda toolkit. Here are the present options available:

table=‘AME’.

version= ‘2012’ [135], ‘2016’ [136], ‘2020’ [137].

The content of several output attributes is provided with the command chart.print_outputs(), and their complete list can be obtained as: 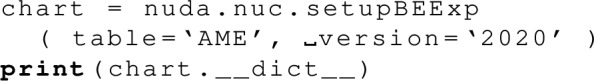 We now provide more details on these outputs. They are provided as a Numpy array containing nuclear properties provided by the selected mass table. The nuda toolkit gives chart.nucA for the mass number $A=N+Z$, chart.nucZ for the nuclear charge *Z*, chart.nucN for the neutron number *N*, chart.nucI for the isospin parameter *I* defined as $I=(n-Z)/A$, and the binding energy (experimental uncertainty) is in chart.nucBE (chart.nucBE_err). Additionally, the nuclear symbol is given in chart.nucSymb, the interpolation flag is stored in chart.flagInterp (it is set to ‘n’ if the nucleus is not interpolated (true measurement) and ‘y’ if the nucleus is not measured but provided from the interpolation provided by the authors of the mass table), chart.flagI is identical to the variable i in the original mass table: i=0 for nuclei measured in their ground-state, the attribute chart.nucStbl reflect if the nucleus is stable (’y’) or unstable (’n’), the measured half-time in seconds is given in chart.nucHT, the discovery year is in chart.nucYear, and the maximum charge in the table is in chart.Zmax.

**Fig. 37:**
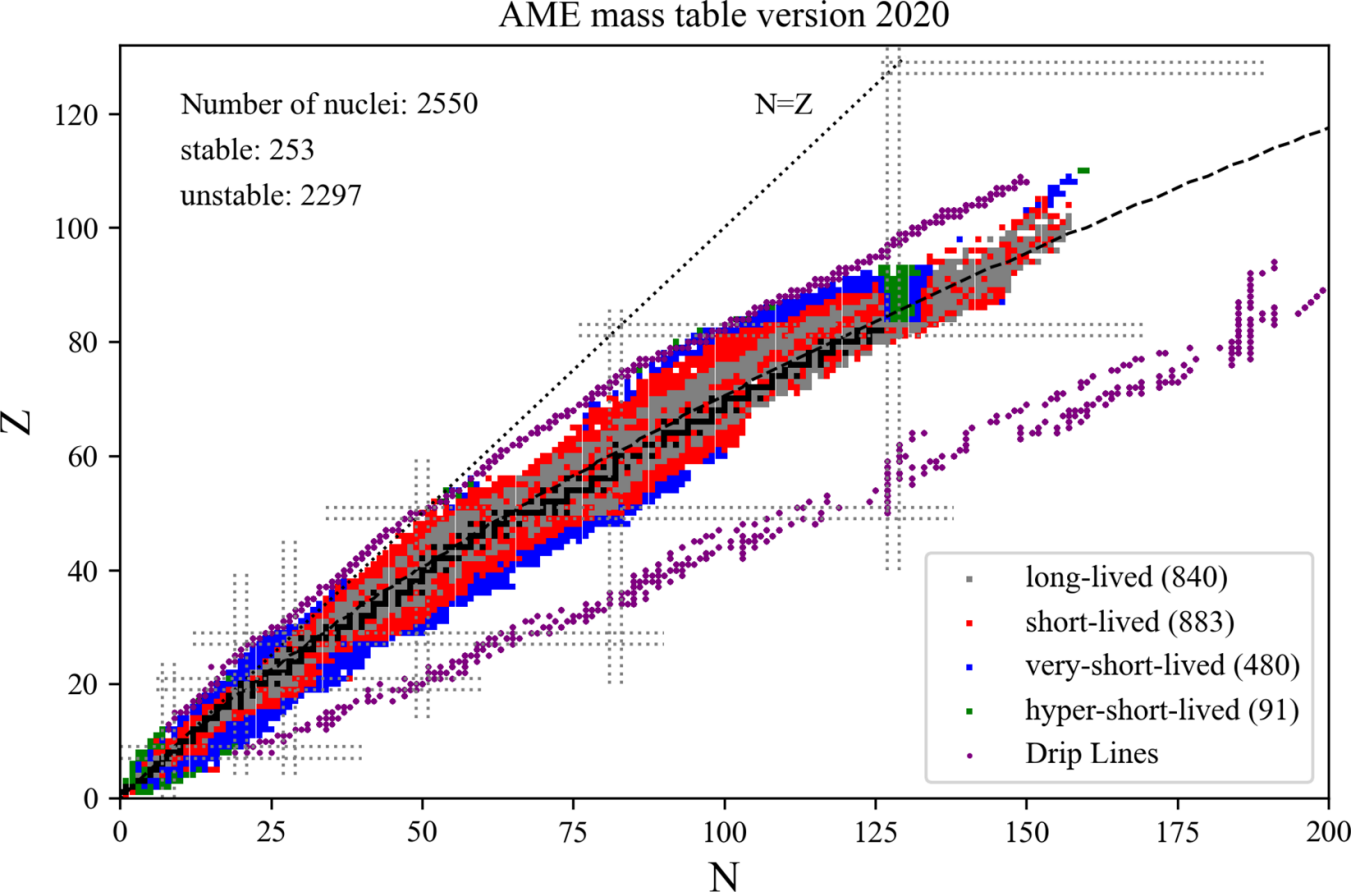
Nuclear chart from AME 2020 nuclear chart available in the nuda toolkit. The driplines (in magenta) are calculated for the following set of models: ‘2013-HFB22’, ‘2013-HFB23’, ‘2013-HFB24’, ‘2013-HFB25’, ‘2013-HFB26’, ‘2021-BSkG1’, ‘2022-BSkG2’, ‘2023-BSkG3’. See text for more details. Figure generated with nuc_setupBEExp_chart_lt_plot.py

The method select can be applied to the object chart to select a smaller list of nuclei respecting some requirements. The command is the following: 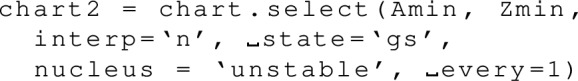 where all input variables are optional. The input Amin and Zmin allows the user to select nuclei above the limit they impose (default value is 0, Amin and Zmin can be fixed independently from each other), the input variable interp select nuclei that are not interpolated (’n’) or only interpolated nuclei (’y’), the input variable state can be ‘gs’ to select nuclei measured in their ground-state (default), the input variable nucleus can be ‘unstable’ (default), ‘stable’, ‘longlive’, ‘shortlive’ or ‘veryshortlive’, as described below, and finally the input variable every select nuclei by steps defined by every (default is 1, no selection).

The attributes of the object chart2 are the same as the ones associated with the object chart. They have a name with sel_ distinguishing them. With obvious notations, see above, the attributes are: chart2.sel_nucA, chart2.sel_nucZ, chart2.sel_nucN, chart2. sel_nucI, chart2.sel_nucBE, chart2.sel_BE_ err, chart2.sel_nucSymb, chart2.sel_ flagInterp, chart2.sel_flagI, chart2.sel_ nucHT, chart2.sel_nucYear, chart2.sel_Zmax.

The nuclear chart for the 2020 AME mass table [137] contains 2550 nuclei (with 253 stable nuclei) in their ground state. It is shown in Fig. 37, where we display the nuclear chart as a function of the number of neutrons (*x*-axis) and protons (*y*-axis). The dotted lines associated with shell closure are obtained from the function plot_shells(axs) for axs a figure created by matplotlib. Nuclei are colored according to their half-life: stable nuclei (black) have a half-life greater than Earth’s age and define the so-called valley of stability (in dashed line, the fit is provided by the function stable_fit_Z(Zmin,Zmax) or stable_fit_N(Nmin,Nmax) defined as

54 $$\begin{aligned} N(Z)= &   Z(1+6\,10^{-3} Z) \, \end{aligned}$$

55 $$\begin{aligned} Z(N)= &   N ( 1 - 3.8\,10^{-3} N + 9\,10^{-6} N^2 ) , \end{aligned}$$

and providing as output N and Z), long-lived nuclei (grey) are nuclei for which the half-life is larger than 1-day, short-lived nuclei (red) are the ones that live more than 1-second, very-short-lived nuclei (green) are the ones that live more than 1-millisecond, and the other are hyper-short-lived nuclei (green). Theoretical drip lines (purple) are also shown for several EDFs, see the caption of Fig. 37 for the list of EDFs, and Sect. 5.2 for the use of theoretical models.

The 1-day frontier between long-lived and short-lived nuclei is a typical time separating nuclear chemistry and nuclear physics, and the 1-second frontier between short-lived and very short-lived nuclei represents the frontier above which nuclei shall be studied online, just after their production. Note that 1 ms is the time for a nucleus to travel 3 km at the speed of 1% of *c* (no relativistic time dilation). Very few nuclei in the mass table live shorter than 1 ms (green color). The 1 ms frontier is the new frontier of research for modern facilities exploring rare isotopes, e.g., RIKEN, FRIB, FAIR, SPIRAL2, etc. In the future, more and more hyper-short-lived nuclei will be produced and characterized on the way to the drip lines. The four domains shown in Fig. 37 are therefore reflecting the progress in technology: using chemistry to identify artificial (long-lived) isotopes, using physics detectors to identify rare (short-lived) isotopes, on-line facilities dedicated to (very-short-lived) exotic nuclei, and finally, (hyper-short-lived) nuclei requiring secondary beams to be produced and compact facilities for their detection. They are at the frontier of research.

Note that there is a large empty space between the nuclei experimentally produced on Earth and the predicted drip lines (magenta dots), except for light nuclei and for some neutron-poor nuclei. The dispersion in green points reflects an uncertainty in the model prediction for the location of the drip lines. Note also that Fig. 37 is one of the many representations possible with the nuda toolkit. It is not the purpose of the present work to list them all, and we simply illustrate the capacities of nuda.

**Fig. 38:**
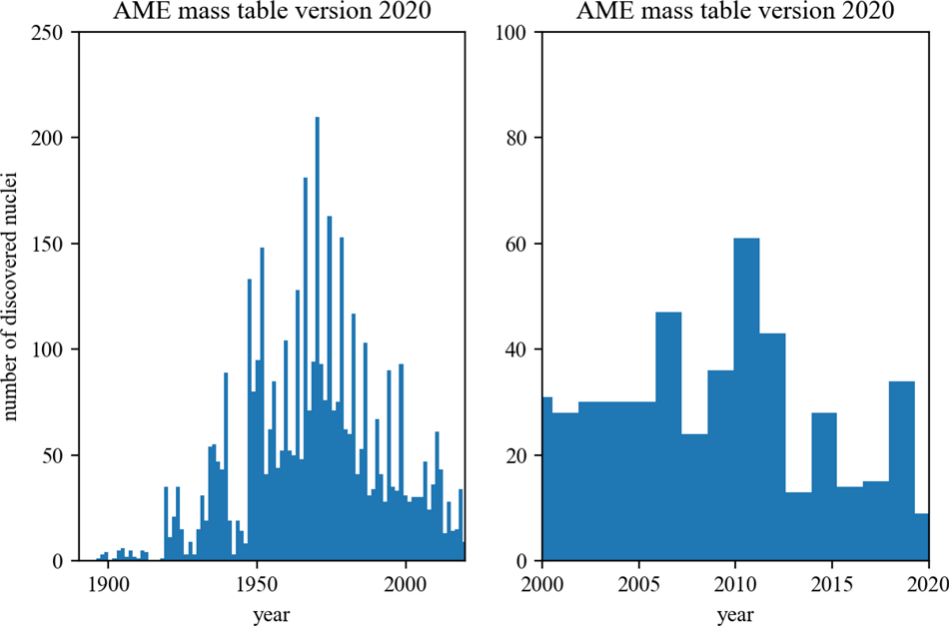
Number of nuclei function of their discovery year. Figure generated with nuc_setupBEExp_year_plot.py

Another illustration of the capacities of nuda is shown in Fig. 38, where experimental nuclei are distributed according to the year of their discovery. On the left is shown the distribution since the first one in 1897, and on the right is a zoom-in for the recent period between 2000 and 2020. The peak of discoveries is located in the 60ies, when a large number of facilities across the world contributed to the blooming of discoveries. More recently, the cost to produce new nuclei is such (all modern facilities produce secondary beams) that there a fewer exotic nuclei factories.

Figure 38 is obtained by using the method select_year() that is called in the following way: 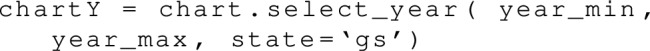 where year_min and year_max are the boundaries for the discovery years and state is ‘gs’ for ground-state nuclei (default). The attributes of chartY are the same as the ones of chart2.

Once nuclei are selected in chart2, selecting nuclei in their ground state for instance, there are two additional methods that can be applied to the object chart2, isotopes(Zref) and isotones(Nref), to fix the boundaries for a isotopic or isotonic chain. These methods are called in the following way: 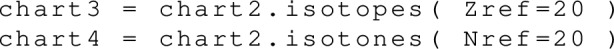 The object chart3 (chart4) contains the boundary of the isotopic (isotonic) chains for Zref (Nref). For the present example, chart3 contains the boundaries chart3.itp_Nmin and chart3.itp_Nmax for calcium isotopes existing in the selected nuclear chart while chart4 contains boundaries chart4.itn_Zmin and chart4.itn_Zmax for $N=20$ isotones.

### Nuclear chart from theoretical models

Predictions for nuclear binding energies from a set of theoretical models are also available in nuda toolkit. The complete list of available theoretical tables is given with the following instructions: 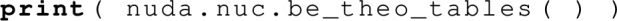 among which a value for the variable table can be chosen. The call for the theoretical nuclear energies can be performed in the following way: 
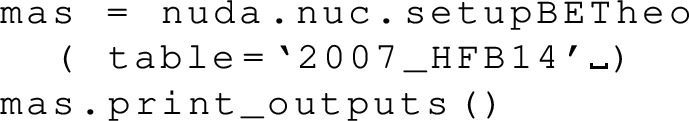
 where we have chosen the theoretical mass model ‘2007_HFB14’. The input variable table can be selected among the following values.

table=‘1988-GK’.

This empirical calculation is based on known masses of neighboring nuclei from transverse Garvey–Kelson (GK) mass relations for approximately 5800 atomic masses are reported in Ref. [138]. While this table is given in the nuda toolkit, we will not show results since they are quite poor.

table=‘1988-MJ’.

This empirical calculation is based on an inhomogeneous partial difference equation, incorporating shell-dependent symmetry energy expressions, Coulomb energy corrections, and higher-order isospin contributions, for approximately 4385 nuclear masses are reported in Ref. [139].

table=‘1995-DZ’.

This phenomenological calculation is based on the shell model, incorporating renormalized monopole and multipole interactions with empirical fits to known masses, for approximately 1751 nuclear masses with $N, Z \ge 8$ are reported in Ref. [140].

table=‘1995-ETFSI’.

This semi-classical calculation is based on the extended Thomas-Fermi plus Strutinsky integral method, including shell and BCS pairing corrections with a Skyrme force. Approximately 1680 nuclear masses with $36 \le A \le 300$ are reported in Ref. [141].

table=‘1995-FRDM’.

This macroscopic-microscopic calculation is based on the finite-range droplet macroscopic model (FRDM) and the folded-Yukawa single-particle microscopic model. The results of [142] present 8979 nuclei ranging from $Z, N \ge 8$ to $A = 339$.

table=‘2005-KTUY’.

This Macroscopic-microscopic mass model is based on a gross macroscopic term and a microscopic shell correction derived from spherical single-particle potentials. The model includes an improved even-odd term to account for pairing effects. The results of [143] present 9437 nuclei ranging from $Z, N \ge 2$ to $Z \le 130$, $N \le 200$.

table=‘2007-HFB14’.

This microscopic mass model is based on a nuclear EDF of Skyrme type. It solves HFB equations on a harmonic oscillator basis and imposes axial symmetry. HFB14 [54] is fitted to experimental masses and fission data by adjusting a vibrational term in the phenomenological collective correction.

table=‘2010-HFB21’.

This microscopic mass model is based on a nuclear EDF of extended-Skyrme type. It solves HFB equations on a harmonic oscillator basis and imposes axial symmetry. HFB21 [144] is fitted to experimental masses and realistic calculations for neutron matter energy in homogeneous matter.

table=‘2010-WS*’.

This macroscopic-microscopic mass model is based on a liquid-drop macroscopic term and a Strutinsky shell correction. The model WS$^*$ [145] incorporates a mirror nuclei constraint to improve isospin symmetry and refines the Coulomb energy term.

table=‘2011-WS3’.

The macroscopic-microscopic mass model WS3 [146] based on a liquid-drop macroscopic term and a Strutinsky shell correction was further refined by incorporating mirror nuclei constraints, Wigner-like effects and additional residual corrections.

table=‘2013-HFB22’, ‘2013-HFB23’, ‘2013-HFB24’, ‘2013-HFB25’, ‘2013-HFB26’.

Extended-Skyrme Hartree–Fock–Bogoliubov (HFB) mass models are reported in Ref. [81]. The models are fitted to experimental masses AME12, realistic calculations for neutron matter energy in homogeneous matter while varying the symmetry coefficient $E_\textrm{sym}$ from 29 to 32 MeV.

table=‘2021-BSkG1’.

This microscopic mass model is based on a nuclear EDF of Skyrme type. The BSkGs models solve HFB calculations on a three-dimensional coordinate-space representation. BSkG1 [58] incorporates for the first time the possibility of triaxial ground-state deformation on global nuclear structure models.

table=‘2022-BSkG2’.

This microscopic mass model is based on an EDF of Skyrme type. In addition to the features of BSkG1, BSkG2 [59] allows for the effects of time-reversal symmetry breaking. This enables the model to access the spin and current densities in the ground states of odd-mass and odd-odd nuclei. BSkG2 also includes information on fission barrier heights of actinide nuclei in the parameter adjustment.

table=‘2023-BSkG3’.

This microscopic mass model is based on an EDF of extended-Skyrme type. The improvements of BSkG3 [83] for BSkG1 and BSkG2 are: (i) break reflection symmetry, allowing for both triaxial and octupole-deformed ground states at the same time; (ii) produce stiff EoS, ensuring the model can accommodate the existence of heavy pulsars; (iii) replace the phenomenological pairing interaction of previous models by a more microscopically grounded interaction designed to match the $^1 S_0$ pairing gaps in NM and SM deduced from ab-initio calculations.

table=‘2025-BSkG4’.

This microscopic mass model is based on an EDF of extended-Skyrme type. In addition to the features of BSkG3, BSkG4 [84] offers an improved treatment of $^1 S_0$ nucleon pairing gaps in asymmetric nuclear matter, inspired by advanced many-body calculations.

**Fig. 39:**
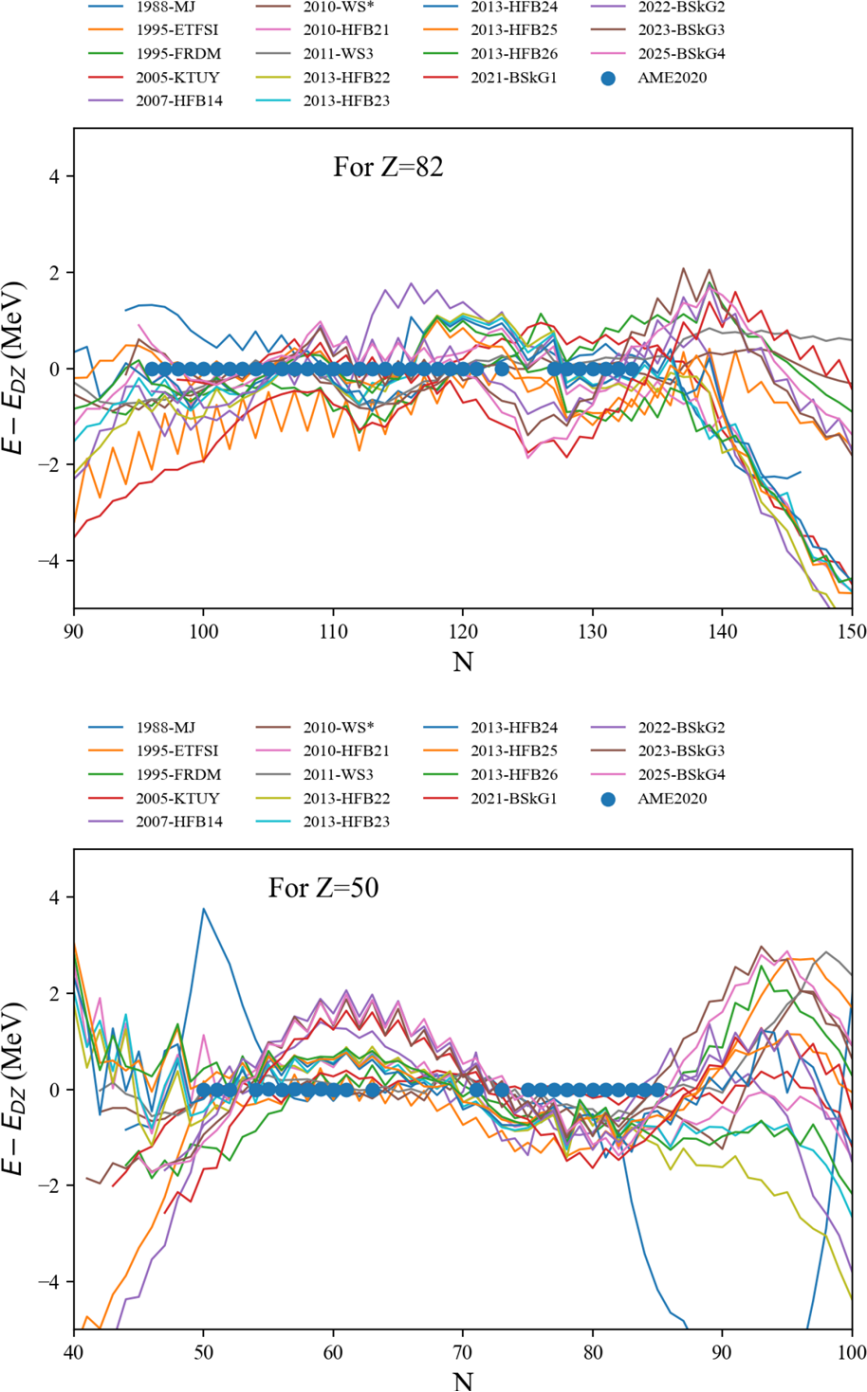
Differences between binding energies predicted by different models with respect to the one predicted by Duflo-Zuker (’1995-DZ’) for $Z=82$ (top) and $Z=50$ (bottom). The experimental measurements reported in AME 2020 mass table are also shown. Figure generated with nuc_setupBETheo_diff_plot.py

The outputs provided by these theoretical tables are the following: mas.nucA, mas.nucZ, mas.nucN, mas.nucI, mas.nucBE, and mas.Zmax with obvious notations (same as for the experimental mass table detailed in Sect. 5.1). The attribute mas.nucBE2A contains the binding energy per nucleon.

An illustration of the use of the theoretical mass models is shown in Fig. 39 for Sn and Pb isotopic chains. The difference between the theoretical mass model and the Duflo-Zuker [140] one is shown in Fig. 39. The difference between the experimental data from AME 2020 [137] and the Duflo-Zuker mass model is also shown in blue dots. These dots are very flat and close to zero, although they are not identical to zero. However, this remark legitimates taking the Duflo-Zuker mass model as a reference one, even where there are no experimental data. Note that the most significant differences occur for neutron number for which there are no experimental data (low and high neutron numbers). All mass models have parameters adjusted to experimental nuclear masses, therefore, the extreme isospin asymmetries are unconstrained by nuclear experimental data.

The comparison between different models is obtained from mas.diff function in the following way: 
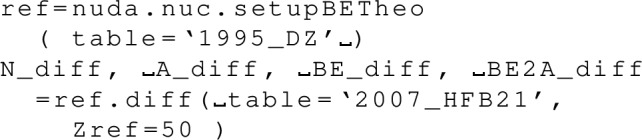
 where the object ref contains the reference mass table, the variable table contains the name of the mass table to compare with, and finally, the variable Zref contains the reference charge of the isotopic chain.

### Two-neutron and two-proton separation energies

The experimental mass tables as well as the theoretical ones can be employed to extract the two-neutron separation energies, $S_{2n}$, and the two-proton one, $S_{2p}$, defined, respectively, as:

56 $$\begin{aligned} S_{2n}= &   E(Z,N)-E(Z,N-2) , \end{aligned}$$

57 $$\begin{aligned} S_{2p}= &   E(Z,N)-E(Z-2,N) . \end{aligned}$$

From the theoretical mass models, $S_{2n}$ and $S_{2p}$ can be obtained in the following way: 
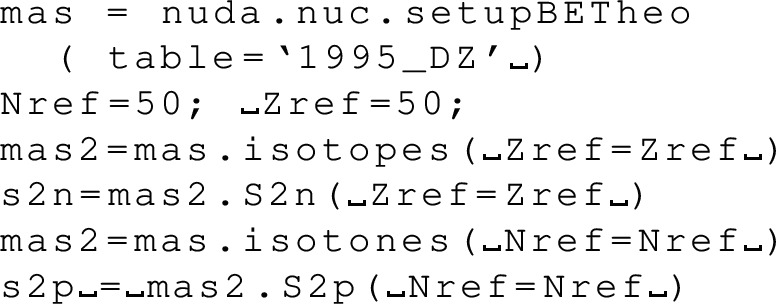
 From the experimental mass tables, they can be obtained in the following way: 
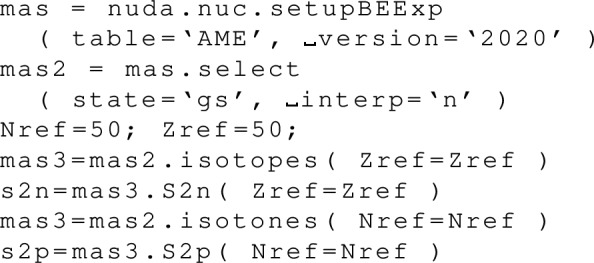
 There is an additional selection of the nuclei in their ground state and an exclusion of the interpolation that shall be applied to the experimental mass table.

The attributes of the objects are Numpy arrays: s2n.s2n_N and s2n.s2n_E for $S_{2n}$ and s2p.s2p_Z and s2p.s2p_E for $S_{2p}$.

**Fig. 40:**
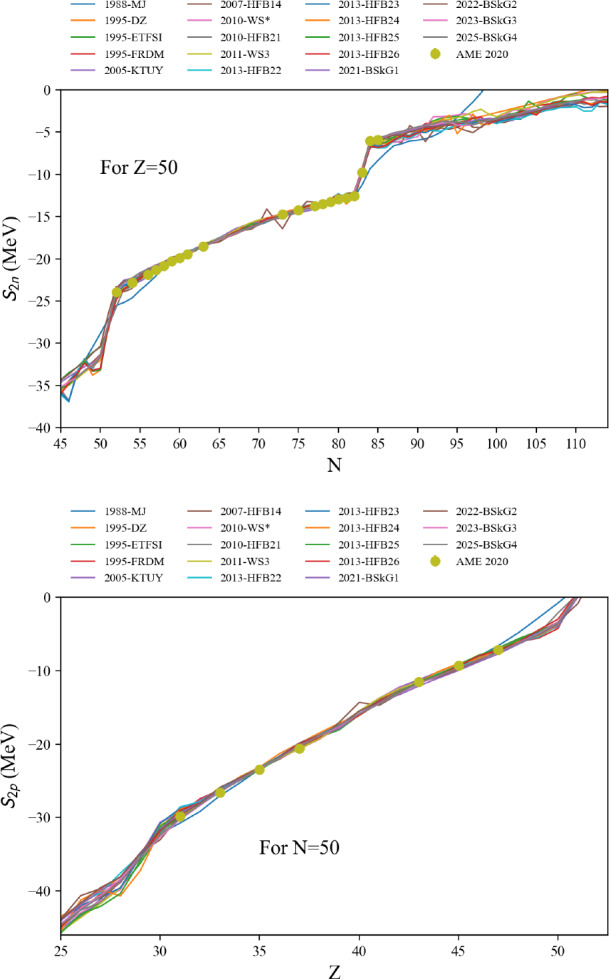
Two-neutron $S_{2n}$ and two-proton $S_{2p}$ separation energies for $Z=50$ (top) and $N=50$ (bottom). The experimental measurements reported in AME 2020 are also shown. Figure generated with nuc_setupBETheo_S2n_plot.py and nuc_setupBETheo_S2p_plot.py

A comparison of model predictions and experimental data for the $S_{2n}$ ($S_{2p}$) is shown Fig. 40 on the top (bottom) for $Z=50$ ($N=50$). The $S_{2n}$ ($S_{2p}$) are negative for bound systems and change their sign at the drip lines. The plots for $S_{2n}$ and $S_{2p}$ exhibit a smooth trend, except close to shell closure. There are changes of slopes for typical magic numbers: 28, 50, 82. Note however the difference between $S_{2n}$ and $S_{2p}$: shell effects are more pronounced for $S_{2n}$ compared to $S_{2p}$. This difference arises because neutron shell effects more directly influence the total energy, while the influence of proton shells is smoothed by Coulomb repulsion and proton-neutron interactions.

### Odd-even mass staggering

There is another interesting quantity which can be extracted from the nuclear mass: The odd-even mass staggering. It can be obtained from the 3-point formula for isotopes,

58 $$\begin{aligned} \varDelta _{3n} = \frac{(-1)^N}{2}\Big ( E(Z,N-1)-2E(Z,N)+E(Z,N+1) \Big ) , \end{aligned}$$

and, for isotones,

59 $$\begin{aligned} \varDelta _{3p} = \frac{(-1)^Z}{2}\Big ( E(Z-1,N)-2E(Z,N)+E(Z+1,N) \Big ) . \end{aligned}$$

From the theoretical mass models, $\varDelta _{3n}$ and $\varDelta _{3p}$ can be obtained in the following way: 
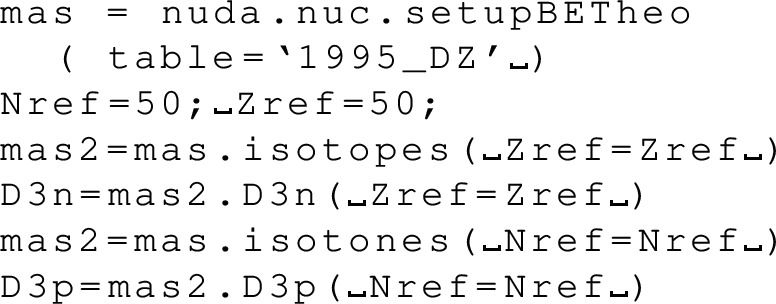
 From the experimental mass tables, they can be obtained in the following way: 
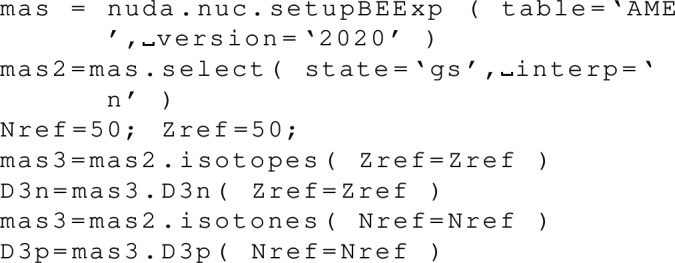
 Similarly to the calculation of the separation energies, there is an additional selection of the nuclei in their ground state and an exclusion of the interpolation that shall be applied to the experimental mass table.

The attributes of the objects are Numpy arrays: D3n.D3n _N_even and D3n.D3n_E_even for $\varDelta _{3n}$ for even *N* and D3p.D3p_Z_even and D3p.D3p_E_even for $\varDelta _{3p}$ for even *Z*. For odd *N* and *Z*, simply replace even by odd in the name of the attributes.

The smooth *Z* and *N* behavior of the odd-even mass staggering is employed to define empirical expressions. These empirical relations are encoded in the nuda toolkit in the following way: 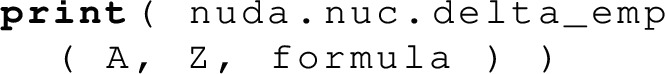 where the variable formula can be one of the following choice:

formula=‘classic’.

Classical formula from Ref. [147]:

60 $$\begin{aligned} \varDelta (A) = 12 A^{-1/2}\hbox { MeV} . \end{aligned}$$

formula=‘Vogel’.

By studying the OEMS for $50<Z<82$ and $82<N<126$, the following empirical formula best reproduces nuclear data for isotopic and isotonic chains, see Ref. [148],

61 $$\begin{aligned} \varDelta (A,Z) = \left( 7.2 -44 [(N-Z)/A]^2 \right) A^{-1/3}\hbox { MeV} . \end{aligned}$$

**Fig. 41:**
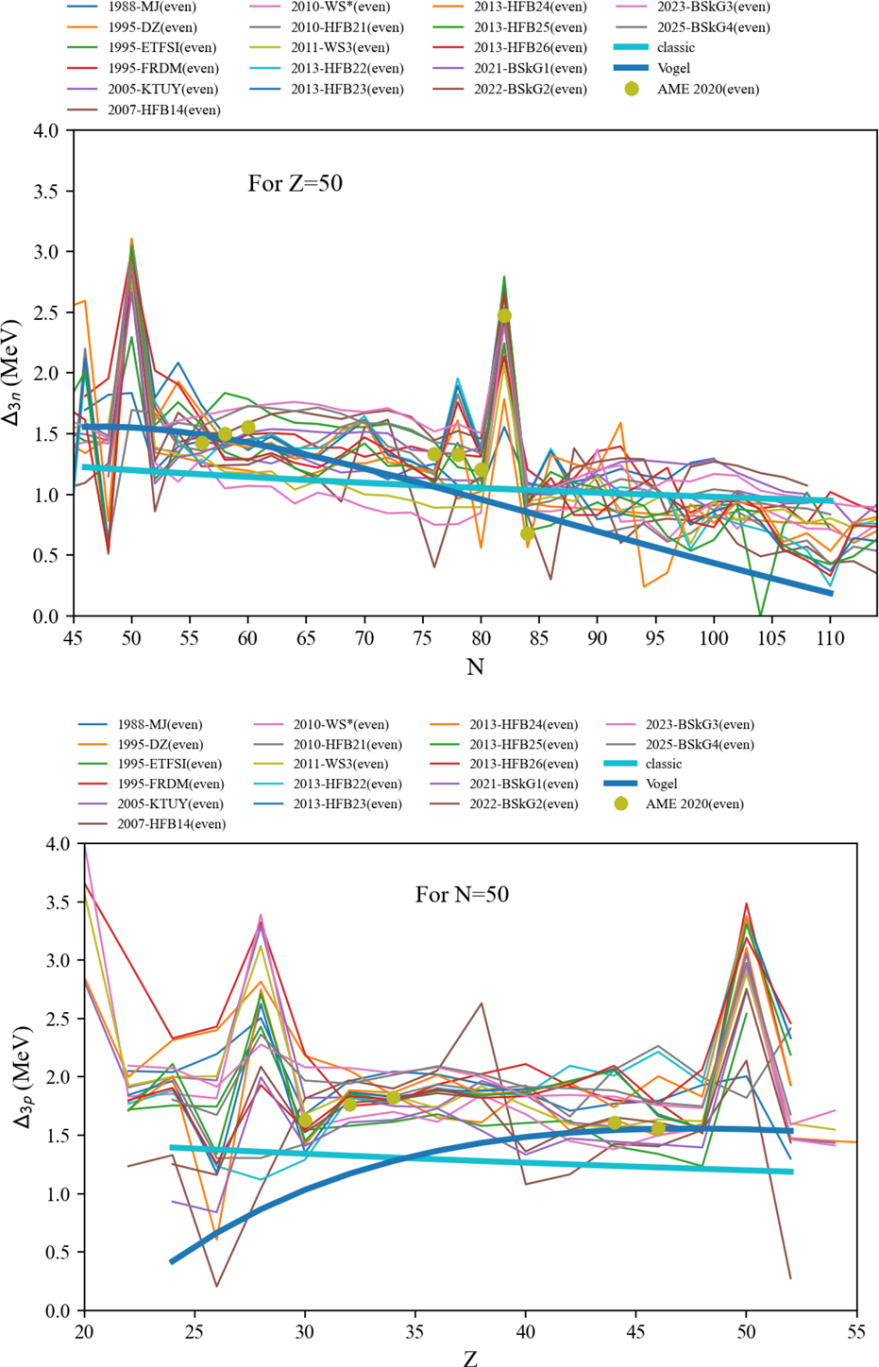
Odd-even mass staggering $\varDelta _{3n}$ for $Z=50$ (top) and $\varDelta _{3p}$ for $N=50$ (bottom). The experimental measurements reported in AME 2020 mass table are also shown. Figure generated with nuc_setupBETheo_D3p_plot.py

**Fig. 42:**
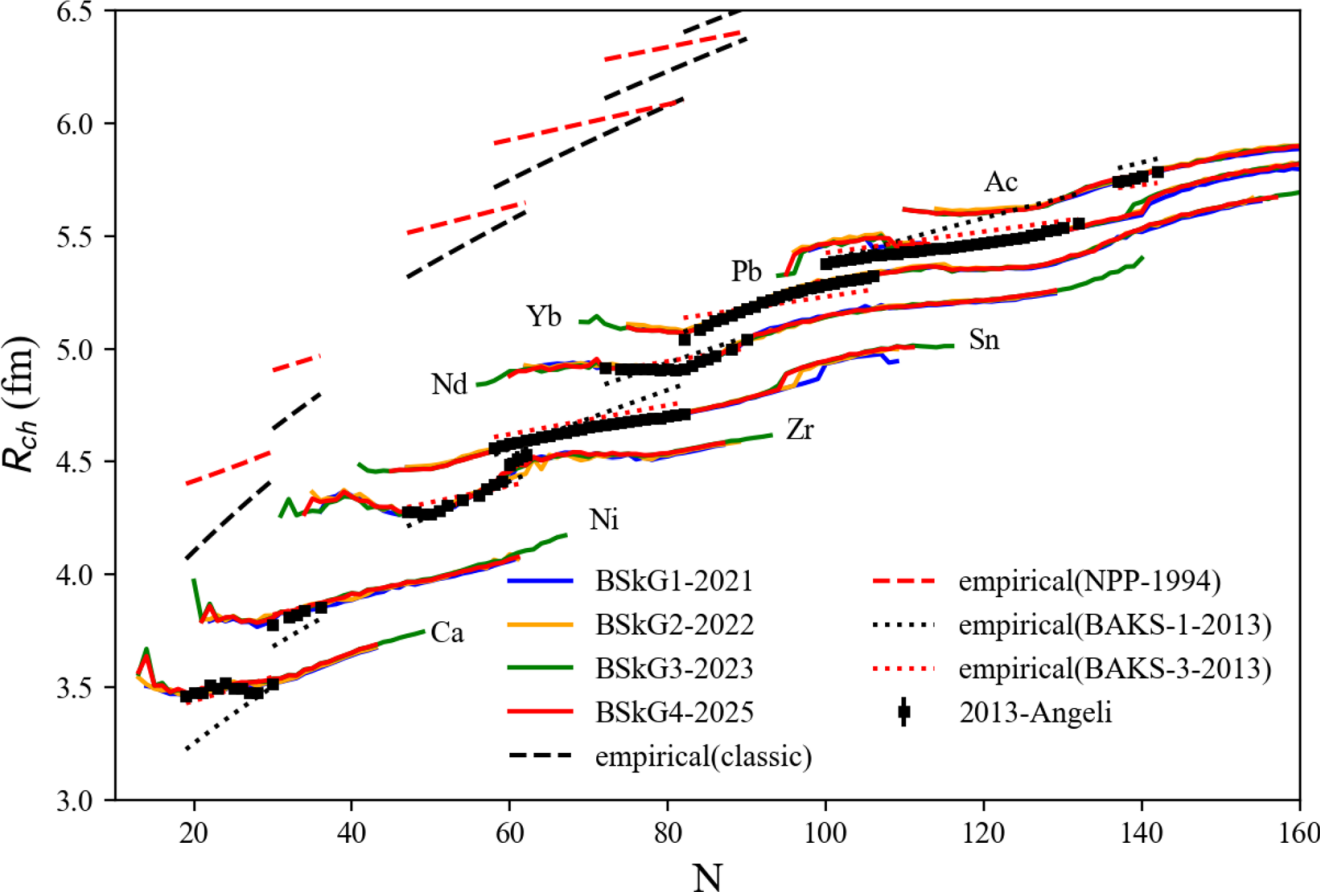
Charge radii for Ca, Ni, Zr, Sn, Nd, Yb, Pb and Ac isotopes and for the models available in the nuda toolkit. Figure generated with nuc_setupRchTheo_plot.py

The odd-even mass staggering are shown in Fig. 41 for a set of theoretical models and for the experimental data (dots) for even *N* or *Z*. The Sn isotopic chain (top) and $N=50$ isotonic chain (bottom) are shown. The empirical formulae are shown with thick solid lines. The peaks at shell closure are visible for magic numbers. The dispersion among theoretical models is larger than the experimental uncertainties. The empirical formulae are adjusted on the isotopic chains and reproduce rather well the smooth behavior of the data shown on the top panel of Fig. 41. For the isotonic chain shown on the bottom panel of Fig. 41, the empirical formulae are slightly below the experimental data.

### Nuclear charge radii

Nuclear charge radii play a key role in determining nuclear shell structure and provide direct information on the Coulomb energy in nuclei. It impacts nuclear astrophysics and imposes strong constraints on the saturation properties of nuclear interactions [149]. The latest compilation of nuclear charge radii for stable and unstable nuclei is the one by Angeli and Marinova in 2013 [150]. Since then, laser spectroscopy has been employed to determine the charge radii of exotic nuclei, but no new table yet exists.

The complete list of available experimental tables is given with the following instructions: 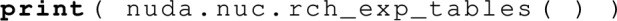 This instruction provides only one output since there is only one mass table available for the moment: ‘2013-Angeli’ [150]. Once the variable table is chosen, the call for the experimental table can be performed in the following way: 
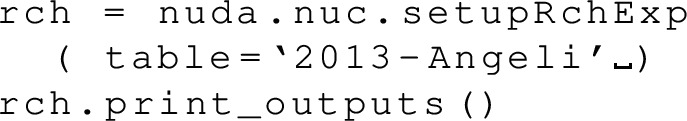


We now provide more details about the experimental nuclear tables available in the nuda toolkit.

table=‘2013-ANGELI’ [150].

The object rch contains the whole experimental table. In the following, we are interested in a subset of data, for the isotopic chain, for instance. The charge radii for an isotopic chain identified by the selection of a nuclear charge in the variable Zref are obtained in the following way: 
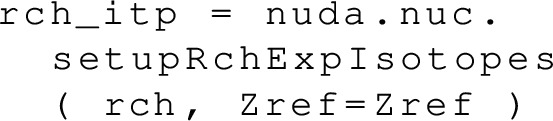
 which attributes are the following Numpy arrays: rch_itp.A, rch_itp.Z, rch_itp.N, rch_itp.Rch, and rch_itp.Rch_err.

Some of the mass models given in the nuda toolkit also provide the charge radii that they predict. The list of theoretical mass models providing charge radii is provided by the following command: 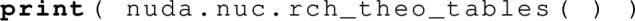 One can choose the variable table among the following options, ‘2021-BSkG1’, ‘2022-BSkG2’, ‘2023-BSkG3’, ‘2025-BSkG4’, which are described in Sect. 5.2. The theoretical table is loaded in the following way: 
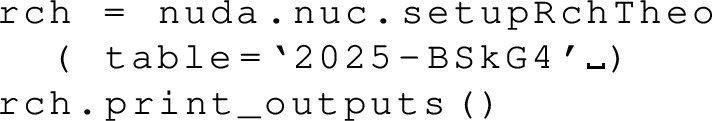
 for the choice table = ‘2025-BSkG4’, and the results for a given isotopic chain is obtained as, 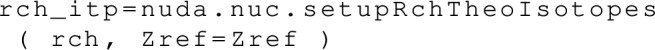 The attributes are the same as the ones which have been given for the experimental table: rch_itp.A, rch_itp.Z, rch_itp.N, rch_itp.Rch, and rch_itp.Rch_err.

**Table 5:** Parameters of Eq. (63) for different choices for the variable formula

formula	$r_0$ fm	*b*	*c*	References
’classic’	1.2	0	0	[147]
’1994-NPP’	1.24	0.191	1.646	[151]
’2013-BAKS-1’	0.951	0	0	[152]
’2013-BAKS-2’	0.996	0.278	0	[152]
’2013-BAKS-3’	0.966	0.182	1.652	[152]

In Fig. 42 we show a comparison between the experimental table and the theoretical predictions for various isotopic chains: Ca, Ni, Zr, Sn, Nd, Yb, Pb, and Ac. We also show some empirical formulae for nuclear charge radii that have been proposed in the literature. They can be obtained from the following function: 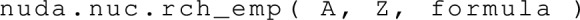 where the variable formula can be one of the following choice:

formula=‘classic’.

Classical formula from Ref. [147]:

62 $$\begin{aligned} r_{ch}(A) = 1.2 A^{1/3}\hbox { fm} . \end{aligned}$$

formula=‘1994-NPP’ [151], ‘2013-BAKS-1’ [152], ‘2013-BAKS-2’ [152], or ‘2013-BAKS-3’ [152].

Other empirical parameterizations have considered a generalization of Eq. (62) in the following form:

63 $$\begin{aligned} r_{ch}(A,Z) = r_0 * \left( 1-b\frac{N-Z}{A} +c\frac{1}{A} \right) A^{1/3}\hbox { fm} . \end{aligned}$$

Note that Eq. (62) is a peculiar case of Eq. (63) for which $r_0=1.2$ MeV and $b=c=0$.

The value of the parameters $r_0$, *b*, and *c* for the different empirical adjustments are given in Table 5.

### Neutron-skin thickness

For the neutron-skin thickness, the nuda toolkit provides a set of experimental measurements and theoretical predictions. The complete nuclei list in the nuda toolkit is given with the following instructions: 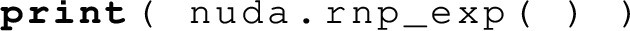


The available experimental/analysis data for the neutron-skin thickness ($R_\textrm{np}=R_n-R_p$) closely follow the organization given in Ref. [153], in which the authors summarize a comprehensive set of results in Tables III and IV of the Appendix.

**Fig. 43:**
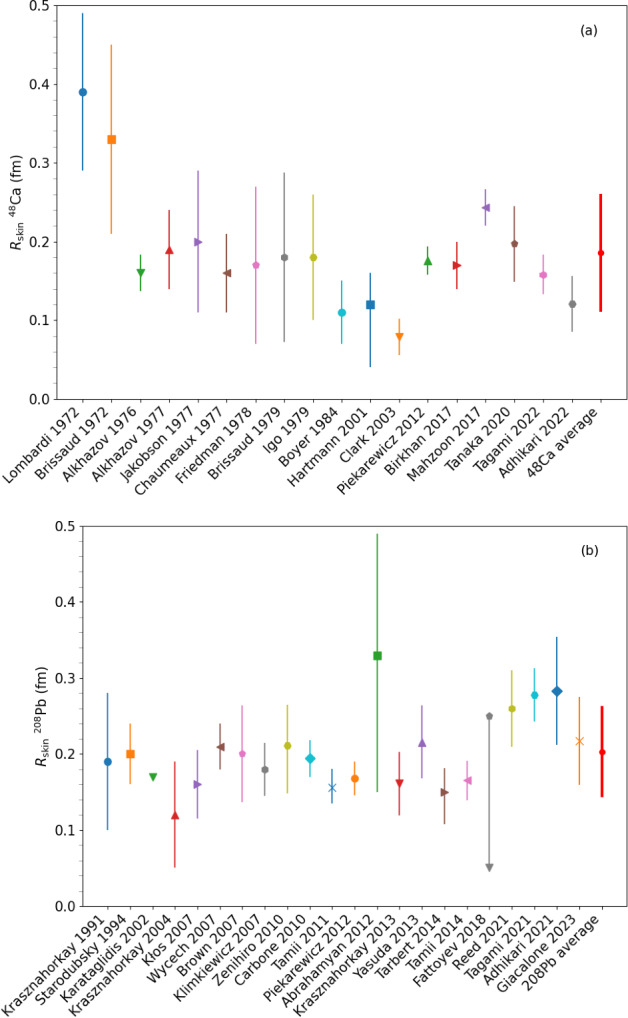
Data deduced by different experiments and analyses for neutron skin thickness of (a) $^{48}\textrm{Ca}$ and (b) $^{208}\textrm{Pb}$, based on the available datasets in the nuda toolkit. Figures generated with nuc_setupRnpExp_plot.py

We currently have two data sources available in the toolkit, namely, ‘48Ca’ and ‘208Pb’, corresponding to the respective nuclei. Once the variable source is chosen, the information regarding the experimental/analysis is performed in the following way: 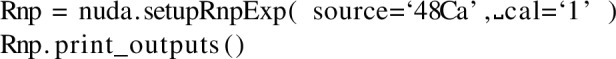


In the source ‘48Ca’, we have a total of 18 data points available extracted from a variety of experimental and analysis methods. Each of the 18 data points is assigned to the variable cal = 1, ..., 18. The references corresponding to these points are [129, 154–170], and data are shown in Fig. 43(a). For the source ‘208Pb’, we have a total of 22 data points available with similar structure (cal = 1, ..., 22). The respective references are [125, 128, 166, 171–189], and in Fig. 43(b) we display the data.

The theoretical predictions for $R_\textrm{np}$ are calculated by using some parametrizations implemented in the nuda toolkit ($R_p$ and $R_n$ are obtained from the method used in Ref. [132]). The complete list is accessed from the command: 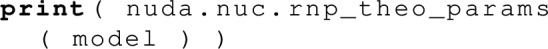 We have three models in this calculation: ‘Skyrme’, ‘NLRH’, and ‘DDRH’. Once the model is chosen, $R_\textrm{np}$ for $^{48}$Ca and $^{208}$Pb are listed in the nuda toolkit from the command: 
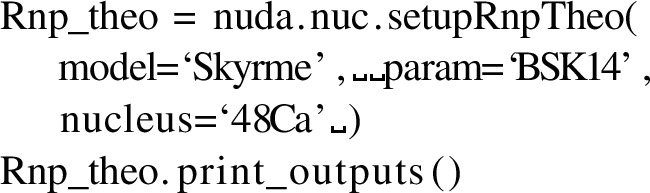

**Fig. 44:**
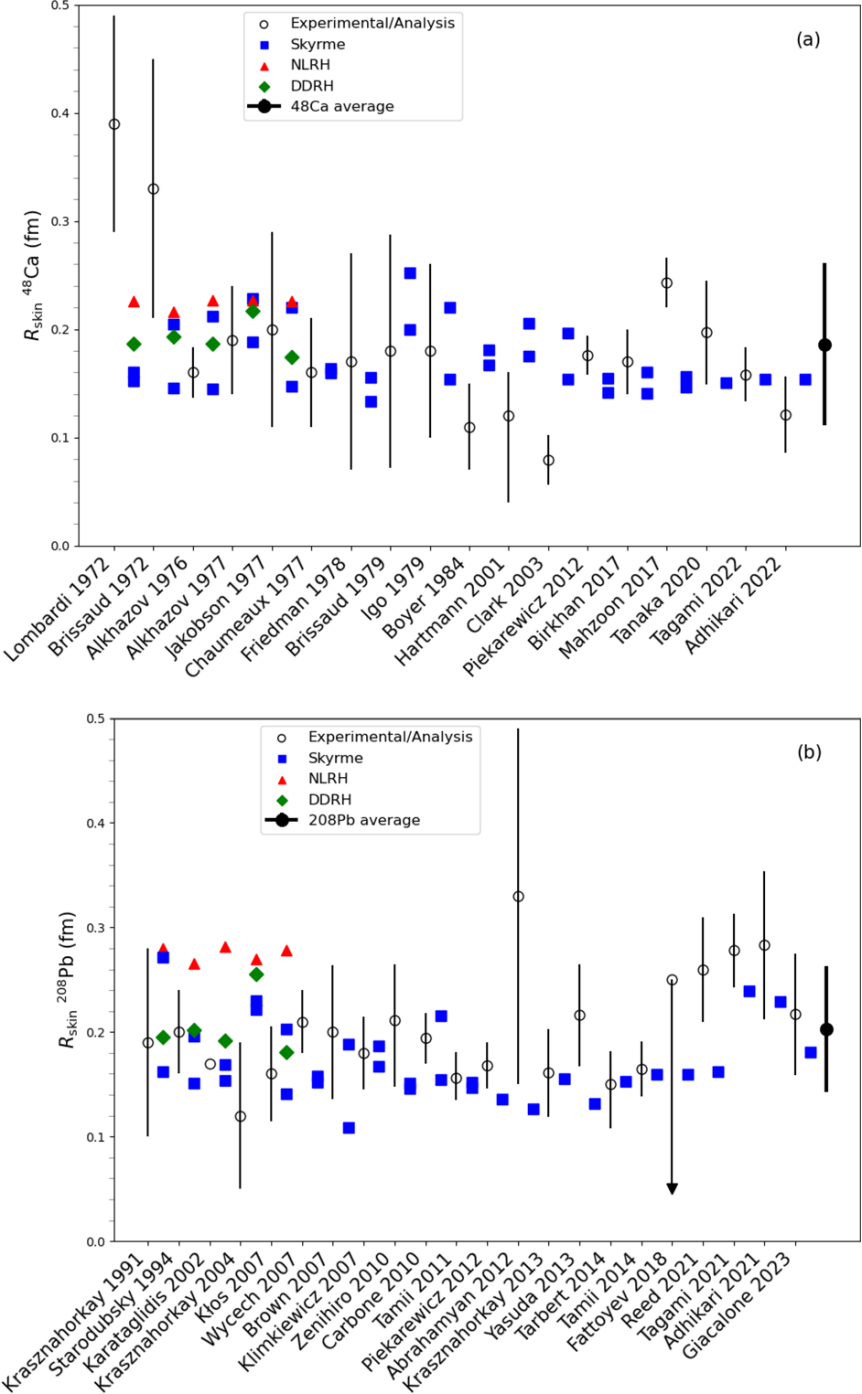
Theoretical neutron skin thickness for (a) $^{48}\textrm{Ca}$ and (b) $^{208}\textrm{Pb}$, as predicted by models implemented in the nuda toolkit. The experimental/analysis data from Fig. 43 are also shown. Figures generated with nuc_setupRnpTheo_plot.py

The theoretical predictions of $R_\textrm{np}$ for $^{48}\textrm{Ca}$ and $^{208}\textrm{Pb}$ are shown in Fig. 44. They can be directly compared to the experimental ones shown in Fig. 43.

### Experimental centroid energy for the ISGMR

The ISGMR, or breathing mode, is often employed to evaluate the goodness of the nuclear modeling. The complete list of available experimental tables is given with the following instructions: 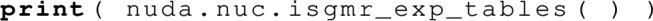 We have two tables available for the moment: ‘2018-ISGMR-LI’ [190] and ‘2018-ISGMR-GARG’ [113]. Once the variable table is chosen, the call for the experimental ISGMR can be performed in the following way: 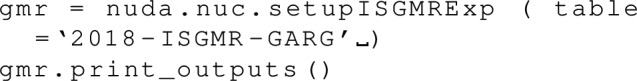


We now provide more details about the experimental ISGMR tables available in the nuda toolkit.

table= ‘2018-ISGMR-LI’.

Table from Ref. [190].

table= ‘2018-ISGMR-GARG’.

Table from Ref. [113].

table= ‘2018-ISGMR-GARG-LATEX’.

Original table from Ref. [113].

table= ‘2022-average’.

Average table employed in Ref. [191].

Note that the nuda toolkit provides only experimental data for the ISGMR energy. To select some isotopes from the table, there is a select() function that can be called in the following way: 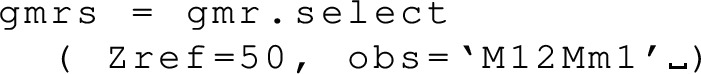 where Zref selects the isotope charge and obs the observable, that can be taken from the following options: ‘M12M0’ ($m_1/m_0$), ‘M12Mm1’ ($\sqrt{m_1/m_{-1}}$) or ‘M32M1’ ($\sqrt{m_3/m_1}$). The output arrays are: gmrs.nucA for the mass *A* of the isotopes, gmrs.cent for the centroid of the GMR energy (as defined from obs), gmrs.errp, gmrs.errm, gmrs.erra for the positive, negative, and average uncertainties.

**Fig. 45:**
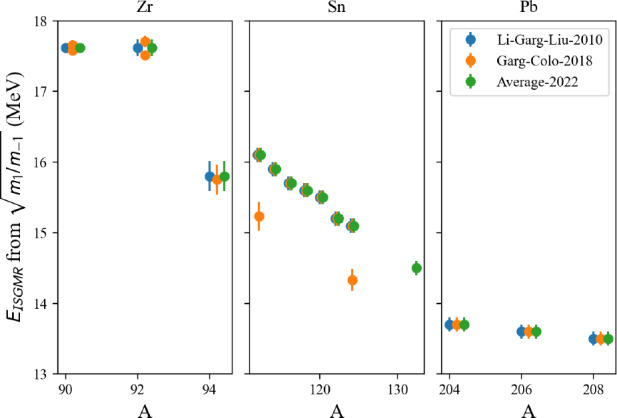
ISGMR energies available in the nuda toolkit extracted from the sum rule $\sqrt{m_1/m_{-1}}$. Figure generated with nuc_setupISGMRExp_plot.py

We show in Fig. 45 a comparison between the three tables available in the nuda toolkit. Except for the nuclei in the Sn isotopic chain, the two tables provide comparable results for the isotopes shown in Fig. 45.

## Data for hypernuclei: the hnuc module

Hypernuclei are bound systems composed of nucleons and one or more hyperons (baryons with strangeness content) such as $\varLambda$, $\varSigma$, $\varXi$, or $\varOmega$. Since their discovery in 1951, nowadays more than 40 single-$\varLambda$ hypernuclei, and few double-$\varLambda$ and single-$\varXi ^-$ ones have been identified. In contrast, it has not been possible to prove without any ambiguity the existence of $\varSigma$-hypernuclei.

**Fig. 46:**
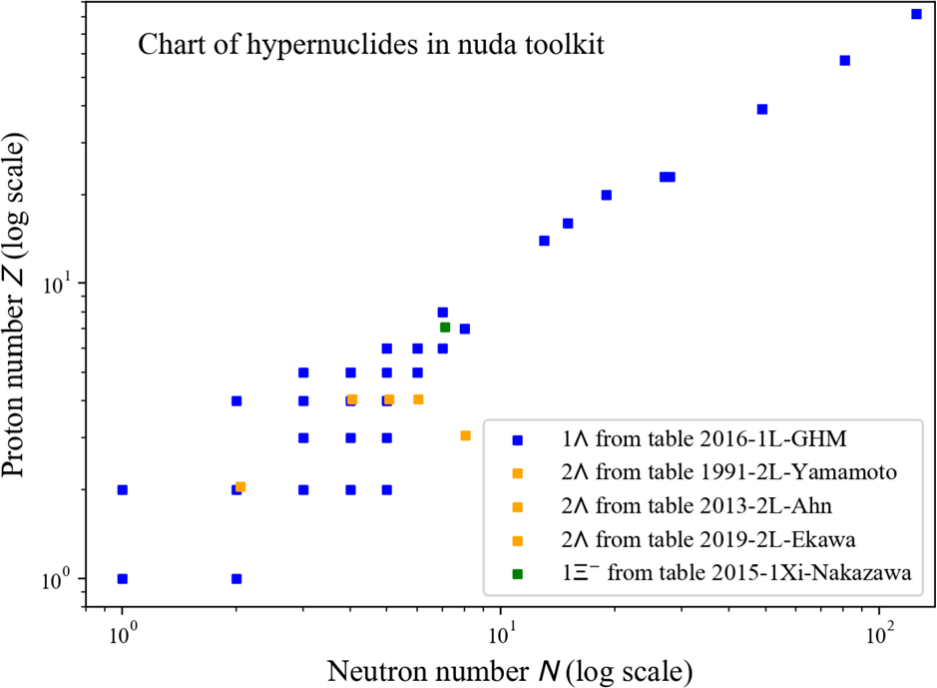
Hypernuclear chart representing the hypernuclides available in the nuda toolkit as a function of *N* and *Z* (log scale). The hypernuclei from tables ’1991-2 L-Yamamoto’ and ’2019-2 L-Ekawa’ are ambigous and should be undertaken with due caution (see text for more details). Figure generated with hnuc_setupChart_plot.py

We adopt the usual nomenclature for single $\varLambda$, $\varSigma$ or $\varXi$ hypernuclei, $^{A}_Y Z$, with $A=N+Z+1$, $Y=\varLambda$, $\varSigma$ or $\varXi$, and *Z* the atomic number of the pure nuclear core that forms the hypernuclei. For double $\varLambda$ hypernuclei, the nomenclature is $^{A}_{YY} Z$ with $A=N+Z+2$. The total energy for the hypernucleus $^{A}_{N_Y} Z$ is defined as:

64 $$\begin{aligned} E_\textrm{tot}(Z,N,N_Y) = Z m_p + N m_n + N_Y m_Y + BE(^{A}_{N_Y}Z) , \end{aligned}$$

where $BE(^{A}_{N_Y}Z)$ is the binding energy and is defined as a negative number for bound nuclei.

The hypernuclear chart, see Fig. 46, shows, as a function of *N* and *Z*, the hypernuclides available in the nuda toolkit tables that we describe in the next subsections. These nuclei have a single $\varLambda$, a double $\varLambda$, or a single $\varXi ^{-}$; see legend for more details. Over the past six decades, only a small number of double-$\varLambda$ hypernuclei have been experimentally observed, mainly through emulsion and hybrid-emulsion techniques at CERN, KEK, and J-PARC. The most unambiguous and universally accepted identification is the NAGARA event [192], corresponding to $^6_{\varLambda \varLambda }$He, which provided a precise $\varLambda \varLambda$ binding energy of $\varDelta B_{\varLambda \varLambda }= 0.67 \pm 0.17$ MeV [193]. Several additional candidate events, such as $^{10}_{\varLambda \varLambda }$Be and $^{13}_{\varLambda \varLambda }$B reported in Ref. [194], and the more recent MINO event from the J-PARC E07 experiment [195], have been interpreted as other possible double-$\varLambda$ systems. However, in these cases, the parent hypernucleus assignments remain ambiguous, owing to incomplete decay-chain reconstruction, uncertainties in the kinematics of captured $\varXi ^-$ hyperons, or the possible involvement of excited nuclear states. As a consequence, although several $\varLambda \varLambda$-hypernuclear candidate events have now been only the NAGARA event can be regarded as uniquely identified. Therefore, any quantitative or phenomenological use of the data provided in the toolkit for the additional and ambiguously identified double-$\varLambda$ hypernuclei should be undertaken with due caution.

### Single-$\varLambda$ hypernuclei

Single-$\varLambda$ hypernuclei can be produced by several reaction mechanisms such as $K^-+^AZ\rightarrow ^A_\varLambda Z + \pi ^-$ strangeness exchange reactions, where a neutron hit by a $K^-$ is changed into a $\varLambda$ emitting a $\pi ^-$; $\pi ^+ + ^AZ \rightarrow ^A_\varLambda Z + K^+$ associated production reactions, where an $s\bar{s}$ pair is created from the vacuum and $K^+$ and a $\varLambda$ are produced in the final state; electro-production $e^-+^AZ\rightarrow e^-+K^+ + ^A_\varLambda (Z-1)$; or by using stable and unstable heavy ion beams. The binding energies of the produced single-$\varLambda$ hypernuclei can be accurately determined by measuring the momenta of the incoming and outgoing kaons and pions with the help of magnetic spectrometers. Here we provide an analysis published in Ref. [196] considering the results of several probes, see the list hereafter.

**Fig. 47:**
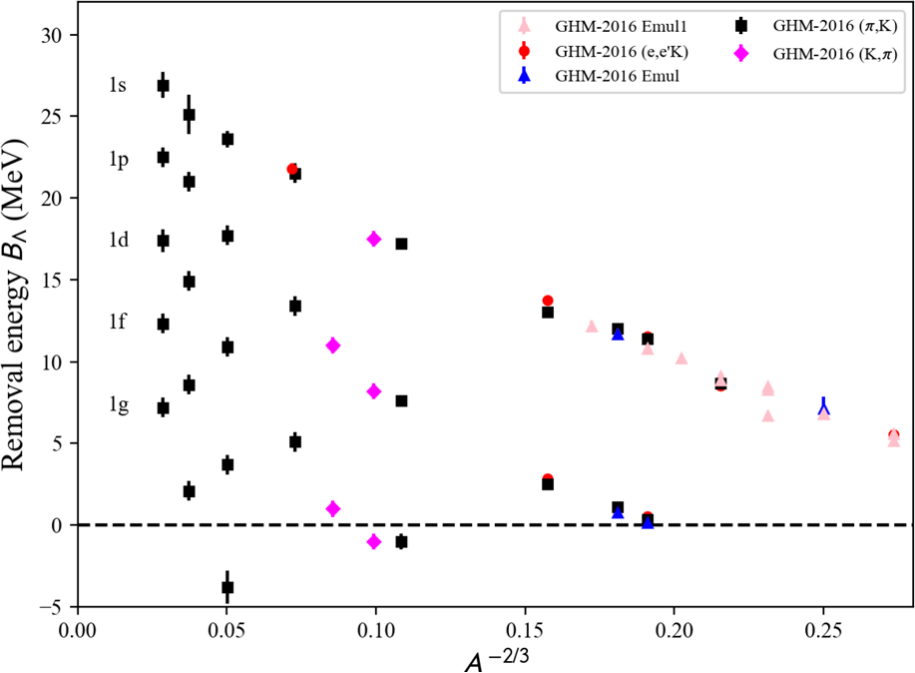
Experimental removal energies for single-$\varLambda$ hypernuclei available in the nucleardatapy toolkit. Figure generated with hnuc_setupRE1LExp_plot.py

The complete list of available tables is given with the following instructions: 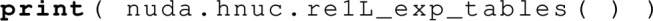 In the present release of nuda toolkit, there is only one table which has been implemented: table=‘2016-1 L-GHM’. The data are obtained from tables I and IV of Ref. [196]. The experimental table is loaded in the following way: 
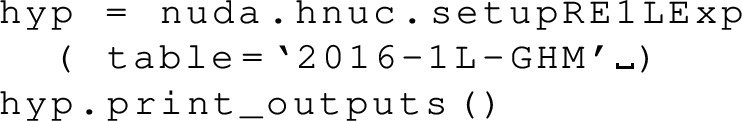
 This table provides the $\varLambda$ removal energy $B_\varLambda$, defined as

65 $$\begin{aligned} B_{\varLambda }= &   E_\textrm{tot}(Z,N-1) + m_\varLambda - E_\textrm{tot}(Z,N-1,N_{\varLambda }=1) , \nonumber \\= &   BE(^{A-1} Z)- BE(^{A}_\varLambda Z ) , \end{aligned}$$

for the hypernucleus $^{A}_\varLambda Z$. This energy is associated with a single-particle state and measure its energy (with opposite sign), see Fig. 47. The other properties of this single particle state, e.g., its angular momentum, are obtained by the experimental analysis.

The attributes are the following Numpy arrays: hyp.A the baryon number ($A=N+Z-S$), hyp.N the neutron number *N*, hyp.Z the proton number *Z*, hyp.Q the nuclear charge ($Q=Z-q_SS$), where $q_S$ is the charge of the hyperon particule ($q_\varLambda =0$ for instance), hyp.S the strangeness number ($S=-1$ for 1 $\varLambda$ for instance), and hyp.symb the symbol representing the hypernucleus.

Since measurements have been performed with various excitation energies, states with different angular momenta have been identified, corresponding to different single-particle energies in the ground state of single-$\varLambda$ hypernuclei. These states are shown in Fig. 47 and are provided as attributes to the object hyp in the following way: hyp.sps the single-particle state, hyp.ell its associated angular momentum, hyp.lre and hyp.lre_err the removal energy and its experimental uncertainty, and finally hyp.probe the probe employed to extract the experimental information: ‘piK’ for $(\pi ^+,K^+)$, ‘eeK’ for $(e,e^\prime K^+)$, ‘emul1’ for emulsion obtained from table I, ‘emul’ for emulsion obtained from table IV, and ‘Kpi’ for $(K^-,\pi ^-)$.

### Double-$\varLambda$ hypernucleus

Double-$\varLambda$ hypernuclei are nowadays the best systems to investigate the properties of the strangeness S=-2 baryon-baryon interaction. Contrary to single-$\varLambda$ hypernuclei, double-$\varLambda$ hypernuclei cannot be produced in a single reaction. To produce them, first a $\varXi ^-$ has to the created, through reactions like $K^-+p\rightarrow \varXi ^-+K^+$ or $p+\bar{p}\rightarrow \varXi ^+ + \bar{\varXi }^+$. Then, provided the $\varXi ^-$ is captured in an atomic orbit, it can interact in a second step with the nuclear core, producing two $\varLambda$ hyperons via processes such as, e.g., $\varXi ^-+p\rightarrow \varLambda + \varLambda + 28$ MeV, where the 28 MeV energy is equally shared by the two $\varLambda$’s. Earlier emulsion experiments reported the formation of a few double-$\varLambda$ hypernuclei: $^{\,\,\,6}_{\varLambda \varLambda }$He, $^{\,\,10}_{\varLambda \varLambda }$Be and $^{\,\,13}_{\varLambda \varLambda }$B. However, the identification of some of these double-$\varLambda$ hypernuclei was ambiguous. In 2001, a new $^{\,\,\,6}_{\varLambda \varLambda }$He candidate was unambiguously observed at KEK in Japan. Further experiments are planned in the future at BNL, KEK, and J-PARC with $K^-$ beams, and at FAIR/GSI with proton and antiproton beams.

The list of available tables is given with the following instructions: 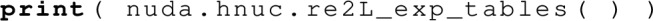 There are several tables for double-$\varLambda$ hypernuclei in the toolkit: table = ‘1991-2 L-Yamamoto’ [194], table = ‘2013-2 L-Ahn’ [193], and table = ‘2019-2 L-Ekawa’ [195]. These tables contain the binding energy $B_{\varLambda \varLambda }$ and the bond energy $\varDelta B_{\varLambda \varLambda }$. The $\varLambda \varLambda$ excess binding energy, also called the bond energy, $\varDelta B_{\varLambda \varLambda }$ for the nucleus $(Z,N,2\varLambda )$ is defined as [196]:

66 $$\begin{aligned} \varDelta B_{\varLambda \varLambda }= &   B_{\varLambda \varLambda }(^A_{\varLambda \varLambda }Z) - 2B_{\varLambda }(^{A-1}_{\varLambda }Z) , \end{aligned}$$

67 $$\begin{aligned}= &   BE(^{A}_{\varLambda \varLambda }Z)+BE(^{A-2}Z) -2 BE(^{A-1}_{\varLambda }Z) , \end{aligned}$$

where the $\varLambda \varLambda$ binding energy is defined as,

68 $$\begin{aligned} B_{\varLambda \varLambda }(^A_{\varLambda \varLambda }Z)=BE(^{A-2}Z)- BE(^A_{\varLambda \varLambda }Z) . \end{aligned}$$

Note that the bond energy can be defined equivalently as [197, 198]:

69 $$\begin{aligned} \varDelta B_{\varLambda \varLambda }= &   E_\textrm{tot}(^{A}_{\varLambda \varLambda }Z)+E_\textrm{tot}(^{A-2}Z) -2 E_\textrm{tot}(^{A-1}_{\varLambda }Z) . \end{aligned}$$

The instantiation of the object hyp with the mass table is performed in the following way: 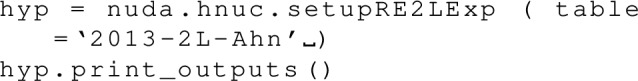

**Table 6:** Data from tables available in the nuda toolkit, see Refs. [193–196, 199]: $B_{Y}$ and $B_{YY}$ are the removal energies, and $\varDelta B_{YY}$ is the bond energy, see text for more details. As discussed in the text, we remind that among the double-$\varLambda$ hypernuclei, only the NAGARA event is unambiguous, the identication of the others remain still uncertain. The use of these data should therefore be taken with care. This table has been generated with hnuc_setupRE1LExp_script.py, hnuc_setupRE2L Exp_script.py and hnuc_setupRE1XiExp_script.py

Z	N	S	Q	Name	$B_Y$ (MeV)		Ref.
						$$\varDelta B_{YY}$$ (MeV)	
single-$\varLambda$ hypernucleus							
2	2	-1	2	He	$3.12\pm 0.020$		[196]
single-$\varXi ^{-}$ hypernucleus							
7	7	-2	6	N	$4.378\pm 0.250$		[199]
					$$B_{YY}$$ (MeV)		
double-$\varLambda$ hypernucleus							
4	4	-2	4	Be	$8.50\pm 0.70$	$-4.90\pm 0.70$	[194]
3	8	-2	3	B	$27.60\pm 0.70$	$4.80\pm 0.70$	[194]
2	2	-2	2	He	$6.910\pm 0.160$	$0.670\pm 0.170$	NAGARA [193]
4	4	-2	4	Be	$15.05\pm 0.11$	$1.63\pm 0.11$	MINO [195]
4	5	-2	4	Be	$19.07\pm 0.11$	$1.87\pm 0.37$	MINO [195]
4	6	-2	4	Be	$13.68\pm 0.11$	$-2.70\pm 1.00$	MINO [195]

The content of nuda toolkit for double-$\varLambda$ is shown in Table 6, together with the $B_Y$ for 1-$\varLambda$ and 1-$\varXi$ hypernuclei. We remind that the strangeness number of $\varLambda$ and $\varXi$ are $S_\varLambda =-1$ and $S_\varXi =-2$.

The attributes of the object hyp are: hyp.A the baryon number ($A=N+Z-S$), hyp.N the neutron number *N*, hyp.Z the proton number *Z*, hyp.Q the nuclear charge *Q*, hyp.S the strangeness number *S*, and hyp.symb the symbol representing the hypernucleus. Are also provided the $\varLambda \varLambda$ removal energy $B_{\varLambda \varLambda }$ as hyp.llre and its experimental error hyp.llre_err (in MeV), and the bond energy $\varDelta B_{\varLambda \varLambda }$ as hyp.lldre, with experimental uncertainty hyp.lldre_err (in MeV). Finally, the attribute hyp.probe contains the probe employed to extract the experimental information.

### Single-$\varXi ^{-}$ hypernucleus

Single-$\varXi ^{-}$ hypernuclei can be produced via the reactions $K^-+p\rightarrow \varXi ^-+K^+$ or $p+\bar{p}\rightarrow \varXi ^+ + \bar{\varXi }^+$. Nowadays, very few single-$\varXi$ hypernuclei have been identified, although future production experiments of $\varXi ^-$ hypernuclei are being planned at the J-PARC facility in Japan.

The list of available tables is given with the following instructions: 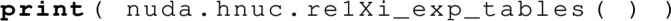 The toolkit provides a single table: table = ‘2015-1Xi-Nakazawa’. This table is constructed from Ref. [199] where the so-called ‘Kiso’ event related to the creation of $^{15}_{\varXi ^-}N$ is reported. The instantiation of the object hyp with the table is performed in the following way: 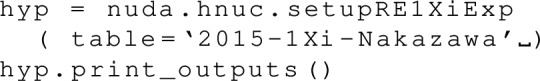


The $\varXi ^{-}$ removal energy is defined as

70 $$\begin{aligned} B_{\varXi ^{-}}=BE(^{A-1} Z)-BE(^A_{\varXi ^{-}} Z) , \end{aligned}$$

and the content of nuda toolkit for the single-$\varXi ^{-}$ event is shown in Table 6.

The attributes of the object hyp are: hyp.A the baryon number ($A=N+Z-S$), hyp.N the neutron number *N*, hyp.Z the proton number *Z*, hyp.Q the nuclear charge *Q*, hyp.S the strangeness number *S*, and hyp.symb the symbol representing the hypernucleus. The $\varXi$ removal energy $B_{\varXi }$ and its experimental error are also provided in hyp.xire and hyp.xire_err (in MeV). Finally, the attribute hyp.probe contains the probe employed to extract the experimental information.

## Neutron star crust: the crust module

The crust of NSs is a non-uniform system composed of nuclear clusters embedded in an electron gas (outer crust) and with additional contributions from a neutron fluid (inner crust). There are several calculations for the crust of NSs, and some of them are provided by the nuda toolkit.

The complete list of available crust model predictions is given with the following instructions: 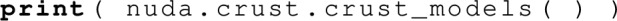 The object crust is instantiated in the following way: 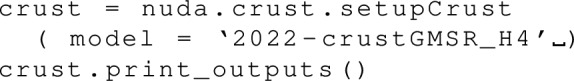 where the variable model is fixed to one of the following crust models.

model=‘1973-Negele-Vautherin’.

This provides results of the Hartree–Fock calculation for the inner crust of NS from Ref. [200].

model=‘2018-PCPFDDG-BSK22’, ‘2018-PCPFDDG-BSK24’, ‘2018-PCPFDDG-BSK25’, ‘2018-PCPFDDG-BSK26’.

The crust is obtained from BSK22, BSK24, BSK25, and BSK26 extended Skyrme EDF and based on the 4th-order Extended Thomas-Fermi (ETF) method with proton shell correction via the Strutinsky integral (SI) [201]. The nucleon distributions are parametrized using damped Fermi profiles.

model=‘2020-MVCD-D1S’, ‘2020-MVCD-D1M’, ‘2020-MVCD-D1M$^*$’.

The crust is obtained from D1S, D1M, and D1M$^*$ Gogny interactions using a semiclassical variational Wigner-Kirkwood method along with shell and pairing corrections calculated with the Strutinsky integral method and the BCS approximation [202].

model=‘2022-crustGMSR_BSK14’, ‘2022-crustGMSR_BSK16’, ‘2022-crustGMSR_DHSL59’, ’2022-crustGMSR_DHSL69’, ‘2022-crustGMSR_F0’, ’2022-crustGMSR_H1’, ‘2022-crustGMSR_H2’, ’2022-crustGMSR_H3’, ‘2022-crustGMSR_H4’, ’2022-crustGMSR_H5’, ‘2022-crustGMSR_H7’, ’2022-crustGMSR_LNS5’, ‘2022-crustGMSR_RATP’, ’2022-crustGMSR_SGII’, ‘2022-crustGMSR_SLY5’.

The GMSR crust [203] is computed with the meta-model [204, 205] calibrated to different parametrizations of the Skyme force (BSK14 [54], BSK16 [55], F0 [60], LNS5 [61], RATP [63], SGII [65], SLy5 [75]) and to Chiral EFT Hamiltonians(H1-H7 [38] (except H6), and DHS$_{L59}$-DHS$_{L69}$ [24])) using a compressible liquid drop model (CLDM) approach as detailed in Ref. [206].

**Fig. 48:**
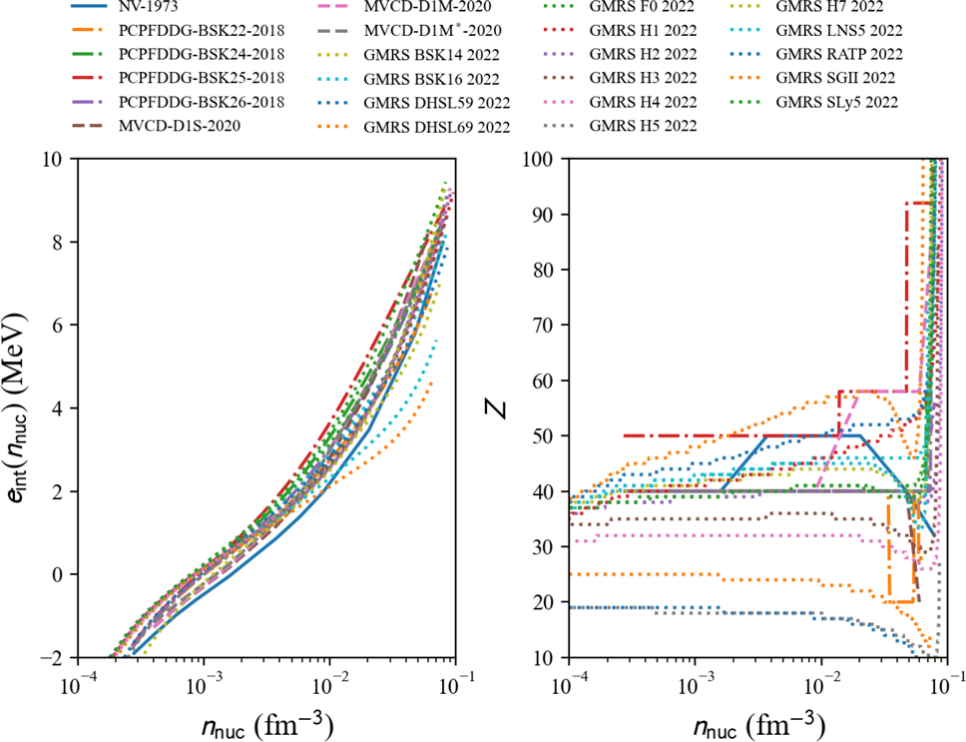
Properties of the crust, internal energy per nucleon $e_\textrm{int}$ and proton number *Z* as a function of the nucleon density $n_\textrm{nuc}$ provided by the models available in the nuda toolkit. Figure generated with crust_setupCrust_plot.py

The attributes of the object crust are Numpy arrays: crust.den the nucleon density $n_\text {nuc}$ in fm$^{-3}$ (crust.den _cgs in g cm$^{-3}$), crust.A and crust.A_bound the total number of nucleons *A* in a Wigner-Seitz cell with radius $R_{WS}$ and volume $V_{WS}$ (in crust.RWS and crust.VWS) and the number of bound nucleons $A_\text {bound}$ in a cluster with radius $R_{cl}$ and volume $V_{cl}$ (in crust.Rcl and crust.Vcl), crust.Z and crust.Z_bound the total number of protons *Z* and the number of bound protons $Z_\text {bound}$, crust.N and crust.N_bound the total number of neutrons *N* and the number of bound neutrons $N_\text {bound}$, crust.N_f the number of free neutrons ($N_f=N-N_\text {bound}$). Note that crust.N_bound and crust.N_f are not provided for the crust models with prefix ‘2020-MVCD’. crust.den_f is the free neutron density $n_f$, crust.u is the volume fraction $u=V_{cl}/V_{WS}$, and finally crust.e2a_tot, crust.eps_tot and crust.e2a _int are the total energy per nucleon, the total energy density and the internal energy per nucleon. More attributes can be obtained in the following way: 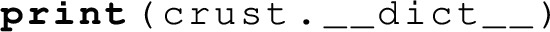


A few properties in the NS crust, internal energy per nucleon and proton number, are represented in Fig. 48 for the models available in the nuda toolkit. The densities are selected to represent the properties of the inner crust. There is a large dispersion in the internal energies and in the proton number, which is related to the fact that most of the EDF do not reproduce neutron fluid properties accurately. This point is well discussed in Refs. [203, 206].

## Equation of state: the eos module

We now describe the eos module, where nucleon and lepton contributions are considered together.

The classes employed in this section are located in the eos module. The new feature in the eos class is that lepton contribution is added to the nucleon thermodynamic quantities, adding two variables: the electron density $n_e$ and the muon density $n_\mu$. At $T=0$, four components contribute to dense matter: *n*, *p*, *e*, and $\mu$. We also consider the electro-neutrality condition: $n_p=n_e+n_\mu$. There are, therefore, only three independent components.

In the following, we consider asymmetric matter (AM), controlled by the nucleon density, the proton, and the muon fractions; the lepton-equilibrated matter (also imposing $\mu _e=\mu _\mu$) controlled by the nucleon density and the proton fraction; and the beta-equilibrated matter (adding the $\beta$-equilibrium condition $\mu _n-\mu _p=\mu _e$) controlled only by the nucleon density.

### Ground state of asymmetric matter

For the models predicting SM and NM, the energy in AM is defined in the following way:

71 $$\begin{aligned} e_\textrm{nuc}(n,\delta ) \approx e_{\textrm{SM}}(n)+e_{\textrm{sym},2}(n) \delta ^2 , \end{aligned}$$

where $e_{\textrm{sym},2}\approx e_{\textrm{sym}}$, defined by Eq. (43).

Similarly, the pressure reads

72 $$\begin{aligned} p_\textrm{nuc}(n,\delta ) \approx p_{\textrm{SM}}(n)+p_{\textrm{sym}}(n) \delta ^2 , \end{aligned}$$

where $$p_{\textrm{SM}}=n^2(\partial e_\textrm{SM}/\partial n)$$ and $$p_{\textrm{sym}}=n^2(\partial e_\textrm{sym}/\partial n)$$.

The ground state of uniform matter at fixed electron and muon fractions is governed by the following equations:

- nucleon number: $$n_\textrm{nuc}=n_n+n_p$$;
- charge neutrality: $n_p=n_e$ if only electrons, while $n_p=n_e+n_\mu$ with electrons and muons;
- no neutrinos: $$\mu _\nu =0$$.

In the following, we investigate only models providing results in SM and NM, since the symmetry energy $e_\textrm{sym}$ requires SM and NM to be defined. We therefore disregard all models providing results in only NM. The list of models suitable for calculating AM can be obtained in the following way: 
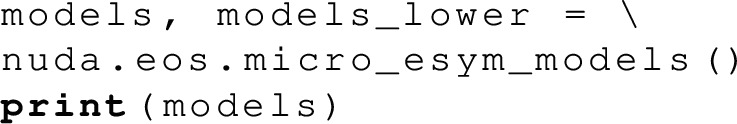


The call of the EoS in AM for a given model, param, and a given kind (which can be either ‘micro’ or ‘pheno’) can be done in the following way: 
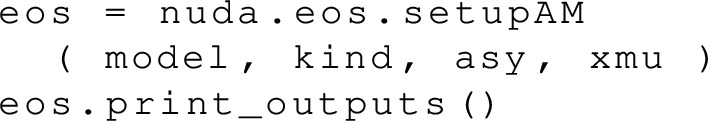
 where the scalar variable asy is the isospin asymmetric parameter (asy=0 by default, for SM), and the scalar variable xmu is the muon fraction (xmu=0 by default, only electrons). Note that the density mesh is the one fixed by the class nuda.matter.setupMicroEsym or nuda.matter.setupPhenoEsym. It corresponds to the densities where the symmetry energy is calculated. If kind=‘micro’, then the variable param is not used and can be set to param=None.

**Fig. 49:**
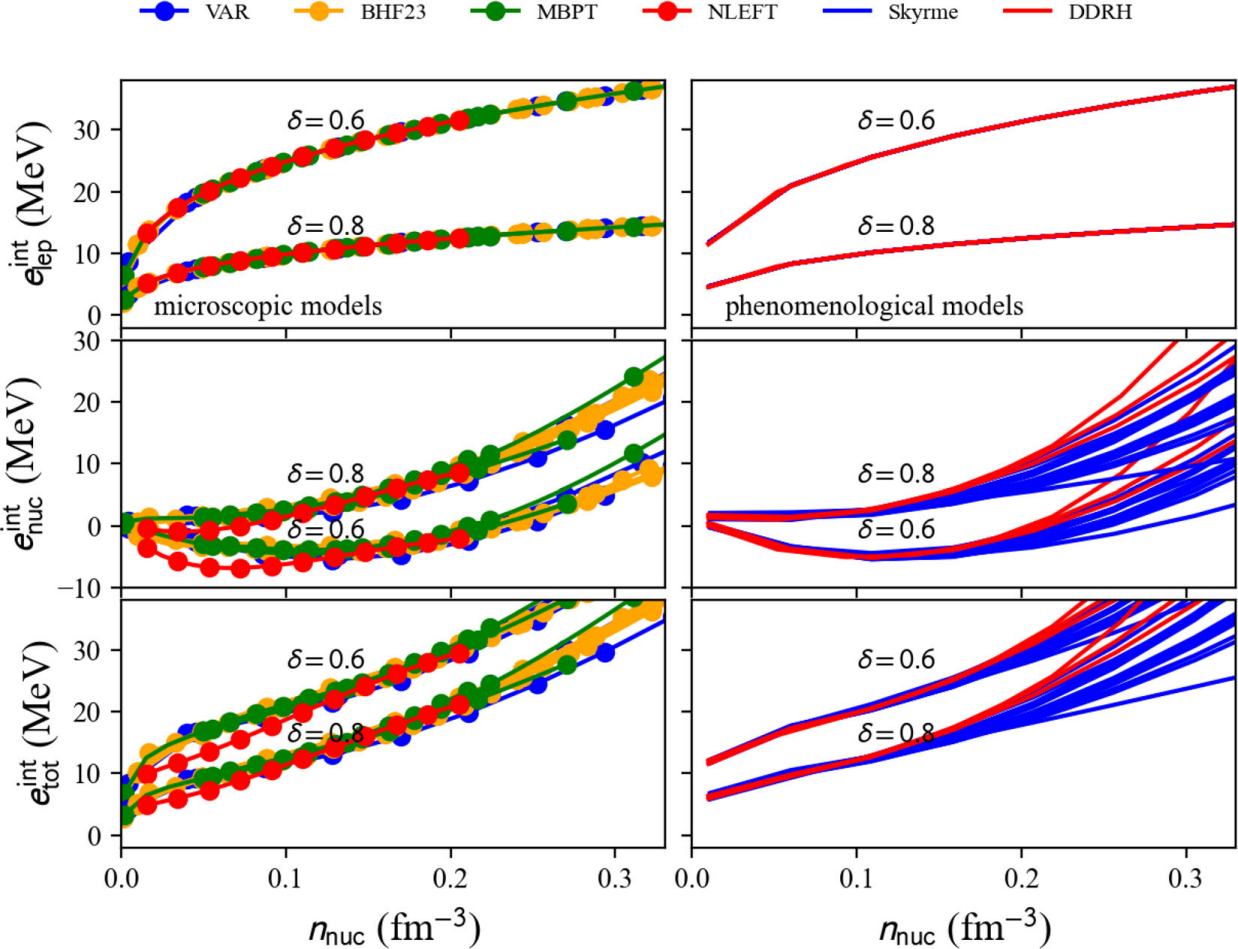
Energy per particle in neutron-rich matter (with $\delta =0.6$ and 0.8) for the list of microscopic (left) and phenomenological (right) models available in the nuda toolkit. Figure generated with eos_setupAM_e2a_plot.py

We show in Fig. 49 the internal energy per nucleon for the lepton contribution $E_\text {lep}^\text {int}/A$, for the nuclear contribution $E_\text {nuc}^\text {int}/A$, and for the total energy per nucleon $E_\text {tot}^\text {int}/A$, running over all microscopic (left) and phenomenological (right) models available in the toolkit. The asymmetry parameter asy is fixed to two values: asy=0.6, 0.8. All models shown are the ones passing inside the reference band in NM, that we previously described in Sect. 3.5. Note the opposite behavior of the energy per nucleon for the lepton contribution and for the nuclear contribution as a function of the asymmetry parameter $\delta$: as $\delta$ increases, the number of leptons decreases and the energy per nucleon decreases as well. Inversely, the nucleon energy per particle increases as a function of $\delta$. Since the reduction of the lepton energy per nucleon is larger than the increase of the nuclear component, the total energy per particle decreases as a function of $\delta$. This phenomenon is observed for microscopic and phenomenological models.

Attributes are the following (Numpy arrays): the nucleon density $n_\textrm{nuc}$ is given by eos.den, the neutron and proton densities $n_n$ and $n_p$ ($n_\textrm{nuc}=n_n+n_p$) are given in eos.n_n and eos.n_p. The electron, muon and lepton densities are in eos.n_el, eos.n_mu, and eos.n_lep. The thermodynamic properties are: the total energy per nucleon eos.e2a_tot, its nucleon contribution eos.e2a_nuc and lepton contribution eos.e2a_ lep, the symmetry energy eos.esym, the total energy density eos.eps_tot, the total pressure eos.pre_tot and the sound speed eos.cs2_tot. More attributes can be obtained in the following way: 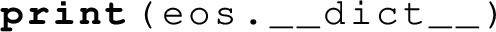


### Ground state of lepton-equilibrated matter

The ground state of lepton-equilibrated matter at fixed proton fraction is governed by the following equations:

- baryon number: $$n_\textrm{nuc}=n_n+n_p$$;
- charge neutrality: $n_p=n_e$ if only electrons, $n_p=n_e+n_\mu$ with electrons and muons;
- chemical potentials: $\mu _\mu =\mu _e$ when muons are present, $\mu _\mu =0$ otherwise.
- no neutrinos: $$\mu _\nu =0$$;

At $T=0$, if $\mu _e \le m_\mu c^2$, only electrons are present, $n_\mu =0$, and $x_p=x_e$, while if $\mu _e>m_\mu c^2$ there are electrons and muons in asymmetric matter, $x_p=x_e+x_\mu$ and $n_l=k_{Fl}^3/(3\pi ^2)$ with $l=e$, $\mu$, and the following relation between lepton Fermi momenta imposes the equilibrium:

73 $$\begin{aligned} \left( m_\mu c^2\right) ^2 + \left( \hbar c k_{F\mu } \right) ^2 = \left( \hbar c k_{Fe} \right) ^2 . \end{aligned}$$

Lepton-equilibrated EoS in AM, for a given nuclear model and kind (which can be either ‘micro’ or ‘pheno’), is contained in the object Leq instantiated in the following way: 
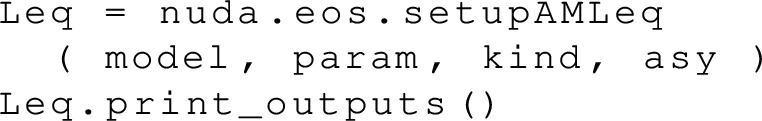
 where the (scalar) variable asy is the isospin asymmetric parameter, with $-1<$asy$<1$.

**Fig. 50:**
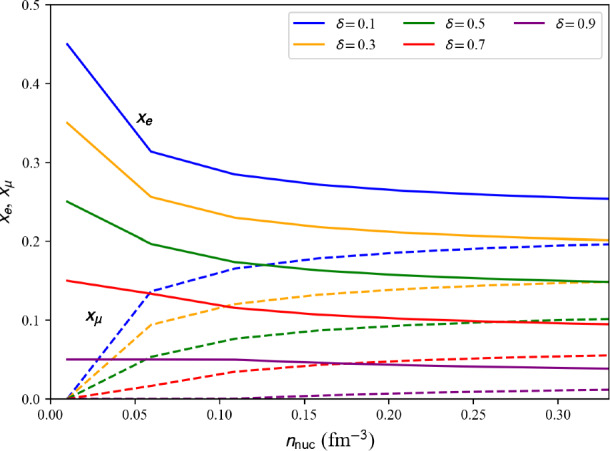
Lepton fractions $x_e$ (solid lines) and $x_\mu$ (dashed lines) for lepton-equilibrated AM with various values for $\delta$ from 0.1 up to 0.9 (see legend). Figure generated with eos_setupAMLeq_plot.py

The lepton fractions $x_e$ and $x_\mu$ for lepton-equilibrated AM as functions of the nucleon density $n_\text {nuc}$ are shown in Fig. 50. The asymmetry parameter $\delta$ is varied from 0.1 up to 0.9, as shown in the legend. Note that these fractions are independent of the nuclear EoS, so the quantities shown in Fig. 50 are identical for all nuclear models.

Attributes are the same as in AM (Numpy arrays): the nucleon density $n_\textrm{nuc}$ is given by Leq.den, the neutron and proton densities $n_n$ and $n_p$ ($n_\textrm{nuc}=n_n+n_p$) are given in Leq.n_n and Leq.n_p. The electron, muon and lepton densities are in Leq.n_el, Leq.n_mu, and Leq.n_lep. The thermodynamic properties are: the total energy per nucleon Leq.e2a_tot, its nucleon contribution Leq.e2a_nuc and lepton contribution Leq.e2a_lep, the symmetry energy Leq.esym, the total energy density Leq.eps_tot, the total pressure Leq.pre_tot and the sound speed Leq.cs2_tot. More attributes can be obtained in the following way: 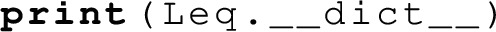


### Ground state of $\beta$-equilibrated matter

The ground state of matter at beta-equilibrium is governed by the following equations:

- baryon number: $$n_\textrm{nuc}=n_n+n_p$$;
- charge neutrality: $n_p=n_e$ if only electrons, $n_p=n_e+n_\mu$ with electrons and muons;
- chemical potentials: $\mu _e+\mu _p=\mu _n$ if only electrons; we have to add the following relation: $\mu _e=\mu _\mu$ when muons are present;
- no neutrinos: $$\mu _\nu =0$$.

There is only one additional equation in $\beta$-equilibrated matter compared to the lepton-equilibrated case, $\mu _e+\mu _p=\mu _n$, fixing the asymmetry parameter $\delta$. As a consequence, there is only one independent variable that we choose to be the nucleon density $n_\textrm{nuc}$.

Considering the quadratic approximation (71), we have $\mu _n-\mu _p\approx 4\, \delta \, e_{\textrm{sym},2}(n_\textrm{nuc})$ and since $\mu _e=\hbar c k_{Fe}=\hbar c( 3\pi ^2 n_e)^{1/3}$ for ultra-relativistic electrons, we obtain the following equation to be solved at $\beta$-equilibrium:

74 $$\begin{aligned} 4\, e_{\textrm{sym},2}(n_\textrm{nuc}) \left( 1-2x_p \right) \approx \hbar c (3\pi ^2 x_e n_\textrm{nuc})^{1/3} \end{aligned}$$

where $x_p=n_p/n_\textrm{nuc}$, $x_e=n_e/n_\textrm{nuc}$, and $n_\textrm{nuc}$ is fixed. Equation (74) can be solved with the initial solution:

75 $$\begin{aligned} x_e\approx (4e_{\textrm{sym},2}/\hbar c)^3/[3\pi ^2 n_\textrm{nuc}+6(4e_{\textrm{sym},2}/\hbar c)^3] , \end{aligned}$$

assuming $x_e\ll 1$ and $x_p=x_e$, for low density matter.

The EoS at $\beta$-equilibrium for a given model, param, and kind (which can be either ‘micro’ or ‘pheno’) is contained in the object beta that is instantiated in the following way: 
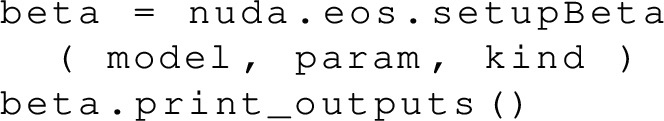

**Fig. 51:**
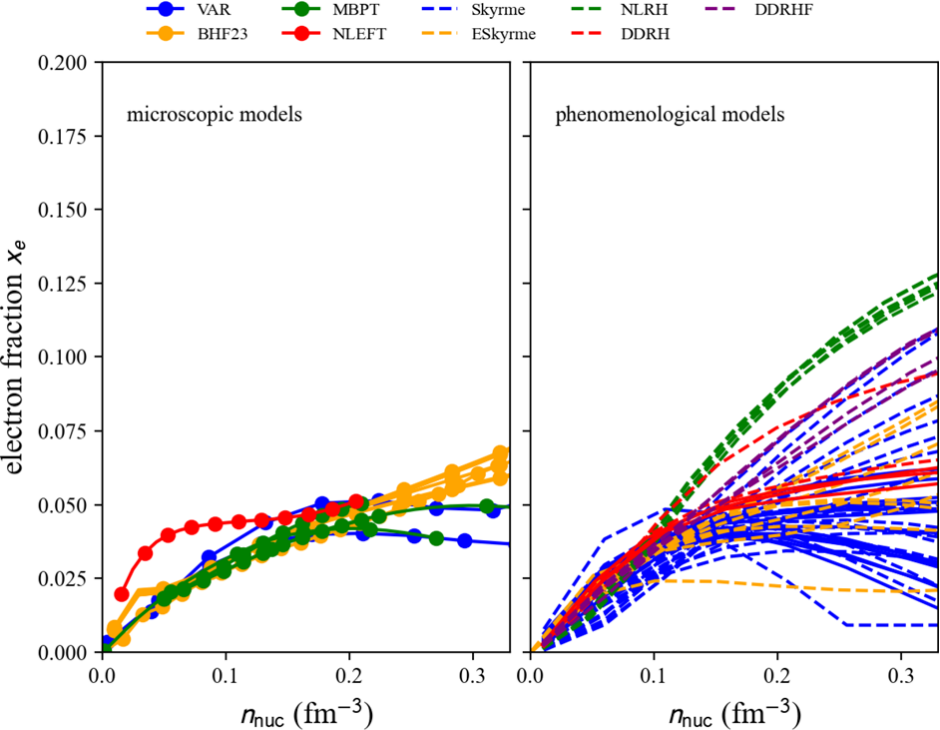
Electron fraction for microscopic models (left) and phenomenological models (right) in $\beta$-equilibrium matter. Figure generated with eos_setupAMBeq_plot.py

**Fig. 52:**
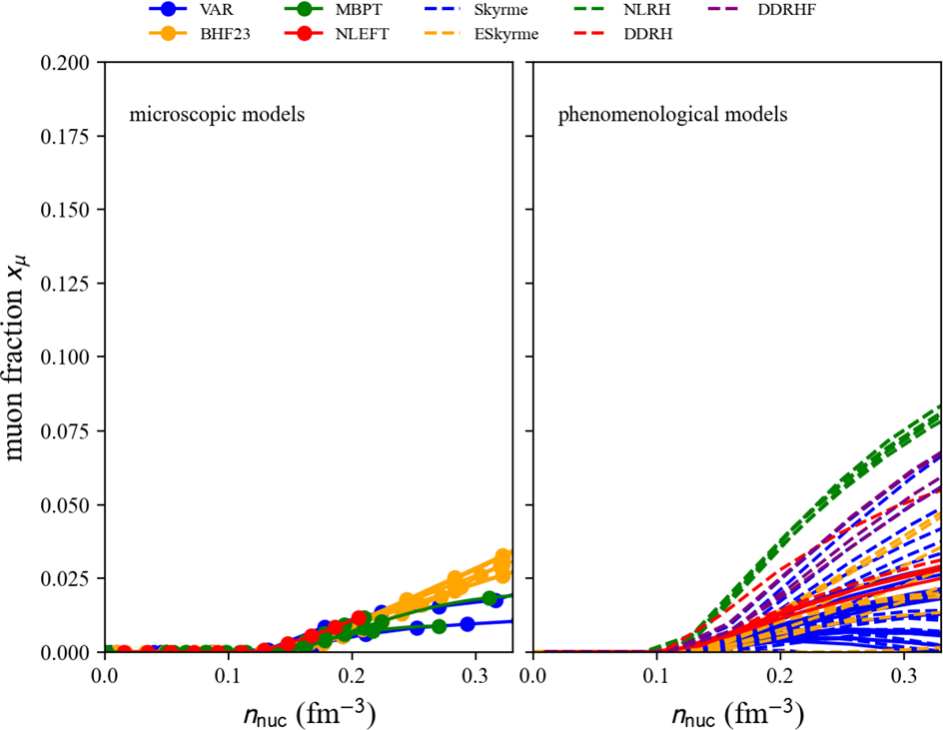
Same as Fig. 51 for the muon fraction

**Fig. 53:**
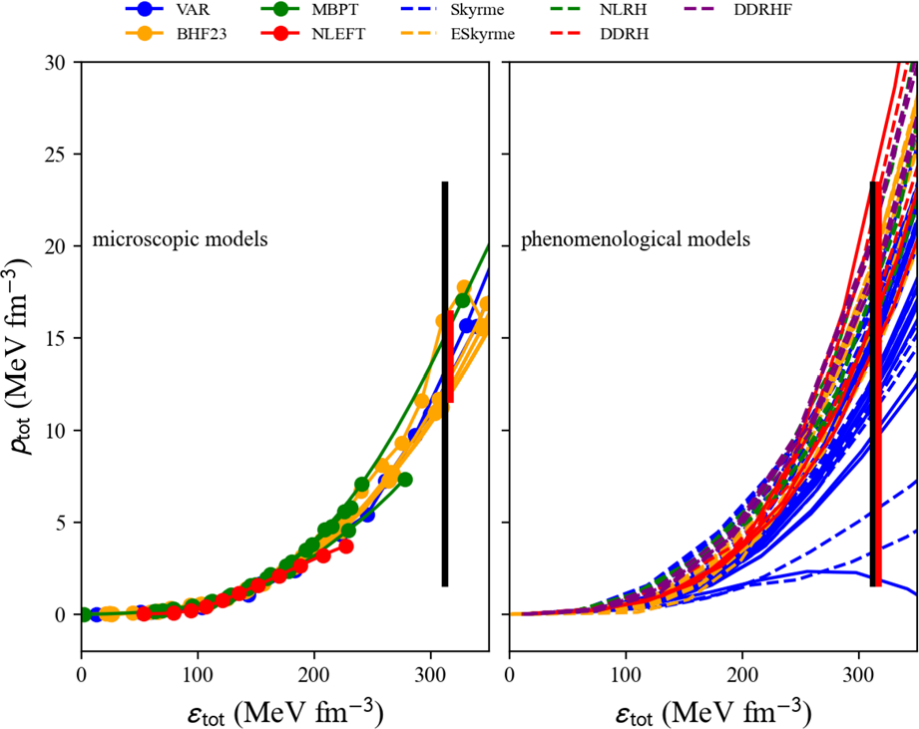
Pressure in matter at beta-equilibrium for the list of microscopic (left) and phenomenological (right) models available in the nuda toolkit. Figure generated with eos_setupAMBeq_plot.py

Running over all microscopic and phenomenological models available in the toolkit, we show the electron fraction in Fig. 51, and the muon fraction in Fig. 52. For all the microscopic and phenomenological models considered in our meta-analysis, muons appear for densities above 0.1 fm$^{-3}$ (for phenomenological models) and 0.15 fm$^{-3}$ (for microscopic models).

The pressure in nuclear matter at beta-equilibrium as a function of the energy density $\epsilon _\text {tot}$ is shown in Fig. 53. The vertical lines represent the dispersion in the microscopic models, phenomenological models, and total at twice the saturation energy density. The dispersion for the phenomenological models is large and is the widest one. The uncertainties are estimated for the models that match the reference band in NM, and they are shown in solid lines. It can be remarked that the models that are not consistent with the reference band (in dashed lines) would not contribute to increasing the uncertainties for the total pressure at twice the saturation energy density. The large dispersion in the prediction observed for the phenomenological models cannot be reduced by imposing the models to be consistent with the reference band. In particular, the very soft phenomenological model largely contributes to the uncertainty in the pressure. This model predicts the collapse of nuclear matter, but it could not be disregarded if there is a low-density phase transition (just above the saturation density) bringing the necessary repulsion to avoid the collapse.

Attributes are the same as in AM (Numpy arrays): the nucleon density $n_\textrm{nuc}$ is given by beta.den, the neutron and proton densities $n_n$ and $n_p$ ($n_\textrm{nuc}=n_n+n_p$) are given in beta.n_n and beta.n_p. The electron, muon, and lepton densities are in beta.n_el, beta.n_mu, and beta.n_lep. The thermodynamic properties are: the total energy per nucleon beta.e2a_tot, its nucleon contribution beta.e2a_nuc and lepton contribution beta.e2a _lep, the symmetry energy beta.esym, the total energy density beta.eps_tot, the total pressure beta.pre_tot and the sound speed beta.cs2_tot. More attributes can be obtained in the following way: 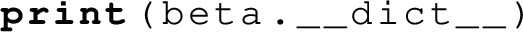

**Table 7:** Pressure and uncertainties in MeV fm$^{-3}$ for NM, SM, and for matter at beta-equilibrium and at $2\epsilon _{\textrm{sat}}$ (with $\epsilon _\textrm{sat}=$156 MeV fm$^{-3}$ and $\rho _{\textrm{sat}}=2.8\times 10^{14}$ g cm$^{-3}$). These quantities are also represented in Figs. 24, 25 and 53. They are compared to the inference from gravitational wave analysis presented in Ref. [207], see text for more discussion

Micro	Pheno	Total
In NM:		
[8.5 : 25.5]	[8.5 : 37.5]	[8.5 : 37.5]
In SM:		
[4.5 : 17.5]	[11.0 : 33.0]	[4.5 : 33.0]
In matter at beta-equilibrium:		
[11.5 : 16.5]	[1.5 : 23.5]	[1.5 : 23.5]
From gravitational wave detection (90% C.I.): [207]		
		$$21.9^{+16.9}_{-10.6}$$

Finally, in Table 7 are given the uncertainties for the pressure in NM, SM, and for AM at $\beta$-equilibrium shown in Figs. 24, 25 and 53. The dispersion in the pressure is obtained from meta-analyses running over all microscopic models (micro), phenomenological models (pheno), or assembling all predictions together (total). We remind that the dispersion is estimated only for the models in agreement with the reference band in NM. It is a pure nuclear prediction since results for these nuclear models for neutron stars are not considered here. These nuclear predictions are also compared with the one inferred from the gravitational-wave detection GW170817 [207]. There is a pretty good overlap between the nuclear-physics prediction and the one inferred from gravitational-wave measurements. It can, however, be noted that the predictions from nuclear physics favor the lower values of pressure as predicted by gravitational-wave analyses. In conclusion of the present meta-analysis, there is a good agreement between nuclear physics and gravitational-wave inferences for the pressure at twice the saturation energy density, with a preference, for nuclear models, for the half lower uncertainty of the gravitational-wave estimation of the pressure.

Improvements from gravitational-wave analyses require new observations of an event similar to GW170817, while the improvement from nuclear physics requires tighter constraints, such as, for instance, those associated with heavy-ion collisions or microscopic models.

### Connecting crust and core EoS

Since the nuda toolkit contains a set of crust and core equations of state at $\beta$-equilibrium, it is possible to connect the crust and the core EoS and provide a complete EoS describing neutron stars in their ground state.

The construction of an EoS for NSs requires: (i) a crust EoS, (ii) a core EoS at $\beta$-equilibrium, and (iii) a method to connect the crust and the core. This is usually the case since a given equation of state does not work for all densities. The following class provides such a construction: 
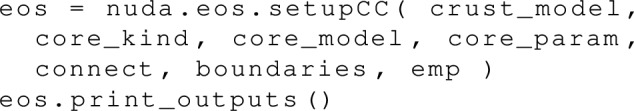
 where the variable crust_model fixes the crust EoS by choosing among the models described in Sect. 7, for memory, the list of crust models is available as 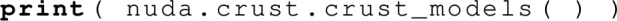


The variable core_kind fixes the kind of model for the core: ‘micro’ or ‘pheno’. The variable core_model fixes the model for the core and the variable core_param fixes the parameter set for the core in case core_kind=‘pheno’. If core_kind=‘micro’, the user can fix core_param =None. The list of microscopic models for the core model can be accessed by 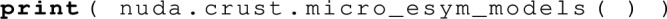 For phenomenological models, the list of core models is available as 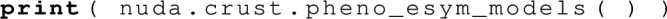 For a chosen model, the list of parameter sets can be printed as 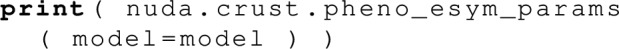


To connect the core and the crust equations of state, we use the variable connect. One can choose to connect them in nucleonic density (connect=‘density’), in energy-density (connect=‘epsilon’) or in pressure (connect=‘pressure’). In all cases, the upper boundary of the crust has to be fixed, which also coincides with the lower limit for the connection (den_lo, rho_lo, pre_lo), as well as the lower boundary of the core, coinciding with the upper limit of the connection (den_up, rho_up, pre_up). The variable boundaries contains the doublet value (den_lo, den_up) if connect= ‘density’. If connect=‘epsilon’, then boundaries=(eps_lo, eps_up) and if connect= ‘pressure’, then boundaries=(pre_lo, pre_up).

The new EoS connecting the crust and the core is discretized on log-log scale, and a linear interpolation is considered between the connection boundaries.

The attributes of the object eos are: eos.den, eos.pre, eos.eps and eos.cs2 for the particle density, the pressure, the energy density, and the square of the sound speed.

The transition between the core and the crust can be fixed by the user as explained previously, by fixing the lower and upper limits of the variable boundary. It can also be fixed by using empirical relations that are encoded in the nuda toolkit in the following way: 
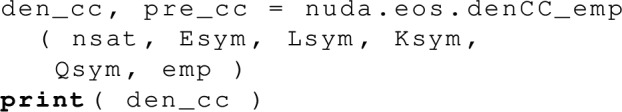
 providing den_cc in fm$^{-3}$, and for the input variable emp which can be set to:

emp=‘Simple’

It simply assumes the crust-core transition density to be half that of the saturation density:

76 $$\begin{aligned} n_{cc} = \frac{n_\textrm{sat}}{2} \, \end{aligned}$$

or

emp=‘Ducoin’

**Fig. 54:**
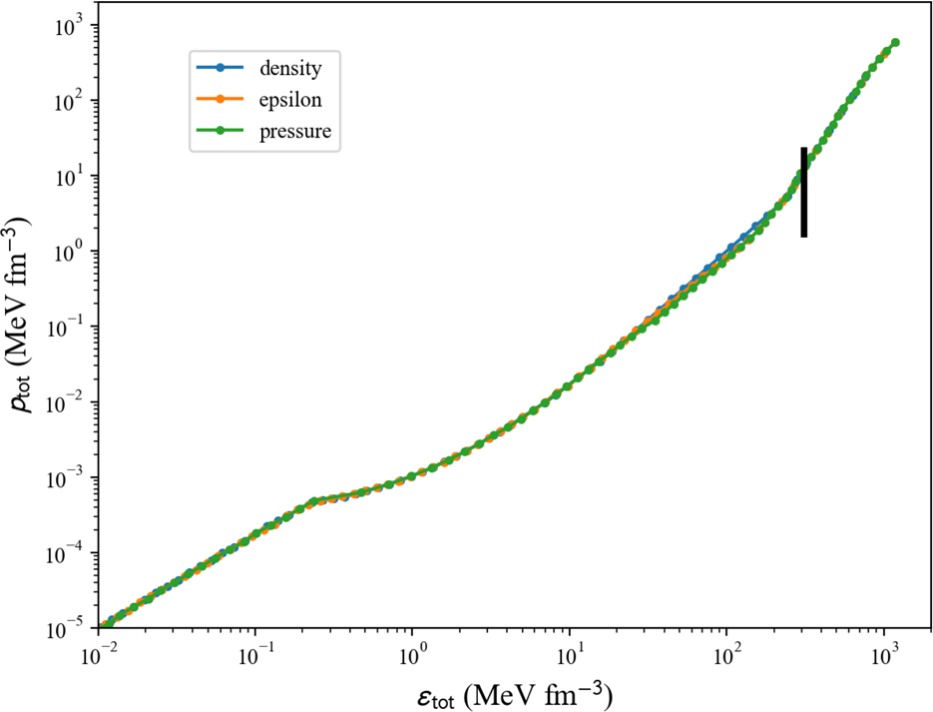
Crust+Core EoS: pressure *p* as a function of the energy density $\epsilon$ for three connections: ‘density’, ‘epsilon’ and ‘pressure’, see text. For the crust, we took ‘2022-GMRS-H4’ and for the core ‘1998-VAR-AM-APR’. The vertical bar represents the pressure uncertainty at $2\epsilon _\textrm{sat}$ from nuclear physics, see table 7. This figure is generated with eos_setupCC_eos_plot.py

This empirical relation is suggested in Ref. [208]:

77 $$\begin{aligned} n_{cc}= &   0.0802 + 3.23\times 10^{-4}\big ( L_{\textrm{sym},0.1} \nonumber \\  &   +\, 0.426 K_{\textrm{sym},0.1}\big ) ,\end{aligned}$$

78 $$\begin{aligned}\approx &   0.0802 + 3.23\times 10^{-4}\big ( L_\textrm{sym}+ (x_{0.1}+0.426) K_\textrm{sym}\nonumber \\  &   + \,0.426 Q_\textrm{sym}x_{0.1}\big ) \, \end{aligned}$$

79 $$\begin{aligned} p_{cc}= &   -\,0.328 + 9.59\times 10^{-3} \big ( L_{\textrm{sym},0.1}, \nonumber \\  &   -\, 0.343 K_{\textrm{sym},0.1} \big ) , \end{aligned}$$

80 $$\begin{aligned}\approx &   -\,0.328 + 9.59\times 10^{-3} \big ( L_\textrm{sym}+(x_{0.1}-0.343) K_\textrm{sym}\nonumber \\  &   -\, 0.343 Q_\textrm{sym}x_{0.1} \big ) , \end{aligned}$$

where $$x_{0.1}=x(n_\textrm{nuc}=0.1$$ fm$$^{-3})$$ and $$x=(n_\textrm{nuc}-n_\textrm{sat})/(3n_\textrm{sat})$$. The NEP $$L_{\textrm{sym},0.1}$$ and $$K_{\textrm{sym},0.1}$$ are empirical parameters defined at 0.1 fm$$^{-3}$$ reference density [208].

emp=‘Newton’

This empirical relation is suggested in Ref. [209]:

81 $$\begin{aligned} n_{cc}= &   E_{\textrm{sym},30}\big ( 0.135 - 0.098L_{\textrm{sym},70} \nonumber \\  &   + 0.026L_{\textrm{sym},70}^2 \big ) , \end{aligned}$$

82 $$\begin{aligned} p_{cc}= &   -0.724 + 0.0157 \left( L_{\textrm{sym},0.1} - 0.343 K_{\textrm{sym},0.1}^2 \right) , \end{aligned}$$

83 $$\begin{aligned}\approx &   -0.724 + 0.0157 \big ( L_\textrm{sym}- 0.343 K_\textrm{sym}^2 \nonumber \\  &   +(1-2\times 0.343 Q_\textrm{sym})x_{0.1} K_\textrm{sym}\big ) , \end{aligned}$$

where $E_{\textrm{sym},30}=E_\textrm{sym}/30$ and $L_{\textrm{sym},70}=L_\textrm{sym}/70$, $E_\textrm{sym}$ and $L_\textrm{sym}$ being the NEP (in MeV) of the crust EDF.

emp=‘Steiner’

This empirical relation has been employed in Refs. [210, 211]:

84 $$\begin{aligned} n_{cc} = E_{\textrm{sym},30}\left( 0.1327 - 0.0898L_{\textrm{sym},70} + 0.0228L_{\textrm{sym},70}^2 \right) . \end{aligned}$$

To take the values given in the input array boundaries, instead of an empirical relation, one should fix emp=None. Otherwise, the choice for the variable emp is considered above all. We also mention that in the nuda toolkit, boundaries are still considered if an empirical relation is chosen. In the ‘density’ connection, the lower boundary is taken as $0.8 n_{cc}$ and the upper boundary is $1.2 n_{cc}$.

We show a result of the connection between an EoS for the crust and another for the core in Fig. 54. Three cases are investigated: a connection in ‘density’ between 0.016 and 0.16 fm$^{-3}$, in energy density ‘epsilon’ between 15 and 150 MeV fm$^{-3}$, and finally a connection in ‘pressure’ between 0.1 and 1 MeV fm$^{-3}$ (note that the boundaries are well separated from each other). A linear interpolation on a log-log scale is considered in the gap region where the two EoS are connected. The connection method has a weak influence on the EoS, but it would still be interesting to study more systematically the impact of varying the crust and the core EoS. It would also be interesting to analyse the impact of the EoS and of the connection on the NS, as suggested in Ref. [212]. This requires solving the general relativistic hydrostatic equations (TOV), which is currently beyond the scope of the nuda toolkit.

## Astrophysical observations: the astro module

Astrophysical observations have become more and more accurate over the last decades. They can now be employed to select among various EoS. The nuda toolkit, therefore, provides a set of astrophysical observations that are interesting to evaluate models. We first present individual measurements for the masses of neutron stars (NSs) from radio-astronomy and then the total mass of binary NSs (BNSs) from gravitational-wave (GW) observations. We then use these observations to construct the probability profile for the maximum mass of isolated, non-rotating, and non-magnetic NS (M$_\textrm{TOV}$).

### Masses of neutron stars

The observation of massive neutron stars from radio astronomy provides a challenge for many EoS models. We provide a list of several observations of massive neutron stars in table 8 together with several measurements that are provided in the variable obs.

**Table 8:** Observed masses ($M_{\textrm{obs},i}$) and associated uncertainties ($\sigma _{M,i}$) at 68% CL for five of the most massive pulsars. For two of these objects, there are several measurements of the masses, which do not often coincide

source	obs	$M_{\textrm{obs}}$	$\sigma _{M}^{+}$	$\sigma _{M}^{-}$	References
		[$M_{\odot }$]	[$M_{\odot }$]	[$M_{\odot }$]	
PSR J1614–2230	1	1.970	0.040	0.040	[213]
	2	1.928	0.017	0.017	[214]
	3	1.908	0.016	0.016	[215]
	4	1.922	0.015	0.015	[216]
	5	1.937	0.014	0.014	[217]
	av	1.933	0.031	0.031	
PSR J0348+0432	1	2.01	0.04	0.04	[218]
PSR J2215+5135	1	2.27	0.15	0.15	[219]
PSR J1600+3053	1	2.5	0.9	0.7	[215]
	av	2.579	0.792	0.792	
MSP J0740+6620	1	2.14	0.10	0.09	[220]
	2	2.08	0.07	0.07	[221]
	3	1.99	0.07	0.07	[217]
	av	2.071	0.101	0.101	

The complete list of available sources is given with the following instructions: 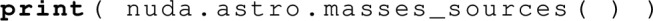


The list of observations for a given source is given with the following instructions: 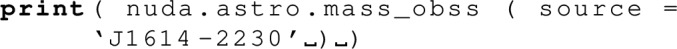 This list of observations is further detailed in Table 8.

Once the variables source and obs are chosen in the previous list, the mass measurement is instantiated in the object m with the following instruction: 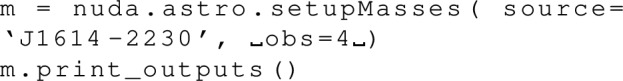


We now provide more details about neutron star masses available in nuda toolkit. There are several sources, some of which have multiple measurements providing different values for neutron star masses.

source=‘J1614–2230’.

obs= 1 [213], 2 [214], 3 [215], 4 [216], 5 [217].

Measured mass for source PSR J1614–2230 obtained from different observations (obs).

source=‘J0348+0432’.

obs= 1 [218]. Measured mass for source PSR J0348+0432 obtained from a single observation (obs).

source=‘J2215+5135’.

obs= 1 [219]. Measured mass for source PSR J2215+5135 obtained from a single observation (obs).

source=‘J1600+3053’.

obs= 1 [215]. Measured mass for source PSR J1600+3053 obtained from a single observation (obs).

source=‘J0740+6620’.

obs= 1 [220], 2 [221], 3 [217].

Measured mass for source MSP J0740+6620 obtained from different observations (obs).

For a given measurement, the attributes are: m.mass is the centroid of the measured mass, m.sig_up and m.sig_lo are the reported upper and lower uncertainty. For a single source, the different measurements can be averaged together in the following way: 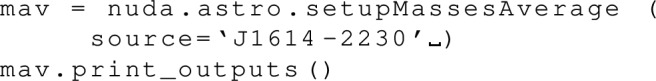 The attributes are the centroid mav.mass_cen and the standard deviation mav.mass_std of the reconstructed measurement (assuming a Gaussian distribution for each measurement).

**Fig. 55:**
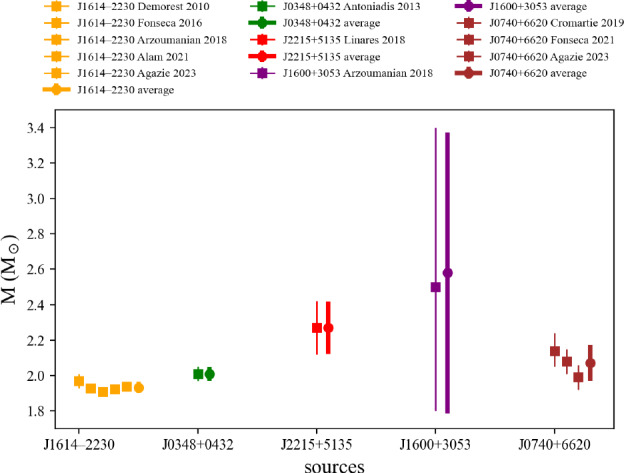
Distribution of measured masses (thin lines) for massive neutron stars and average values (thick lines) defined as the centroid and standard deviation of the cumulative observations per source. Figure generated with astro_setupMasses_plot.py

All the measured masses (thin lines) as well as average values of the measurements per source (thick lines) are shown in Fig. 55. Note that for PSR J1600+3053, the average is not exactly identical to the original measure. It is due to the fact that the original measurement is asymmetric, while the average symmetrizes it. Exact numbers are given in Table 8.

The average quantities provided by the toolkit shall be considered for convenience and are not meant to replace the exact measurements. Note, however, that it may be difficult to choose one measurement instead of another, and the interesting feature of the average masses, illustrated for instance in Fig. 55 is that differences in the measured masses for different sources is larger than the measured uncertainties, except for PSR J1600+3053. The average as we compute it represents well the measurements and suggest an interesting reference quantity.

### Upper masses from gravitational-wave observations

GW emitted from BNSs provides an estimate of the total masses (M$_\textrm{tot}$) of the two merging NSs with a good accuracy. If the final state is a Black Hole (BH), then the total mass can be taken as an estimate of the upper limit of the TOV mass (defined as the maximum mass of non-rotating and non-magnetized NS).

**Table 9:** Masses for GW observation of BNS and for different hypotheses on the spin of the BNSs and the waveform model

Source	Hyp	Hypothesis	*M*	$\sigma _{M}^{+}$	$\sigma _{M}^{-}$	References
			[$M_{\odot }$]	[$M_{\odot }$]	[$M_{\odot }$]	
GW170817	1	low-spin	2.74	0.04	0.01	[222]
		+ TaylorF2				
GW170817	2	high-spin	2.82	0.47	0.09	[222]
		+ TaylorF2				
GW170817	3	low-spin	2.73	0.04	0.01	[223]
		+ PhenomPNRT				
GW170817	4	high-spin	2.77	0.22	0.05	[223]
		+ PhenomPNRT				
GW170817	av		2.825	0.189	0.189	
GW190814	1		2.59	0.08	0.09	[224]
GW190814	av		2.585	0.085	0.085	

The complete list of available GW sources is given with the following instructions: 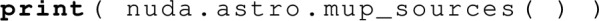


The list of hypotheses for a given source is given with the following instructions: 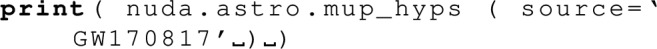 These hypotheses are given in Table 9, where the list of masses for GW observations are reported. For the so-called golden event, GW170817 reported in the toolkit, the total mass $M_\textrm{tot}$ is given based on different hypotheses, e.g., low-spin versus high-spin or gravitational waveforms employed. For the other event GW190814, only one analysis has been carried out.

The object mup contains a measured mass for a given choice for the variables source and hyp. It is instantiated in the following way: 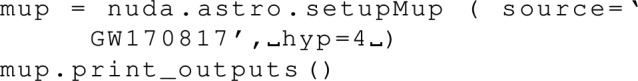 There are several sources, and for some sources, there are several hypotheses in the analysis providing different values for the measured total masses.

source=‘GW170817’.

hyp= 1 [222], 2 [222], 3 [223], 4 [223].

Observed mass for source GW170817 obtained from different hypotheses (hyp), see Table 9 for more details.

source=‘GW190814’.

hyp= 1 [224].

Observed mass for source GW190814 obtained from different hypotheses (hyp), see Table 9 for more details.

**Fig. 56:**
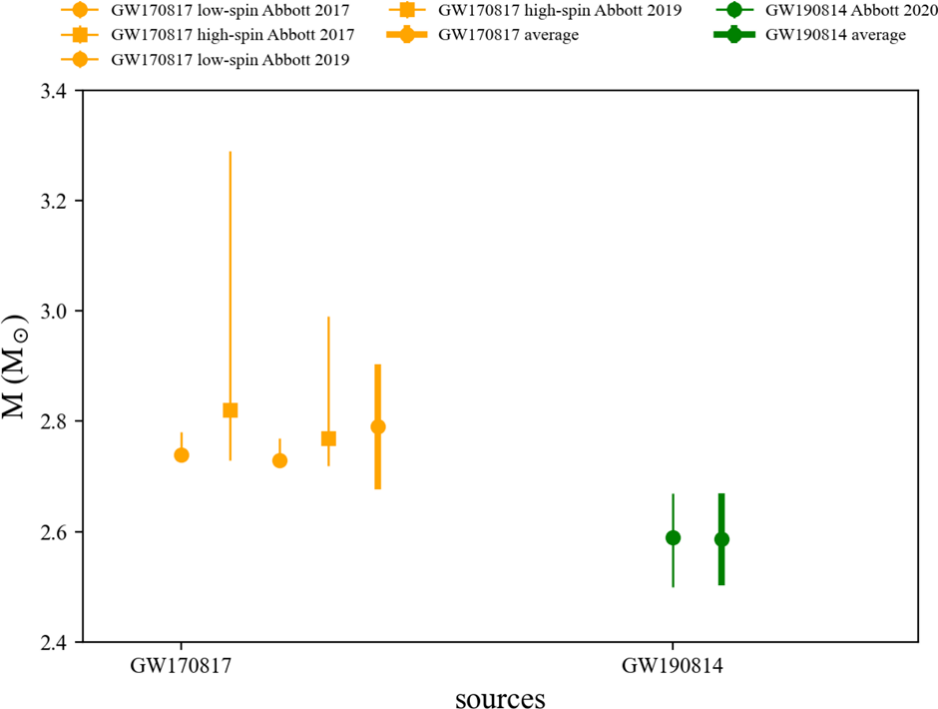
Distribution of upper masses from different GW sources: GW170817 and GW190814. The average for GW170817 is obtained considering only the two most recent analyses, fixing hyps = [3, 4] in the instantiation, as in the example provided in the text. Figure generated with astro_setupMup_plot.py

For a given analysis, the attributes are: mup.mup is the centroid of the measured mass, mup.sig_up and mup.sig_lo are the reported upper and lower uncertainty respectively. For a given source, the different measurements can be averaged together in the following way: 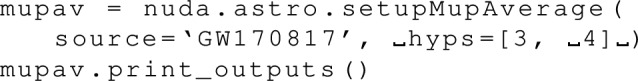 The attributes are the centroid mupav.mass_cen and the standard deviation mupav.mass_std of the reconstructed measurement (assuming a Gaussian distribution for each measurement).

All the observed total masses are shown in Fig. 56, as well as average values of the observations, defined as the centroid and standard deviation of the reconstructed observation (assuming a Gaussian distribution for each hypothesis).

### Probability distribution function for $M_\textrm{TOV}$

The set of observed pulsar masses provides lower limits for $M_\textrm{TOV}$, while the quantity $M_\textrm{tot}$ determined from GW emission from BNS provides an upper limit for $M_\textrm{TOV}$, provided the BNS collapses to a BH after the merger. It is, therefore, possible to estimate the boundaries for $M_\textrm{TOV}$ from astrophysical observations. Moreover, considering the uncertainties in the measurements, it is preferable to determine a distribution of probabilities as a function of $M_\textrm{TOV}$. This is what we are now constructing.

We consider a function of the TOV mass, $z_\textrm{obs}(M_\textrm{TOV})$, defined in the following way:

85 $$\begin{aligned} z_\textrm{obs}(M_\textrm{TOV})= &   \frac{M_\textrm{TOV}-M_{\textrm{obs}}}{\sqrt{2} \, \sigma _{M}^{-}}\varTheta (M_{\textrm{obs}}-M_\textrm{TOV}) \nonumber \\  &   + \frac{M_\textrm{TOV}-M_{\textrm{obs}}}{\sqrt{2} \, \sigma _{M}^{+}}\varTheta (M_\textrm{TOV}-M_{\textrm{obs}}) , \end{aligned}$$

where $\varTheta$ is the Heaviside step function and $M_\textrm{obs}$ and $\sigma _M^{\pm }$ are the observed mass and the associated uncertainties given in Tables 8 (for the lower limit of $M_\textrm{TOV}$) and 9 (for the upper limit).

**Fig. 57:**
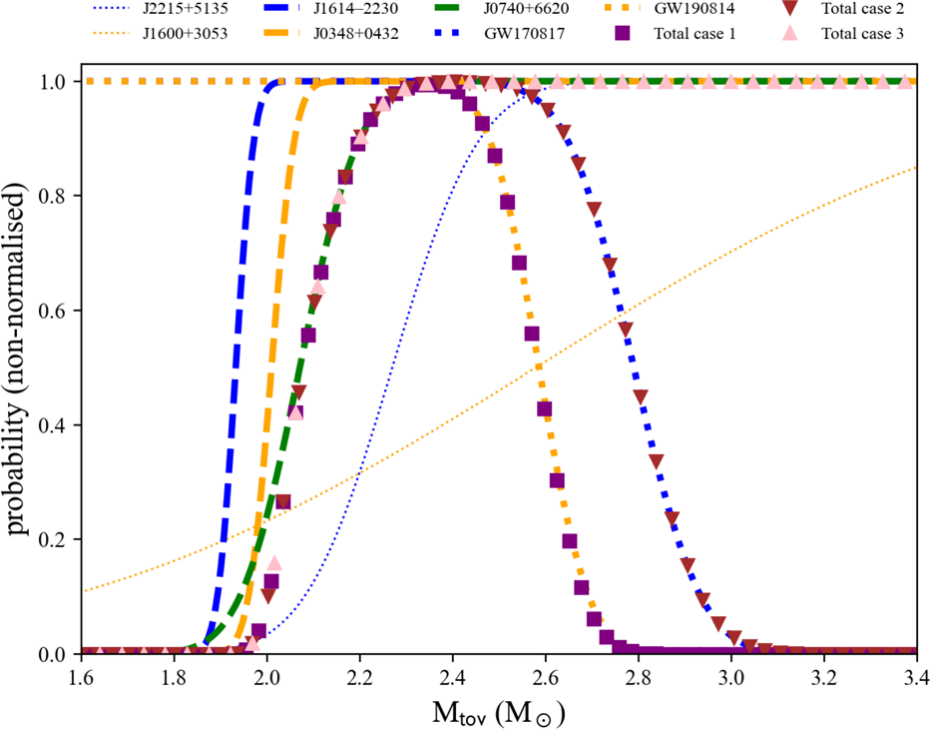
Probability distribution function for the TOV mass (Total, thick solid line or dashed line or dotted line) and the contributions from the different sources (see legend in the figure). Figure generated with astro_setupMtov_plot.py

The probability associated with $M_\textrm{TOV}$ considering a given source is calculated as follows

86 $$\begin{aligned} P_\textrm{obs}(M_\textrm{TOV}) = \frac{\text {erf}[z_\textrm{obs}(M_\textrm{TOV})]+1}{2}, \end{aligned}$$

where $\textrm{erf}(z)$ is the error function defined as $\textrm{erf}(z)=(2/\sqrt{\pi })\int _0^z \exp (-t^2) dt$. The probabilities associated with each observation are shown in Fig. 57, see the five dashed curves for the sources listed in Table 8.

The lower bound probability distribution $P_{\text {lower}}(M_\textrm{TOV})$ is defined as,

87 $$\begin{aligned} P_\textrm{lower}(M_\textrm{TOV}) = \prod _{\textrm{obs}} P_\textrm{obs}(M_\textrm{TOV}) . \end{aligned}$$

$P_\textrm{lower}$ is shown in Fig. 57 as a set of circle symbols. We see that the probability distribution is largely impacted by the highest mass distribution with small uncertainties (here PSR J2215-5135). The role of PSR J1600-3053 is not negligible but remains weak because of the large uncertainties.

The upper limit for non-rotating and non-magnetized neutron stars is more difficult to obtain. GW170817, however, provides an estimation of the upper limit of neutron stars, since it is possible that the two neutron stars have collapsed into a black hole after their merger. This limit depends, however, on the prior for the spin of the neutron stars, see Table 9.

The probability associated with $M_\textrm{TOV}$ considering a given source is calculated as follows,

88 $$\begin{aligned} P_\textrm{Mtot}(M_\textrm{TOV}) = \frac{1-\text {erf}[z_\textrm{obs}(M_\textrm{TOV})]}{2} , \end{aligned}$$

where we consider the average value including the uncertainties in the hypothesis for the spin priors and the waveforms. The upper bound probability distribution $P_{\text {upper}}(M_\textrm{TOV})$ is defined as,

89 $$\begin{aligned} P_\textrm{upper}(M_\textrm{TOV}) = \prod _\textrm{Mtot} P_\textrm{Mtot}(M_\textrm{TOV}) . \end{aligned}$$

The toolkit is ready for several sources, e.g., GW170817 and GW190814, and new sources can be added easily. The dashed line (for GW190814) and the upper probability distribution (square symbols) coincide in Fig. 57. The main contributions come from PSR J2215+5135 and J1600+3053 for the lower boundary and GW190814 for the upper boundary.

Finally, the total probability distribution for the TOV mass is defined as the product of the distribution for the lower masses and the upper masses, as

90 $$\begin{aligned} P_{\text {mass}}(M_\textrm{max}) = P_\textrm{lower}(M_\textrm{max}) \times P_\textrm{upper}(M_\textrm{max}) , \end{aligned}$$

and is shown in thick solid line in Fig. 57.

The variables source_lo and source_up have to be fixed. The existing sources can be obtained in the following way: 
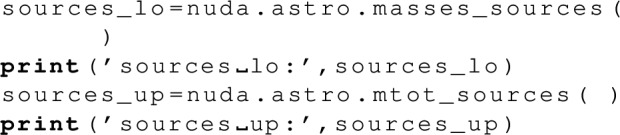


Once the variables source_lo and source_up are chosen in the previous list, the call for the mass observation is performed in the following way: 
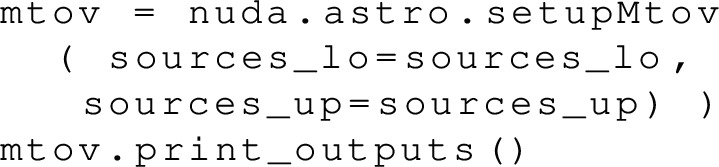


The mass distributions are represented in Fig. 57. It illustrates the impact of the different measurements for the lower and upper boundaries of the TOV mass.

The attributes are given in the form of Numpy arrays: mtov.mass is a vector with a set of masses for which values the distribution is provided, mtov.proba_lo[i] provide the lower mass distribution of probabilities corresponding to each of the source given in the array sources_lo with index i, see Eq. (86), mtov.proba_up[j] provide the upper mass distribution of probabilities corresponding to each of the source given in the array sources_up with index j, see Eq. (88), and finally, mtov.proba_tot contains the product of lower and upper probabilities, see Eq. (90).

### Mass-radius measurement by X-ray emission

The NICER observatory [225] is providing a large amount of data which are analyzed to determine accurately the mass and the radius of pulsars. A summary of these masses and radii measurements is shown in Table 10.

**Table 10:** Observational radii and masses for different pulsars. *C* stands for compactness and is written when it is provided by the authors. We also complete the table with our calculation of the compactness obtained from the reported distribution of masses and radii (in bold)

Source	Obs	Telescope	*R*	*M*	*C*	References
			[km]	[$$M_{\odot }$$]		
PSR J0030+0451	1	NICER	$13.02^{+1.24}_{-1.06}$	$1.440^{+0.150}_{-0.140}$	$0.163^{+0.008}_{-0.009}$	[226]
	2	NICER	$12.71^{+1.14}_{-1.19}$	$1.340^{+0.150}_{-0.160}$	$0.156^{+0.008}_{-0.010}$	[227]
	3	NICER	$14.44^{+0.88}_{-1.05}$	$1.700^{+0.180}_{-0.190}$	$0.179^{+0.011}_{-0.022}$	[228]
	4	NICER	$11.71^{+0.88}_{-0.83}$	$1.400^{+0.130}_{-0.120}$	$0.1773^{+0.0056}_{-0.0074}$	[228]
	av		$12.89\pm 1.17$	$1.390\pm 0.158$	$0.159\pm 0.009$	
MSP J0740+6620	1	NICER	$13.70^{+2.60}_{-1.50}$	$2.080^{+0.070}_{-0.070}$		[229]
	2	NICER	$12.39^{+1.30}_{-0.98}$	$2.072^{+0.067}_{-0.066}$		[230]
	3	NICER	${12.49}^{+1.28}_{-0.88}$	$2.073^{+0.069}_{-0.069}$		[231]
	av		$13.10\pm 1.66$	$2.075\pm 0.068$		
PSR J0437-4715	1	NICER	$11.360^{+0.945}_{-0.629}$	$1.418^{+0.037}_{-0.037}$	$0.1847^{+0.0097}_{-0.0143}$	[232]

The complete list of available sources is given with the following instructions: 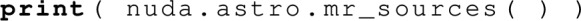


The list of observations, see Table 10, for a given source is given with the following instruction: 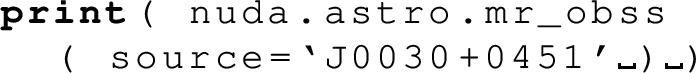


The object mr is instantiated in the following way: 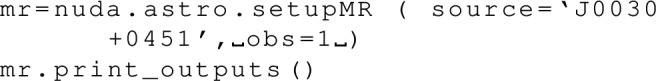 where the variables source and obs are chosen in the previous list, see also Table 10. We now provide more details for each of the sources encoded in the nuda toolkit.

source=‘J0030+0451’.

Observed mass and radii for source J0030+0451 obtained from different analyses of the observational data (obs=1, 2, 3, or 4), see Table 10 for more details.

obs= 1 is the result of the fit to NICER with three oval hot-spots from Ref. [226],

obs= 2 is the result of the fit to NICER for ST+PST hot-spot model from Ref. [227].

obs= 3 is the result of the fit to joint NICER and XMM-Newton data for PDT-U hot-spot model from Ref. [228].

obs= 4 is the result of the fit to joint NICER and XMM-Newton data for ST+PDT hot-spot model from Ref. [228].

source=‘J0740+6620’.

Observed mass and radii for source J0740+6620 obtained from different analyses of the observational data (obs=1, 2 or 3), see Table 10 for more details.

obs= 1 is the result of the fits to NICER and XMM-Newton Data with a Parameterized Normalization for the XMM-Newton Calibration obtained in Ref. [229],

obs= 2 is the result of the fit to NICER considering ST-U model with a conditional constraint on NICER XTI pulse-profile modeling, joint NANOGrav and CHIME/Pulsar wideband radio timing, and XMM EPIC spectroscopy obtained in Ref. [230].

source=‘J0437-4715’.

Observed mass and radii for source J0437-4715 obtained from different analyses of the observational data (obs=1), see Table 10 for more details.

obs= 1 is the result of the fits to NICER considering the high MULTINEST resolution CST+PDT runs and in the presence of lower and upper background constraints involving the instrument background and 3C50 AGN spectrum obtained in Ref. [232].

The attributes of the object mr are: mr.rad, mr.rad_ sig_lo, and mr.rad_sig_up for the radius and its lower and upper uncertainties, as well as mr.mass, mr.mass_ sig_lo, and mr.mass_sig_up for the mass measurement.

**Fig. 58:**
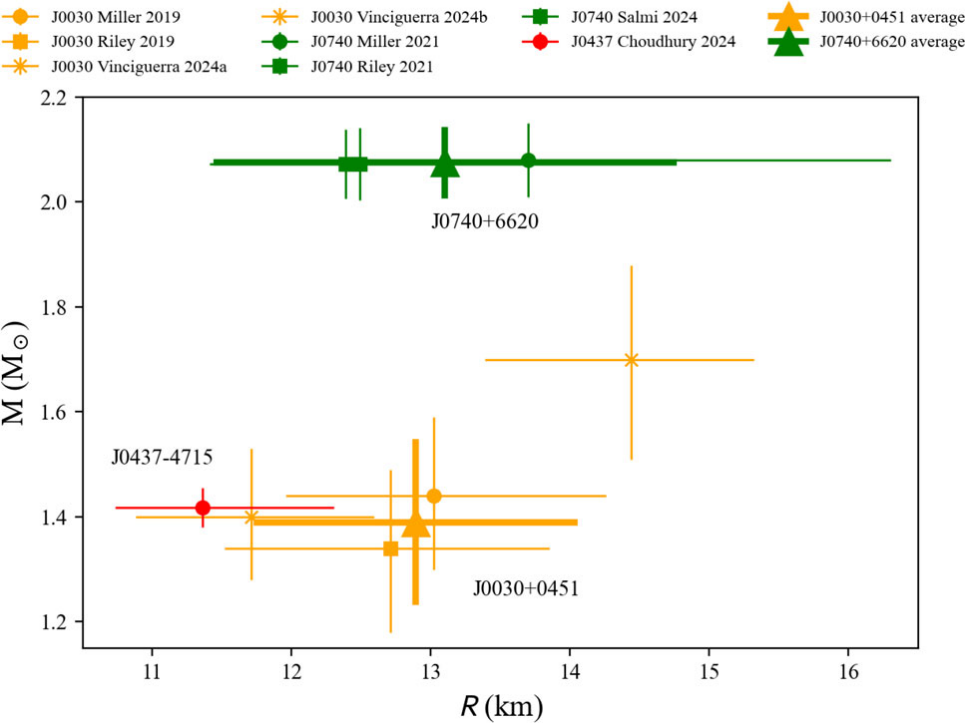
Observational measurement of masses and radii by the NICER observatory [225]. Figure generated with astro_setupMR_plot.py

**Table 11:** The lower and upper boundary at 90% CI of the effective tidal deformability obtained from various analyses of GW sources

Source	Hyp	Hypothesis	$M_\textrm{chirp}$ [M$_{\odot }$]	*q*	$\tilde{\varLambda }_{90\%}$	References
GW170817	1	low-spin	1.188$^{+0.004}_{-0.004}$	[0.7:1]	400$^{+400}_{-400}$	[222]
		+ TaylorF2				
GW170817	2	high-spin	1.188$^{+0.004}_{-0.004}$	[0.4:1]	350$^{+350}_{-350}$	[222]
		+ TaylorF2				
GW170817	3	low-spin	1.186735$^{+0.000119}_{-0.000095}$	[0.639:0.982]	222$^{+420}_{-138}$	[239]
		+ TaylorF2				
GW170817	4	low-spin	1.186$^{+0.001}_{-0.001}$	[0.73:1]	300$^{+420}_{-230}$	[223]
		+ PhenomPNRT				
GW170817	5	high-spin	1.186$^{+0.001}_{-0.001}$	[0.53:1]	315$^{+315}_{-315}$	[223]
		+ PhenomPNRT				
	av				355$^{+338}_{-338}$	
GW190425	1	low-spin	1.44$^{+0.02}_{-0.02}$	[0.8:1.0]	300$^{+300}_{-300}$	[224]
GW190425	2	high-spin	1.44$^{+0.02}_{-0.02}$	[0.8:1.0]	550$^{+550}_{-550}$	[224]
	av				425$^{+456}_{-425}$	

The different measurements are represented in Fig. 58, where each color is specific to a given pulsar. For a given source, the different measurements can be averaged together in the following way: 
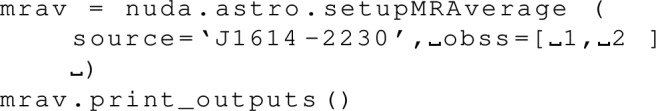
 The attributes are the centroid mrav.mass_cen and the standard deviation mrav.mass_std for the masses and mrav.rad_cen and the standard deviation mrav.rad_std for the radii.

### Tidal deformabilities from gravitational waves

At leading order, the tidal effects are imprinted in the gravitational waveform through the effective tidal deformability $\tilde{\varLambda }$ [233–238], which is defined as

91 $$\begin{aligned} \tilde{\varLambda } = \frac{16}{13} \frac{(12q+1)\varLambda _1+(12+q)q^4\varLambda _2}{(1+q)^5} , \end{aligned}$$

where $\varLambda _1$ and $\varLambda _2$ are the dimensionless tidal deformability for the two merging stars, related to the tidal Love number $k_i$ as,

92 $$\begin{aligned} \varLambda _i=\frac{2}{3} k_i \left( \frac{R_i c^2}{G M_i}\right) ^5 . \end{aligned}$$

$R_i$ and $M_i$ being the radii and the mass if the star *i*, $i=1$, 2. The binary mass ratio is $q=M_2/M_1\le 1$. The chirp mass is

93 $$\begin{aligned} M_\textrm{chirp}=\frac{(M_1 M_2)^{3/5}}{M_\textrm{tot}^{-1/5}} , \end{aligned}$$

with $$M_\textrm{tot}=M_1+M_2$$.

Several analyses have extracted the tidal deformabilities from the GW signal of GW170817 and GW190425. They are reported in Table 11.

The complete list of available sources is given with the following instructions: 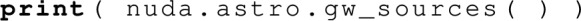


The list of hypotheses for a given source is given with the following instruction: 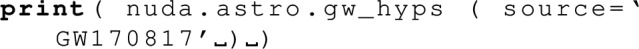


The object gw is instantiated in the following way: 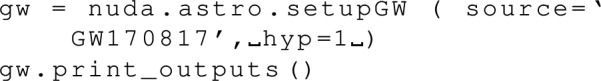 where the variables source and hyp are chosen in the previous list.

We now provide more details about the observed masses available in nuda toolkit. There are several sources, and for some sources, there are several observations providing different values for the observed masses.

source=‘GW170817’.

Observed mass for source GW170817 obtained from different hypotheses (hyp=1, 2, 3, 4 or 5).

hyp=1 low spin prior from Ref. [222], 2 high spin prior from Ref. [222],

hyp=3 from Ref. [239],

hyp=4 low prior prior from Ref. [223], or 5 high spin prior from Ref. [223].

source=‘GW190425’.

Observed mass for source GW170817 obtained from a single hypothesis (hyp=1 or 2).

hyp= 1 low spin prior from Ref. [224], or 2 high spin prior from Ref. [224].

The attributes of the object gw are: gw.mchirp, gw.mchirp_sig_lo, gw.mchirp_sig_up for the chirp mass $M_\textrm{chirp}$, gw.q_lo and gw.q_up for the mass ratio *q*, and gw.lam, gw.lam_sig_lo, and gw.lam_sig_up for the effective tidal deformability $\tilde{\varLambda }$.

**Fig. 59:**
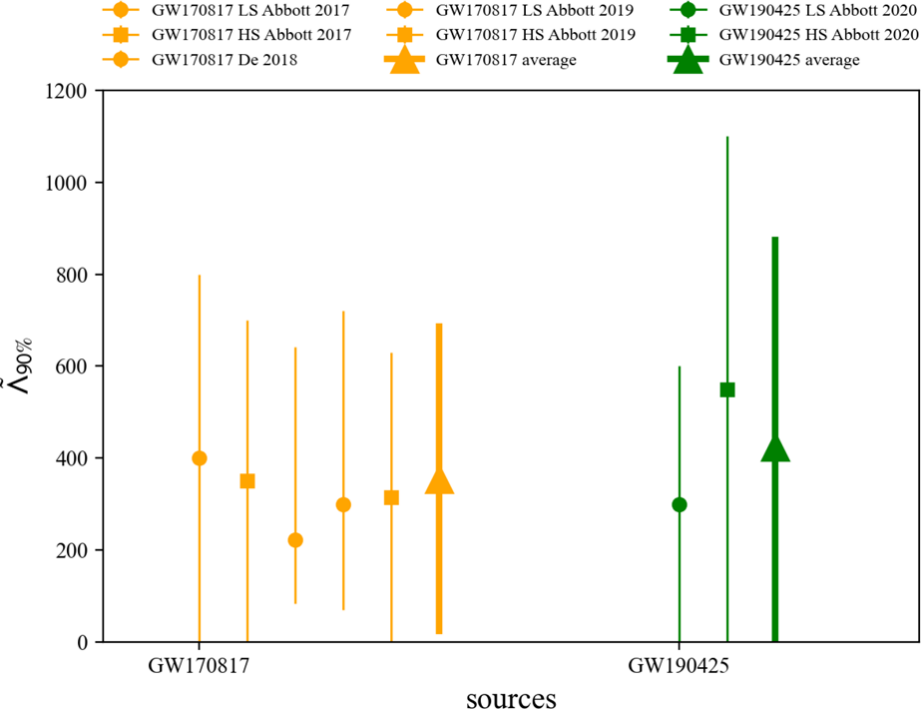
Distribution of effective tidal deformabilities at 90% confidence level for the two gravitational wave source presently known GW170817 and GW190425. Figure generated with astro_setupGW_plot.py

The GW data in the nuda toolkit are shown in Fig. 59 for the two sources GW170817 and GW190425. For a given source, the different measurements can be averaged together in the following way: 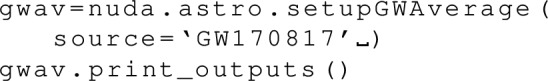 The attributes are the centroid gwav.lam_cen and the standard deviation gwav.lam_std for the effective tidal deformability $\tilde{\varLambda }$.

## Conclusions

In this paper, we have described in detail the use of the nucleardatapy toolkit for uniform matter, nuclear experimental data, correlations, and astrophysical data. The goal of this numerical tool is to provide an easy way to access most of the available theoretical, experimental, and astrophysical data. The amount of data included in the present version of the toolkit represents only a fraction of the data existing in the literature, and we hope that our colleagues will find it an interesting tool to contribute to the database by sharing with us their results and making them easily accessible to the community. Another advantage of the toolkit is that it offers modular functions that average over several predictions, for which the user is free to decide which one to consider. Through the present paper, we have shown at different places in tables and figures how the original data compares to the averaged data, or how to build a reference band from the user’s choice.

This paper illustrates the use of the toolkit for research and provides many examples. For more details, please consult the documentation [2] and the tutorials [3]. The scripts generating the figures shown in this paper are all available in the toolkit, and the GitHub repository [1] is public. All the data provided in this toolkit are accompanied by the proper citations to the original paper, and this toolkit should not supersede them.

In the future, we plan to update the toolkit with newly available data, as well as extend it to new results. We are therefore willing to continue to enrich the toolkit in the future. Any suggestions from the community or requests for extensions are welcome. Any of the co-authors can be contacted for this purpose.

## Data Availability

My manuscript has associated data in a data repository. [Author’s comment: The figures generated during and/or analysed during the current study are available in the https://github.org/jeromemargueron/nucleardatapy/version-1.0/nucleardatapy_samples/figs.]

## Appendix A List of abbreviations

**Table:**

Abbreviation	Meaning
AFDMC	Auxiliary Field Diffusion Monte Carlo
AM	Asymmetric (infinite) Matter
BCS	Bardeen–Cooper–Schrieffer
BHF	Brueckner–Hartree–Fock
DDRH	Density-Dependent Relativistic Hartree
DDRHF	Density-Dependent Relativistic Hartree–Fock
EDF	Energy Density Functional
EFT	Effective Field Theory
EoS	Equation of State
FFG	Free Fermi Gas
GW	Gravitational wave
HFB	Hartree–Fock–Bogoliubov
HIC	Heavy-Ion Collision
MBPT	Many-Body Perturbation Theory
NEP	Nuclear Empirical Parameters
NLEFT	Nuclear Lattice Effective Field Theory
NLRH	Non-Linear Relativistic Hartree
NM	Neutron (infinite) Matter
NR	Non-Relativistic
NRFFG	Non-Relativistic Free Fermi Gas
NS	Neutron Star
N2(3)LO	next-to-next-to-(next-to)-leading order
PVES	Parity-Violating Electron Scattering
QMC	Quantum Monte Carlo
RFFG	Relativistic Free Fermi Gas
RH	Relativistic Hartree or RMF
*r.m.*	rest mass
RMF	Relativistic Mean Field
SM	Symmetric (infinite) Matter

## Appendix B Install nuda toolkit on an iPad

### Appendix B.1 Using a-Shell

The free app a-Shell emulates a terminal for the iOS of your iPad. and can do locally most of the things that a terminal can do. It can be downloaded directly from the App Store. Once installed, you can launch it and write in the prompt:  A list of commands and some explanation are provided on the terminal screen.

To install nucleardatapy, write in a-Shell:  Maybe you will have to install some additional libraries:

You can start by importing the toolkit as:

You can launch any samples.

Alternatively, you can clone the git repository with the following command: https://github.com/jeromemargueron/nucleardatapy.git

Note: a-Shell has a Vim editor. Press i to write into the file, and if you do not have an Esc key on your keyboard, then use cmd+., and write :wq to write and quit.

### Appendix B.2 Using Carnets-Plus

The iPad app Carnets-Plus is a free app providing a Jupyter Notebook running locally on an iPad.

To start, create a new File will create a Jupyter notebook. Then install the toolkit as before:  You can start by importing the toolkit as:

You can now start running the toolkit, and follow, for instance, the instructions given in this paper.

## Footnotes

1 A list of all abbreviations is provided in Appendix A.

2 New microscopic models have recently been added to the toolkit: many-body perturbation theory ’MBPT’, self-consistent Green’s functions ’SCGF’, and coupled-cluster ’CC’ from Refs. [17–19] in 2024 and self-consistent Green’s functions ’SCGF’ From Ref. [20] in 2020.

## Code Availability

My manuscript has associated code/software in a data repository. [Author’s comment: The code/software generated during and/or analysed during the current study is available in the nucleardatapy repository, https://github.org/jeromemargueron/nucleardatapy.]
